# Reduction of Substituted Benzo-Fused Cyclic Sulfonamides with Mg-MeOH: An Experimental and Computational Study

**DOI:** 10.1021/acs.joc.2c01169

**Published:** 2022-09-01

**Authors:** Aisha Khalifa, Robert Redmond, Goar Sánchez-Sanz, Paul Evans

**Affiliations:** Centre for Synthesis and Chemical Biology, School of Chemistry, University College Dublin, Dublin D04 N2E5, Ireland

## Abstract

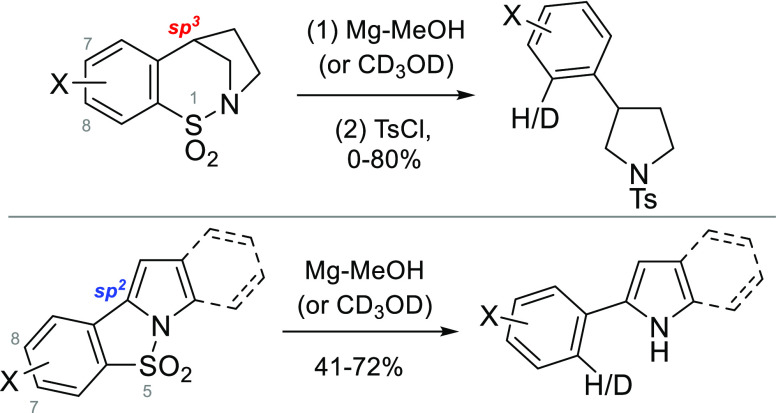

A study involving the use of Mg-MeOH for the double reductive cleavage of both N–S and C–S bonds in a series of 11 benzo-fused cyclic sulfonamides is reported. Examples where the sulfonamide nitrogen atom is part of a pyrrolidine ring effectively undergo reduction, as long as a methoxy substituent is not *para*-positioned in the aromatic ring, relative to the sulfonyl group. In contrast, if the nitrogen atom is contained within an aromatic ring (pyrrole or indole), the presence of a *para*-methoxy substituent does not prohibit reduction. If deuterated methanol is used, aromatic *ortho-*deuterium incorporation was observed. To better understand how structure affects reactivity, density functional theory calculations were performed using three functionals. Results using CAM-B3LYP were found to best correlate with experimental observations, and these demonstrate the impact that the different aromatic substitution patterns and types of N-atom have on the lowest unoccupied molecular orbital (LUMO) energies and adiabatic electron affinities.

## Introduction

**Scheme 1:**
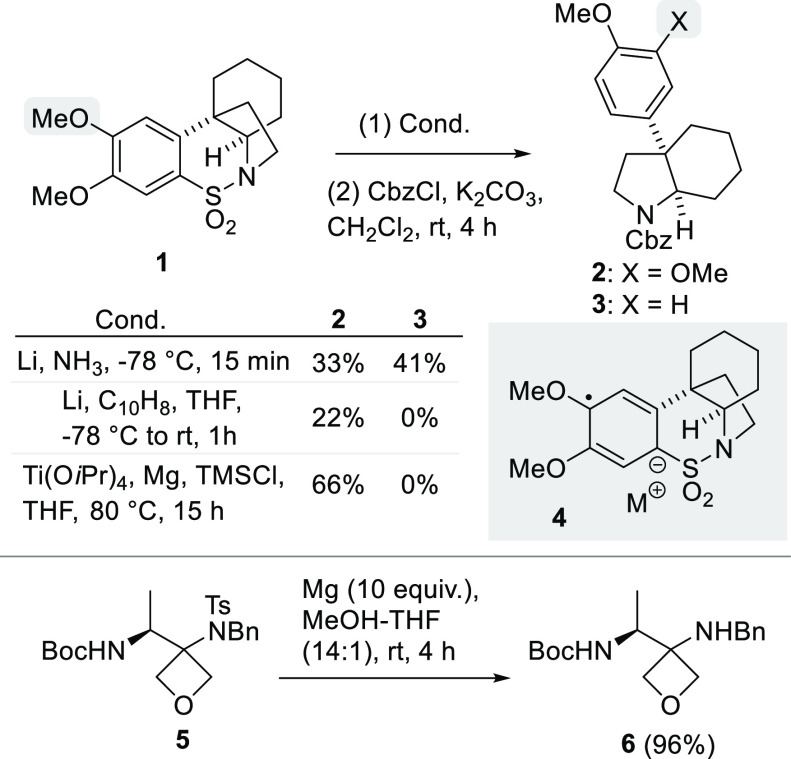
Double Reduction of Cyclic Sulfonamides for the Preparation of Aryl Ring-Containing Amines and the Representative Use of Mg-MeOH for the Deprotection of *N*-Sulfonyl Groups (M = Metal)

For several years, we have worked on the reductive cleavage of cyclic sulfonamides (sultams) in a process in which both the N–S and C–S bonds are replaced.^1−7^ In most of the examples studied, an aromatic moiety flanks the sulfonyl group (e.g., compound **1**, Scheme 1), and the double reductive process generates aromatic ring-containing amines (of type **2**). In terms of a general approach for the synthesis of this type of amine, the inclusion of the sulfonyl group both protects the amino group and strategically delivers the aromatic unit. With specific reference to the synthesis of compound **2**, good conversion was obtained with Li/NH_3_; however, this method was hampered by a partial loss of the methoxy group *para*- to the sulfonyl group, which led to compound **3**.^5^ Although compounds **2** and **3** were separable as their *N*-Cbz derivatives, the formation of **3** hampered the application of this chemistry for the synthesis of the target *Sceletium* and *Amaryllidaceae* alkaloids.^5^ It was reasoned that the undesired process occurs via a radical anion intermediate formed from single electron transfer (SET) of type **4**. Support for this hypothesis came from the finding that the formation of **3** can be avoided if aprotic conditions (lithium naphthalenide or low-valent titanium) are employed.^7^

The combination of magnesium and methanol is a well-recognized reductant, and in general, the lower cost and operational simplicity of this system make its use attractive compared with alternative choices that are able to mediate similar reductive processes.^8^ In addition, numerous examples exist, demonstrating that this mixture can efficiently cleave arenesulfonamide functionality to reveal the amine of interest.^9^ For example, *N*-tosylsulfonamide **5** was efficiently converted into protected diamine **6** in a process in which the benzyl, carbamate, and oxetane units survive (Scheme 1).^10^ In this current work, the utilization of the Mg-MeOH system as a means to reductively excise the sulfonyl group from a series of cyclic sulfonamides is described.

## Results and Discussion

**Scheme 2:**
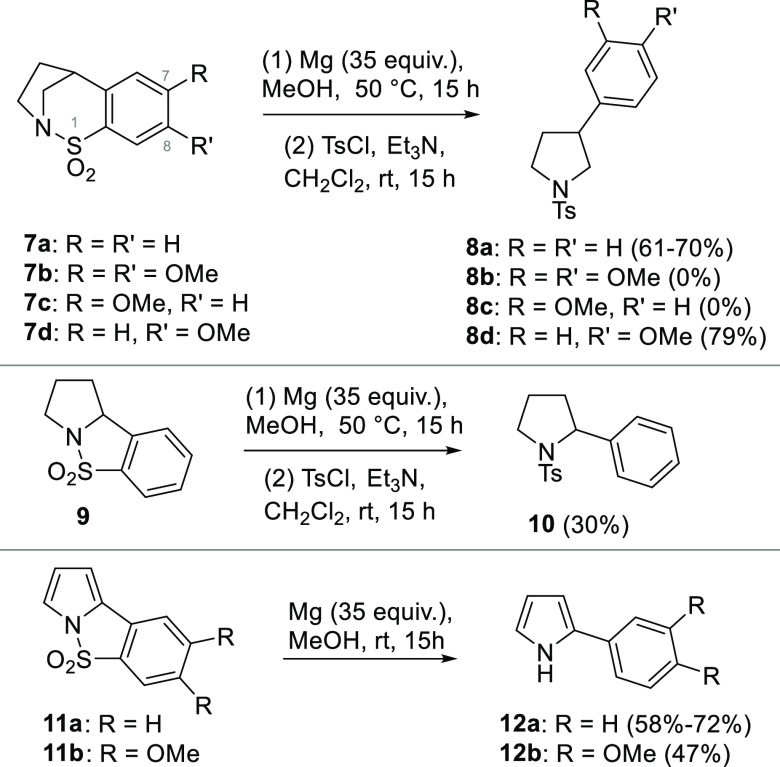
Mg-MeOH Reduction of Benzo-Fused Cyclic Sulfonamides for the Synthesis of 3- and 2-Aryl Pyrrolidines and Pyrroles

Based on our interests in the chemistry of cyclic aromatic sulfonamides, we became keen to study if the Mg-MeOH method would lead not only to N–S bond cleavage (as previously documented in numerous examples) but whether, under these conditions, the aromatic C–S bond would also be reductively cleaved. To this end, we initially revisited our previously studied benzo-fused cyclic sulfonamides **7a** and **7b**, which can be conveniently accessed via an intramolecular Heck process.^11,12^ As shown in Scheme 2, marked differences in reactivity were observed with Mg-MeOH based upon the presence and relative position of the methoxy substituents.

Unsubstituted sulfonamide **7a** undergoes reduction with Mg in MeOH to generate **8a** in reasonable good yield after conversion to its toluene sulfonamide derivative for purification and characterization purposes (Scheme 2). This process proceeds most effectively if elevated temperatures and an excess of activated Mg powder, or turnings, were used. However, when dimethoxy-substituted compound **7b** was subjected to identical conditions, no conversion took place, and only recovered starting material was observed. Since **7b** is only partially soluble in MeOH at 50 °C, this process was also performed with THF as a cosolvent, and a similar lack of reactivity was observed. We speculated that the dimethoxy substituents in the aromatic ring serve to make the initial addition of an electron to **7b** more difficult. Accordingly, isomeric monomethoxy-substituted cyclic sulfonamides **7c** and **7d** were prepared and studied.^7,12^ Unlike **7b**, the 7-methoxy isomer, **7c**, proved to be freely soluble in MeOH at room temperature, but as was observed with **7b**, none of the reactions of interest took place, and the starting material was recovered in quantitative amounts. In contrast, the 8-isomer, **7d**, in which the methoxy substituent is *meta-*positioned relative to the sulfonyl group, gave **8d** in a very good yield. These results clearly demonstrate that with Mg-MeOH, the ease of sulfonamide reduction is dependent on the nature of and the positioning of the substituents relative to the excised sulfonyl moiety. Compound **9**, the 2-isomer of **7a**, also underwent the same type of reaction to generate **10**, although the isolated yield was moderate, due in part, to some unreacted **9** (∼15%).

To further explore the scope of the Mg-mediated double reduction, new cyclic sulfonamide substrates were sought. To this end, for the first time, pyrrole-based substrates **11a**–**c** were considered in this type of reduction reaction. These compounds can be readily accessed from a dihydropyrrole-oxidation sequence followed by a Pd-mediated sp^2^–sp^2^ coupling.^13^ Treatment of **11a** with Mg-MeOH, under identical conditions to those successful for saturated substrate **7a**, gave 2-phenyl pyrrole **12a** in good yield (Scheme 2). Subsequently, it was shown that this process also proceeds efficiently at lower temperatures than the saturated counterparts **7a** and **7d**. This suggested that the biaryl group was responsible for the enhanced reactivity of **11a** compared to **7a**. Therefore, we were interested to see if the combination of the dimethoxy substitution pattern, which led to no reaction in the case of **7b**, would be processed—as long as the dimethoxy aromatic unit was part of a biaryl system. Thus, **11b** was submitted to the reaction, and we were pleased to observe that **12b** was formed in reasonable yield, supporting the idea that the extended conjugation counteracts the electron-donating nature of the methoxy groups. In relation to the yield of **12b**, the conversion in this reaction is high; however, the electron-rich pyrrole product proved to have limited stability during purification by silica gel flash column chromatography.

**Scheme 3:**
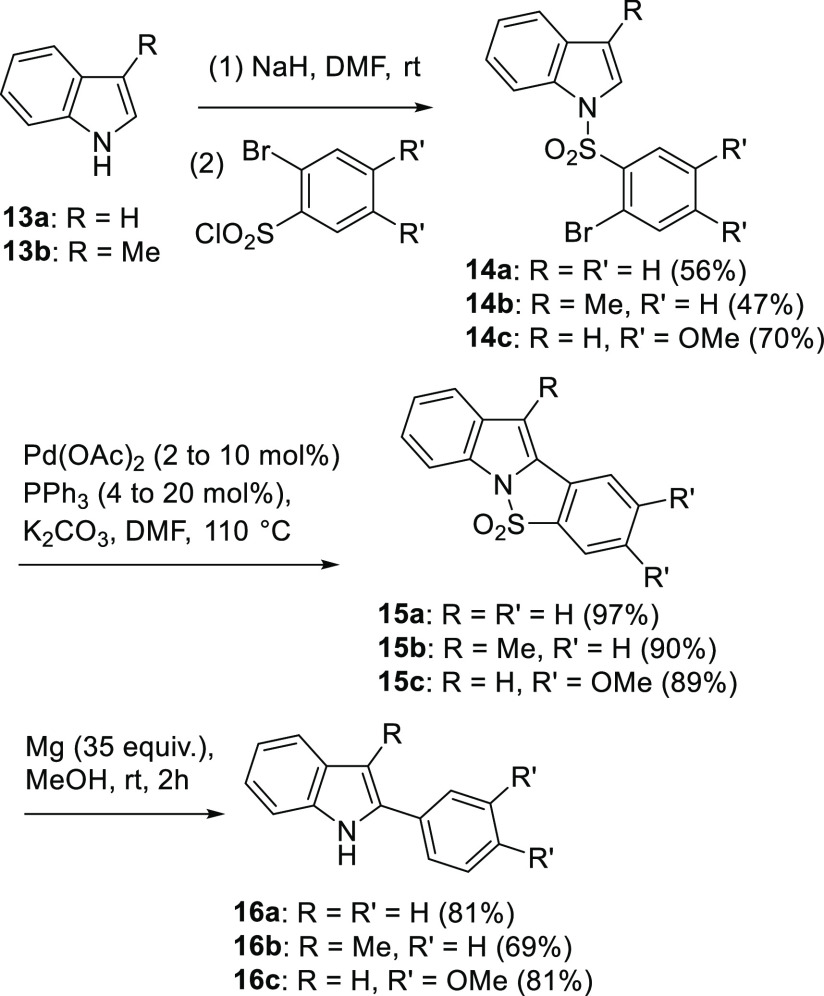
Synthesis of Cyclic *N*-Sulfonyl Indoles **15a–c** and Their Mg-MeOH-Based Reduction for the Preparation of 2-Aryl Indoles **16a–c**

As shown in Scheme 3, an identical overall process was achieved for the first time with indole-based cyclic sulfonamides **15a**–**c**. These cyclic sulfonamides were prepared by a palladium-mediated sp^2^–sp^2^-coupling sequence.^14^ On treatment with Mg-MeOH at room temperature, good yields of the corresponding 2-aryl indoles **16a–c** were observed.

Similarly to pyrrole **11b**, and unlike its saturated pyrrolidine counterpart **7b**, dimethoxy-substituted sulfonamide **15c** underwent the double reduction process of interest generating **16c**. Small amounts of less-polar side products that appear to be over-reduced dihydroindoles were detected in these reactions.

**Scheme 4:**
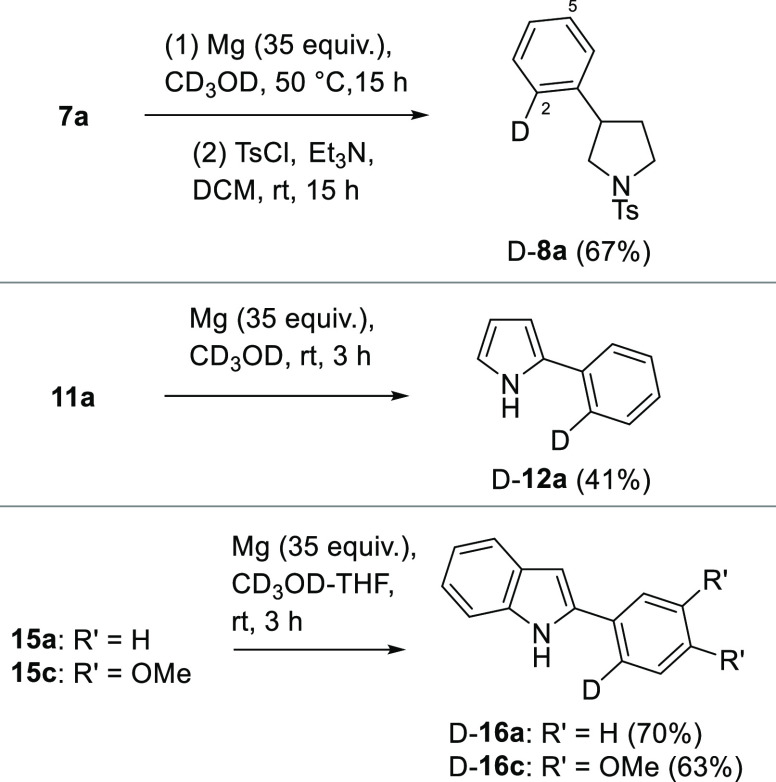
Synthesis of Compounds d-**8a**, d-**12a**, and d-**16a** and **16c** Using the Mg-Deuteration Process

The use of methanol as a solvent, mediator for the transfer of electrons, and a proton source offers the opportunity to replace it in these reactions with CD_3_OD. As shown in Scheme 4, when CD_3_OD was used in the reaction of **7a** with Mg, selective incorporation of deuterium in the 2-position was observed. After tosylation, d-**8a** was isolated in good yield. The incorporation of the single deuterium atom was supported by proton NMR spectroscopy, where the loss of a proton signal in comparison to the spectrum from **8a** was observed, and in carbon NMR spectroscopy, where a triplet was evident at ∼126.5 ppm.

This finding was not completely anticipated since, based on our hypothesis for methoxide loss (see Scheme 1, proposed intermediate **4**), it was felt that partial incorporation of deuterium in the 5-position was possible. In the event, this was not detected in any appreciable amount. This selective deuteration process was extended with the pyrrole (**11a**) and the indole-based cyclic sulfonamides **15a** and **15c**, which led to the formation of the 2-deuterio compounds d**-12a**, d-**16a**, and d-**16c** (Scheme 4). As observed previously, yields for the indoles in this double reduction process were higher than that observed for the pyrrole, a finding attributed to the improved stability of the reaction products.

**Figure 1:**
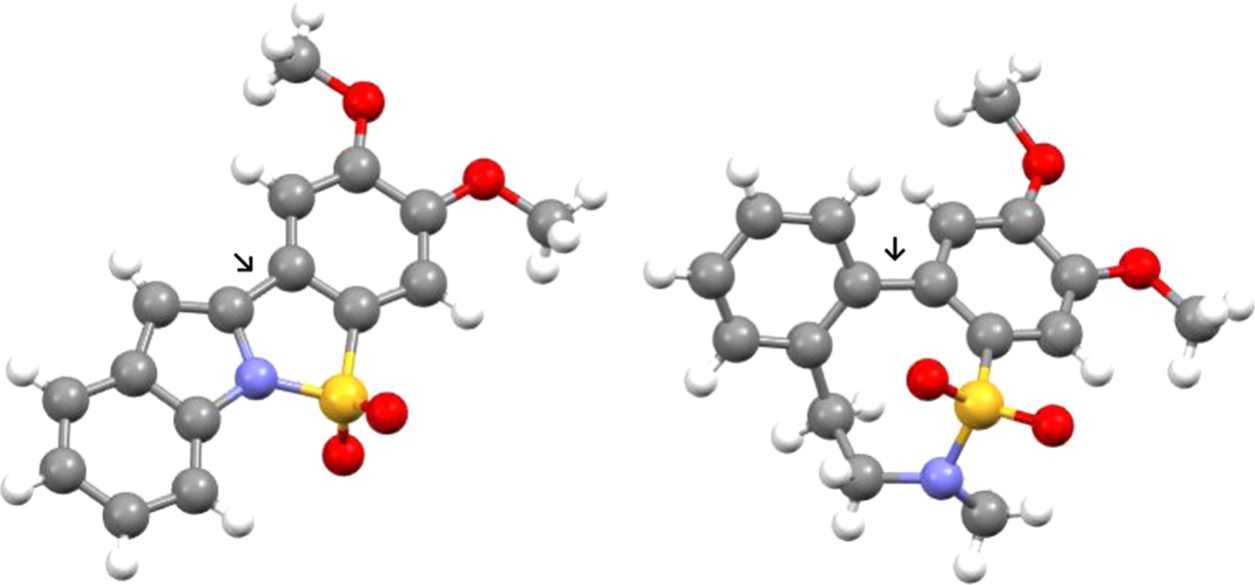
Comparison of single-crystal X-ray structures for dimethoxy-containing biaryl sulfonamides **15c** and **17**. Torsion angles at biaryl bonds indicated by arrows are 3.40 and 53.21°, respectively.^16^

**Scheme 5:**
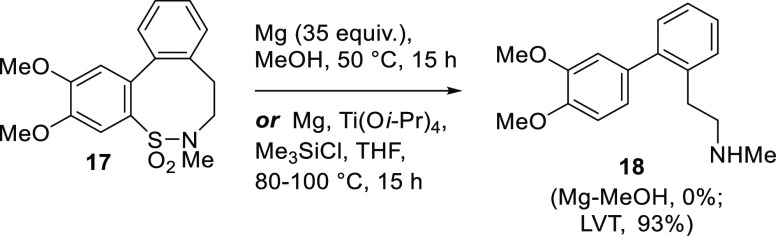
Attempted Double Reduction for the Synthesis of 2-(3,4^′^-Dimethoxy-[1,1^′^-biphenyl]-2-yl)-*N*-Methylethanamine **18**

In relation to the reactivity patterns observed with Mg-MeOH, when the electron releasing methoxy substituent is *para-*positioned relative to the sulfonyl group, the results indicate that the ability of a biaryl unit to overturn the lack of reactivity of the methoxy-substituted cyclic sulfonamide examples depends on conjugation (i.e. no reaction is observed for dimethoxy-substituted benzo-fused cyclic sulfonamide **7c**, whereas similarly substituted pyrrole **11b** and indole **15c** compounds react). Further evidence for this was found when **17**(15) was studied. This biaryl sulfonamide fails to undergo any reaction with Mg-MeOH at either rt or 50 °C (Scheme 5). In this case, X-ray crystallography^16^ demonstrates that the biaryl axis is staggered and the two aromatic systems are, therefore, not subject to direct conjugation, unlike pyrrole **11b** and indole **15c** (see comparative X-ray crystallographic structures in Figure 1).

Compound **17** does, however, undergo the double reductive process of interest with Okamoto’s low-valent titanium (LVT) reaction conditions,^17^ and it was found that **18** can be isolated in an unpurified yield of 93%.

Our rationale for the substituent effects observed during the described Mg-MeOH reduction reactions concerns the relative energies of the lowest unoccupied molecular orbitals (LUMOs) for the differently substituted benzo-fused sulfonamides. We hypothesized that electron addition will be more difficult when the electron-releasing methoxy substituents were *para*-positioned relative to the sulfonyl group. This, coupled with the comparative instability of the initially formed radical anion, resulting from the addition of a single electron into the LUMO (e.g., structure **4**, Scheme 1), likely has the strongest influence on reaction outcome. To gain insight into this interplay, geometry optimizations using density functional theory (DFT) have been carried out for compounds **7a**–**d**, **11a–b**, **15c**, and **17**. To do this, three functionals CAM-B3LYP, WB97XD, and M06-2X were used with def2TZVP as the basis set.^18−20^ Frequency calculations have been carried out to verify the nature of the minima, and for each compound, true minima have been identified with no imaginary frequencies. According to these calculations, the CAM-B3LYP results most closely match the experimental outcomes (additional data obtained using the other functionals can be found in the Supporting Information). As can be seen in Table 1, moving from **7a** to **7b**, the relative energies of the LUMO are raised (entries 1 and 2). On forcing an electron into the molecule to form a radical anion (adiabatic electron affinities), results indicate that compound **7b** is also less willing to accommodate this extra charge. A similar trend is observed for isomeric monomethoxy-substituted compounds **7c** and **7d**. Compound **7d**, in which the methoxy substituent is *meta-*positioned relative to the sulfonyl group, possesses both a lower lying LUMO and is better able to accommodate the added electron than its *para*-methoxy counterpart, **7c** (entries 3 and 4).

**Table 1:** LUMO Orbital Energies in au, and Adiabatic Electron Affinities in kJ mol^–1^, at CAM-B3LYP/def2TZVP Levels for Compounds **7a**–**d**, **11b**, **15c**, and **17**

entry	starting material	yield (%)^a^	LUMO^b^	adiabatic electron affinities (EA)^b^
1	**7a**	71	–0.0002	–6.55
2	**7b**	0	0.0156	4.05
3	**7c**	0	0.0101	9.80
4	**7d**	79	0.0007	–8.69
5	**11b**	47	–0.0012	–6.45
6	**15c**	81	–0.0219	–67.69
7	**17**	0	0.0076	–7.24

a Observed isolated yields, see scheme 2–5.

b Data calculated using CAM-B3LYP.

The effect of the additional conjugation present in the pyrrole (**11b**) and indole (**15c**) dimethoxy-containing biaryl cyclic sulfonamides is also evident (entries 5 and 6). In comparison to **7b** (entry 2), both **11b** and **15c** present a lower energy LUMO and are also significantly better able to accommodate the additional electron following single-electron transfer. Finally, as shown in entry 7, dimethoxy-containing biaryl compound **17** presents its LUMO at a comparatively higher energy than its planar pyrrole and indole analogues; a finding which is consistent with its lack of reactivity under the Mg-MeOH conditions. However, based on the adiabatic electron affinity values obtained for this compound, one might anticipate the successful formation of the intermediate required to react further. This hints that the relative energies of the LUMO orbitals for the compounds studied in this report may be the crucial determiner of a successful reaction under these Mg-MeOH conditions.

## Conclusions

In conclusion, the use of the Mg-MeOH combination for double reduction of cyclic sulfonamides has been demonstrated. This procedure can provide a complementary way to reductively excise the sulfonyl group in certain cyclic sulfonamides and, unlike previous reports for this type of reaction,^1−7^ does not require the use of gaseous ammonia or anhydrous conditions. Notably, the use of deuterated methanol specifically incorporated deuterium in place of the C–S bond. However, results indicated that the substitution pattern on the aromatic ring is a crucial factor for the success of this process. Thus, for situations where the benzylic carbon atom is sp^3^ hybridized, compounds with a *para*-methoxy group to the sulfonyl group resist reduction (i.e., compounds **7b** and **7c**). However, for compounds where the carbon atom attached to the benzo group is sp^2^ hybridized, reduction proceeds irrespective of the substitution in the benzo-fused aromatic ring (i.e., compounds **11b** and **15c**). This different pattern of reactivity was probed computationally, and calculations suggest that the relative energies of the LUMOs dictate whether the reactions under the Mg-MeOH conditions can occur. This appears to be more significant than the relative energies of the radical anion formed following SET.

## Experimental Section

### General Directions

Reagents were obtained from commercial suppliers and were used without further purification. CH_2_Cl_2_ was dried over activated 4 Å molecular sieves. Air- and moisture-sensitive experiments were performed using a high vacuum Schlenk line. Oxygen-free, anhydrous nitrogen was obtained from BOC gases. Flash column chromatography was performed using flash silica 60 Å (230–400 mesh) 9385 supplied by Merck. Thin-layer chromatography was performed on silica-coated aluminum sheets (60 F_254_) supplied by Merck. Compounds were visualized with UV light and aqueous potassium permanganate, followed by heating. Melting points were recorded on a Gallenkamp electrothermal melting point apparatus. Infrared spectra were recorded on a Bruker α FTIR spectrometer. ^1^H and proton decoupled ^13^C NMR spectra were recorded on a Varian Unity 400 MHz system spectrometer. Chemical shifts are quoted in parts per million (ppm) relative to the internal standard reference tetramethylsilane or the residual protonated solvent. Coupling constants (*J*) are quoted in Hertz and corrected to the nearest 0.5 Hz. High-resolution mass spectra were recorded on a VG analytical 70-E mass spectrometer time-of-flight analyzer. X-ray diffraction data for compounds **15c** and **17** were collected on a Rigaku XtaLab SuperNova X-ray diffractometer. Cyclic sulfonamide substrate compounds **7a–7d**,^1,12^**9**,^1^**11a–b**,^13^ and **17**(15) were available from published procedures.

### General Procedure for the Mg-MeOH-Mediated Sulfonamide Double Reduction

The sulfonamide substrate (0.15–1.05 mmol, 1 equiv) was dissolved in MeOH (specific amount depending on solubility). Oven-dried Mg (35 equiv) [either Mg ribbon or powder may be successfully used] was added along with a crystal of iodine, and the mixture was either stirred at room temperature or 50 °C (oil bath temperature) for the specified reaction period. Sat. NH_4_Cl solution (∼10 mL) was added, and the mixture was extracted with EtOAc (3 × ∼15 mL). The combined extracts were dried over anhydrous MgSO_4_. Filtration followed by solvent removal under reduced pressure gave the crude amine or pyrrole/indole. The amines were converted to the corresponding sulfonamides and purified by chromatography, whereas the pyrrole/indoles were directly purified by chromatography. Note that deuteration experiments were performed with commercial CD_3_OD under a N_2_ atmosphere.

### 3-Phenyl-1-tosylpyrrolidine **8a**(1)

As described above, Mg powder (0.723 g, 29.75 mmol, 35 equiv) and crystal of iodine were added to a solution of the cyclic sulfonamide **7a** (0.18 g, 0.86 mmol, 1 equiv) in MeOH (10 mL). The mixture was heated and stirred at 50 °C (oil bath temperature) for 15 h. On cooling, the reaction was diluted with CH_2_Cl_2_ (15 mL) and poured into 0.5 M HCl (15 mL). The organic layer was washed with 1 M NaHCO_3_ (2 × 20 mL) and brine and then dried over anhydrous MgSO_4_. Filtration followed by solvent removal under reduced pressure afforded the crude amine. A solution of the crude amine in CH_2_Cl_2_ (10 mL) was treated with Et_3_N (0.24 mL, 2.3 mmol, 2 equiv) and TsCl (0.174 g, 0.91 mmol, 1.1 equiv) at 0 °C. Stirring was continued for 15 h, and the reaction gradually warmed to room temperature. Silica (ca. 2.0 g) was added to the reaction mixture, and the solvent was removed under reduced pressure. Purification by flash chromatography (*c*-Hex-EtOAc; 3:1) gave **8a** (179 mg, 70%) as a colorless solid. M.P. 65 °C. *R*_f_ = 0.3 (*c*-Hex-EtOAc; 3:1). ^1^H NMR (400 MHz, CDCl_3_): δ 7.77 (d, 2H, *J* = 8.0 Hz) 7.35 (d, 2H, *J* = 8.0 Hz), 7.30–7.18 (m, 3H), 7.11 (d, 2H, *J* = 7.0 Hz), 3.78–3.70 (m, 1H), 3.54 (ddd, 1H, *J* = 10.0, 8.5, 3.5 Hz), 3.37 (dd, 1H, *J* = 10.0, 7.0 Hz), 3.29–3.18 (m, 2H), 2.46 (s, 3H), 2.25–2.17 (m, 1H), 1.94–1.82 (m, 1H). Data are consistent with the literature.^1^

### 3-(4-Methoxyphenyl)-1-tosylpyrrolidine **8d**(1,7)

Under N_2,_ Mg powder (203 mg, 8.35 mmol, 35 equiv) and a crystal of iodine were added to a solution of the cyclic sulfonamide **7d** (50 mg, 0.21 mmol, 1 equiv) in MeOH (5 mL). The mixture was heated and stirred at 50 °C for 15 h. The resulting suspension was cooled, and solid NH_4_Cl (ca. 1.0 g) was added and diluted with CH_2_Cl_2_ (15 mL). A solution of 1 M NaOH (10 mL) was added (until the pH was 12) and stirred for 20 min. The resultant aqueous layer was extracted with CH_2_Cl_2_ (4 × 25 mL). The combined organic layers were dried over anhydrous MgSO_4_. Filtration followed by solvent removal under reduced pressure afforded the crude amine. A solution of the resultant crude amine in CH_2_Cl_2_ (10 mL) was treated with Et_3_N (0.07 mL, 0.65 mmol, 2 equiv) and TsCl (46 mg, 0.239 mmol, 1 equiv) at 0 °C. Stirring was continued for 15 h, and the reaction gradually warmed to room temperature. Silica (ca. 2.0 g) was added to the reaction mixture, and the solvent was removed under pressure. Purification by flash column chromatography (*c*-Hex-EtOAc; 2:1) gave **8d** (55 mg, 79%) as a light-yellow colored viscous oil. *R*_f_ = 0.15 (*c*-Hex-EtOAc; 4:1). *v̅*_max_ 3054, 2958, 2927, 1599, 1492, 1454, 1436, 1340, 1264, 1157, 816, 780, 731, 699, 661, 590, 547 cm^–1^. ^1^H NMR (400 MHz, CDCl_3_): δ 7.78 (d, 2H, *J =* 8.0 Hz), 7.37 (d, 2H, *J =* 8.0 Hz), 7.05 (d, 2H, *J =* 8.0 Hz), 6.83 (d, 2H, *J =* 8.0 Hz), 3.80 (s, 3H), 3.74–3.69 (m, 1H), 3.57–3.51 (m, 1H), 3.40–3.32 (m, 1H), 3.25–3.13 (m, 2H), 2.47 (s, 3H), 2.15–2.23 (m, 1H), 1.89–1.78 (m, 1H). Data are consistent with the literature.^1^

### 2-Phenyl-1-tosylpyrrolidine **10**(1)

As described above, Mg powder (0.896 g, 36.79 mmol, 35 equiv) and crystal of iodine were added to a solution of the cyclic sulfonamide **9** (0.220 g, 1.05 mmol, 1 equiv) in MeOH (12 mL). The mixture was heated and stirred at 50 °C (oil bath temperature) for 15 h. On cooling, the reaction was diluted with CH_2_Cl_2_ (20 mL) and poured into 0.5 M HCl (20 mL). The organic layer was washed with 1 M NaHCO_3_ (2 × 20 mL) and brine and then dried over anhydrous MgSO_4_. Filtration followed by solvent removal under reduced pressure afforded the crude amine. A solution of the crude amine in CH_2_Cl_2_ (10 mL) was treated with Et_3_N (0.14 mL, 1.4 mmol, 2 equiv) and TsCl (0.160 g, 0.84 mmol, 1.2 equiv) at 0 °C. Stirring was continued for 15 h, and the reaction gradually warmed to room temperature. The reaction mixture was diluted with 1 M HCl (10 mL). The aqueous layer was separated and washed with CH_2_Cl_2_ (2 × 10 mL). Silica (ca. 2.0 g) was added to the combined organic layers, and the solvent was removed under reduced pressure. Purification by flash chromatography (*c*-Hex-EtOAc; 3:1) gave **10** (95 mg, 30%) as a colorless solid. M.P. 84–86 °C. *R*_f_ = 0.5 (*c*-Hex-EtOAc; 4:1). ^1^H NMR (400 MHz, CDCl_3_): δ 7.67 (d, 2H, *J* = 8.0 Hz), 7.30–7.20 (m, 7H), 4.79 (dd, 1H, *J* = 8.0, 4.0 Hz), 3.64–3.59 (m, 1H) 3.46–3.40 (m, 1H), 2.42 (s, 3H), 2.04–1.95 (m, 1H), 1.91–1.78 (m, 2H), 1.72–1.63 (m, 1H). Data are consistent with the literature.^1^

### 2-Phenyl-1*H*-pyrrole **12a**(21)

Sulfonamide **11a** (100 mg, 0.49 mmol, 1 equiv) was dissolved in MeOH (10 mL) in a sealed tube with a circular stirrer bar. To this reaction mixture, Mg powder (0.425 g, 17.71 mmol, 36 equiv) was added along with a crystal of iodine. This reaction mixture was sealed and left stirring at 50 °C (oil bath temperature) for 15 h. The resulting reaction mixture was cooled to room temperature, and the reaction was quenched with sat. NH_4_Cl solution (25 mL). Ethyl acetate (20 mL) was added, and the organic layer was removed. The aqueous layer was further extracted with ethyl acetate (2 × 15 mL), and the combined organic layers were dried over MgSO_4_. After filtration, the crude product was purified by column chromatography (*c*-Hex-EtOAc; 9:1) to afford **12a** (50 mg, 72%) as a colorless solid, which became purple over time. M.P. 110–115 °C (decomp). *R*_f_ = 0.25 (*n*-Hex-EtOAc; 2:1). *v̅*_max_ 3431, 3379, 3245, 2923, 1681, 1603, 1494, 1466, 1449, 1410, 1031, 764, 714, 689, 532 cm^–1^. ^1^H NMR (400 MHz, CDCl_3_): δ 8.46 (s(br), 1H), 7.48 (d, 2H, *J* = 8.0 Hz), 7.37 (t, 2H, *J* = 8.0 Hz), 7.19 (t, 1H, *J* = 8.0 Hz), 6.81–6.78 (m, 1H), 6.47–6.45 (m, 1H), 6.31–6.29 (m, 1H). ^13^C{^1^H} NMR (100 MHz, CDCl_3_): δ 132.7, 132.1, 128.8, 126.2, 123.8, 118.8, 110.1, 105.9. HRMS (CI) *m*/*z*: [M + H]^+^ Calcd for C_10_H_10_N 144.0813; Found 144.0808.

### 2-[1-(3,4-Dimethoxyphenyl)]pyrrole **12b**(22)

At rt, a solution of **11b** (25 mg, 0.094 mmol, 1 equiv) in MeOH (3 mL) was treated with Mg ribbon (80 mg, 3.293 mmol, 35 equiv). A crystal of I_2_ was added, and the mixture was stirred at rt for 15 h. Sat. NH_4_Cl solution (20 mL) and EtOAc (15 mL) were added, and the resultant organic layer was removed. The aqueous layer was further extracted with ethyl acetate (2 × 15 mL), and the combined organic layers were washed with H_2_O (25 mL) and dried over MgSO_4_. After filtration and solvent removal under reduced pressure, the crude product was purified by column chromatography (*c*-Hex-EtOAc; 5:1 to 3:1) to afford **12b** (9 mg, 47%) as a viscous oil. *R*_f_ = 0.15 (*c*-Hex-EtOAc; 3:1). ^1^H NMR(400 MHz, CDCl_3_): δ 8.45–8.33 (s(br), 1H), 7.02–6.98 (m, 2H), 6.87 (d, 1H, *J* = 8.0 Hz), 6.84–6.81 (m, 1H), 6.42–6.39 (m, 1H), 6.30–6.24 (m, 1H), 3.92 (s, 3H), 3.89 (s, 3H). ^13^C{^1^H} NMR(100 MHz, CDCl_3_): δ 149.3, 147.8, 132.3, 126.3, 118.3, 116.2, 111.6, 109.9, 108.1, 105.1, 56.0, 55.9.

### 1-[(2-Bromophenyl)sulfonyl]-1*H*-indole **14a**(23)

Indole **13a** (245 mg, 2.09 mmol, 1 equiv) was dissolved in dry DMF (6 mL) and at room temperature treated with 60% w/w NaH in mineral oil (90 mg, 2.25 mmol, 1.1 equiv). After 0.25 h, 2-bromobenzenesulfonyl chloride (535 mg, 2.09 mmol, 1 equiv) was added portionwise. The reaction mixture was stirred for 15 h before EtOAc (15 mL) and H_2_O (15 mL) were added. The resultant aqueous layer was further extracted with EtOAc (2 × 10 mL), and the combined organic extracts were dried of MgSO_4_. Filtration, followed by solvent removal under reduced pressure afforded the crude sulfonamide, which was purified by recrystallization (*c*-Hex-EtOAc), which gave **14a** (396 mg, 56%) as a pale tan solid. M.P. 85–86 °C (*c*-Hex-EtOAc). *R*_f_ = 0.45 (*c*-Hex-EtOAc; 1:1). *v̅*_max_ (dep. CH_2_Cl_2_) 3151, 3118, 3089, 3068, 1573, 1447, 1373, 1263, 1179, 1136 cm^–1^. ^1^H NMR (400 MHz, CDCl_3_): δ 8.09 (dd, 1H, *J* = 7.5, 0.5 Hz), 7.77 (d, 1H, *J* = 4.0 Hz), 7.68–7.65 (m, 2H), 7.59–7.57 (m, 1H), 7.45 (dt, 1H, *J* = 7.5, 0.5 Hz), 7.38 (dt, 1H, *J* = 7.5, 0.5 Hz), 7.25–7.21 (m, 2H), 6.68 (dd, 1H, *J* = 4.0, 0.5 Hz). ^13^C{^1^H} NMR (100 MHz, CDCl_3_): δ 138.1, 136.0, 134.7, 134.5, 131.4, 130.6, 128.2, 127.8, 124.4, 123.3, 121.6, 120.1, 113.0, 107.4. Microanalysis: found C, 50.07; H, 2.76; N, 3.90%; C_14_H_10_NO_2_SBr requires C, 50.00; H, 2.98; N, 4.17%.

### 1-[(2-Bromophenyl)sulfonyl]-3-methyl-1*H*-indole **14b**

3-Methyl indole **13b** (400 mg, 3.05 mmol, 1 equiv) was dissolved in dry DMF (14 mL) and at room temperature treated with 60% w/w NaH in mineral oil (134 mg, 3.35 mmol, 1.1 equiv). After 0.25 h, 2-bromobenzenesulfonyl chloride (780 mg, 3.05 mmol, 1 equiv) was added portionwise. The reaction mixture was stirred for 2 h before EtOAc (25 mL) and H_2_O (25 mL) were added. The resultant aqueous layer was further extracted with EtOAc (2 × 15 mL), and the combined organic extracts were dried of MgSO_4_. Filtration, followed by solvent removal under reduced pressure, afforded the crude sulfonamide, which was purified by filtration through silica gel (*c*-Hex-EtOAc; 6:1) and then recrystallization (*c*-Hex-EtOAc), which gave **14b** (501 mg, 47%) as a colorless solid. M.P. 113–114 °C (*c*-Hex-EtOAc). *R*_f_ = 0.35 (*c*-Hex-EtOAc; 4:1). *v̅*_max_ (dep. CH_2_Cl_2_) 3055, 2987, 1450, 1371, 1265, 1178, 1136 cm^–1^. ^1^H NMR(400 MHz, CDCl_3_): δ 7.96 (dd, 1H, *J* = 7.5, 0.75 Hz), 7.69–7.67 (m, 1H), 7.64 (dd, 1H, dd, *J* = 7.5, 0.5 Hz), 7.51–7.47 (m, 2H), 7.41 (dt, 1H, *J* = 7.5, 0.5 Hz), 7.34 (dt, 1H, *J* = 7.5, 0.75 Hz), 7.26–7.21 (m, 2H), 2.37 (s, 3H). ^13^C{^1^H} NMR (100 MHz, CDCl_3_): δ 138.5 ppm, 135.9, 135.0, 134.4, 131.5, 131.0, 127.7, 124.6, 124.4, 123.0, 120.5, 119.5, 116.9, 113.2, 9.6.; HRMS (ESI) *m*/*z*: [M + H]^+^ Calcd for, C_15_H_13_NO_2_S^79^Br 349.9850; Found 349.9854.

### 1-[(2-Bromo-4,5-dimethoxyphenyl)sulfonyl]-1*H*-indole **14c**

Indole **13a** (132 mg, 1.13 mmol, 1.1 equiv) was dissolved in dry DMF (10 mL) and at room temperature treated with 60% w/w NaH in mineral oil (50 mg, 1.25 mmol, 1.2 equiv). After 0.25 h, 2-bromo-4,5-dimethoxybenzenesulfonyl chloride^1^ (322 mg, 1.02 mmol, 1 equiv) was added. The reaction mixture was stirred for 15 h before EtOAc (15 mL) and H_2_O (25 mL) were added. The resultant aqueous layer was further extracted with EtOAc (3 × 15 mL), and the combined organic extracts were dried of MgSO_4_. Filtration, followed by solvent removal under reduced pressure, afforded the crude sulfonamide, which was purified by flash column chromatography (*c*-Hex-EtOAc; 5:1) which gave **14c** (282 mg, 70%) as a colorless solid. M.P. 126–128 °C. *R*_f_ = 0.15 (*c*-Hex-EtOAc; 5:1). *v̅*_max_ (dep. CH_2_Cl_2_) 3155, 3105, 3081, 2971, 2840, 1580, 1502, 1443, 1371, 1257, 1221, 1167, 1133, 1019 cm^–1^. ^1^H NMR (400 MHz, CDCl_3_): δ 7.74 (d, 1H, *J* = 3.5 Hz), 7.64 (s, 1H), 7.67–7.63 (m, 1H), 7.57–7.53 (m, 1H), 7.23–7.18 (m, 2H), 7.01 (s, 1H), 6.63 (dd, 1H, *J* = 3.5, 0.5 Hz), 3.89 (s, 3H), 3.83 (s, 3H). ^13^C{^1^H} NMR (100 MHz, CDCl_3_): δ 153.2, 147.9, 134.4, 130.6, 129.2, 128.2, 124.2, 123.2, 121.5, 117.8, 113.8, 112.9, 112.8, 107.1, 56.55, 56.5. HRMS (ESI) *m*/*z*: [M + H]^+^, Cacld for C_16_H_15_NO_4_S^79^Br 395.9905; Found 395.9889.

### Benzo[4,5]isothiazolo[2,3-*a*]indole 5,5-dioxide **15a**(22)

Compound **14a** (224 mg, 0.67 mmol, 1 equiv) was dissolved in dry DMF (7 mL), which was degassed under a stream of N_2_ for 15 min. Pd(OAc)_2_ (15 mg, 0.07 mmol, 10 mol %) and PPh_3_ (35 mg, 0.13 mmol, 20 mol %) were added followed by K_2_CO_3_ (185 mg, 1.34 mmol, 2 equiv). The mixture was heated at 110 °C (oil bath temperature) for 45 min. On cooling, extraction was performed using EtOAc (20 mL) and H_2_O (20 mL). The resultant aqueous layer was further extracted with EtOAc (3 × 15 mL), and the combined organic layers were dried over MgSO_4_. Filtration was followed by solvent removal under reduced pressure and then purification by flash column chromatography (*c*-Hex-EtOAc; 2:1), which gave the title compound **15a** (165 mg, 97%) as a colorless crystalline solid. M.P. 212–213 °C. *R*_f_ = 0.25 (*c*-Hex-EtOAc; 1:1). *v̅*_max_ (dep. CH_2_Cl_2_) 3055, 2927, 1438, 1320, 1265, 1181 cm^–1^. ^1^H NMR (400 MHz, CDCl_3_): δ 7.82 (d, 1H, *J* = 7.5 Hz), 7.73–7.70 (m, 2H), 7.64 (dt, 1H, *J* = 7.5, 0.5 Hz), 7.59 (d, 1H, *J* = 7.5 Hz), 7.48 (dt, 1H, *J* = 7.5, 0.5 Hz), 7.36 (dt, 1H, *J* = 7.5, 0.5 Hz), 7.23 (dt, 1H, *J* = 7.5, 0.5 Hz), 6.82 (s, 1H). ^13^C{^1^H} NMR (100 MHz, CDCl_3_) δ 138.3, 134.0, 133.1, 132.9, 132.8, 129.2, 127.6, 125.9, 123.4, 122.55, 122.5, 122.3, 111.8, 101.0. Microanalysis: found C, 65.67; H, 3.29; N, 5.32%; C_14_H_9_NO_2_S requires C, 65.88; H, 3.53; N, 5.49%.

### 11-Methylbenzo[4,5]isothiazolo[2,3-*a*]indole 5,5-dioxide **15b**

Compound **14b** (95 mg, 0.27 mmol, 1 equiv) was dissolved in dry DMF (3 mL), which was degassed under a stream of N_2_ for 0.25 h. PdOAc_2_ (6 mg, 0.027 mmol, 10 mol %), PPh_3_ (14 mg, 0.053 mmol, 20 mol %), and K_2_CO_3_ (75 mg, 0.542 equiv) were added and the mixture heated to 110 °C (oil bath temperature) for 2 h. On cooling, EtOAc (10 mL) and H_2_O (10 mL) were added. The resultant aqueous layer was further extracted with EtOAc (2 × 10 mL), and the combined organic extracts were dried of MgSO_4_. Filtration, followed by solvent removal under reduced pressure, afforded the crude adduct, which was purified by recrystallization (EtOH), which gave **15b** (65 mg, 90%) as a colorless crystalline solid. M.P. 158–160 °C (EtOH). *R*_f_ = 0.2 (*c*-Hex-EtOAc; 4:1). *v̅*_max_ (dep. CH_2_Cl_2_) 3055, 2986, 1603, 1440, 1321, 1265, 1180 cm^–1^. ^1^H NMR (400 MHz, CDCl_3_): δ 7.81 (d, 1H, *J* = 7.5 Hz), 7.78 (d, 1H, *J* = 7.5 Hz), 7.67 (d, 1H, *J* = 7.5 Hz), 7.64 (t, 1H, *J* = 7.5 Hz), 7.53 (d, 1H, *J* = 7.5 Hz), 7.44 (t, 1H, *J* = 7.5 Hz), 7.36 (t, 1H, *J* = 7.5 Hz), 7.25 (t, 1H, *J* = 7.5 Hz), 2.45 (s, 3H). ^13^C{^1^H} NMR (100 MHz, CDCl_3_): δ 138.2, 134.3, 133.9, 132.6, 129.1, 128.5, 128.4, 126.1, 123.0, 122.7, 122.4, 120.5, 113.1, 111.8, 9.3. HRMS (ESI) *m*/*z*: [M + H]^+^ Calcd for C_15_H_12_NO_2_S 270.0589; Found 270.0587.

### 2,3-Dimethoxybenzo[4,5]isothiazolo[2,3-*a*]indole 5,5-dioxide **15c**

A solution of **14c** (269 mg, 0.679 mmol, 1 equiv) in degassed DMF (5 mL) was treated with Pd(OAc)_2_ (3 mg, 0.013 mmol, 2 mol %), PPh_3_ (7 mg, 0.027 mmol, 4 mol %), and K_2_CO_3_ (188 mg, 1.36 mmol, 2 equiv). The mixture was heated at 110 °C (oil bath temperature) under a N_2_ atmosphere for 3 h. On cooling, EtOAc (15 mL) and H_2_O (30 mL) were added. The resultant aqueous layer was further extracted with EtOAc (3 × 10 mL), and the combined organic extracts dried over MgSO_4_. Filtration, followed by solvent removal under reduced pressure, gave the crude product which was further purified by flash column chromatography (*c*-Hex-EtOAc; 3:1 to 1:1) which gave **15c** (191 mg, 89%) as a colorless solid. Recrystallization from EtOAc gave crystals suitable for X-ray diffraction. M.P. 209–211 °C (EtOAc). *R*_f_ = 0.5 (*c*-Hex-EtOAc; 1:1). *v̅*_max_ (solid) 3114, 3087, 3070, 3006, 2928, 2835, 1587, 1494, 1461, 1436, 1417, 1327, 1315, 1293, 1248, 1156, 1140, 1044 cm^–1^. ^1^H NMR (400 MHz, CDCl_3_): δ 7.66 (d, 1H, *J* = 7.5 Hz), 7.52 (d, 1H, *J* = 7.5 Hz), 7.34 (t, 1H, *J* = 7.5 Hz), 7.23 (s, 1H), 7.21 (t, 1H, *J* = 7.5 Hz), 7.05 (s, 1H,), 6.64 (s, 1H), 3.99 (s, 3H), 3.97 (s, 3H). ^13^C{^1^H} NMR (100 MHz, CDCl_3_): δ 153.9, 150.4, 133.3, 133.1, 132.9, 129.9, 125.4, 123.2, 122.3, 121.5, 111.4, 104.1, 103.8, 99.6, 58.5, 58.45. HRMS (ESI) *m*/*z*: [M + H]^+^, Calcd for C_16_H_14_NO_4_S316.0644; Found 316.0633.

### 2-Phenyl-1*H*-indole **16a**(24)

Following the general procedure, Mg ribbon (135 mg, 5.55 mmol, 35 equiv) was added to a solution of sulfonamide **15a** (41 mg, 0.16 mmol, 1 equiv) in MeOH (5 mL). A crystal of I_2_ (ca. 10 mg) was added, and the mixture was stirred at rt for 15 h. EtOAc (20 mL) and sat. NH_4_Cl solution (25 mL) were added. The resultant aqueous layer was further extracted with EtOAc (3 × 15 mL), and the combined organic layers dried over MgSO_4_. Filtration, followed by solvent removal and flash column chromatography (*c*-Hex-EtOAc; 19:1 to 9:1), gave **16a** (25 mg, 81%) as a colorless solid. M.P. 150–152 °C. *R*_f_ = 0.2 (*c*-Hex-EtOAc; 9:1). ^1^H NMR (400 MHz, CDCl_3_): δ 8.38–8.30 (s(br, 1H)), 7.67 (d, 2H, *J* = 7.0 Hz), 7.64 (d, 1H, *J* = 8.0 Hz), 7.45 (t, 2H, *J* = 7.0 Hz), 7.42 (d, 1H, *J* = 8.0 Hz), 7.33 (t, 1H, *J* = 7.0 Hz), 7.22–7.18 (m, 1H, m), 6.85–6.83 (m, 1H, m), 7.15–7.11 (m, 1H). ^13^C{^1^H} NMR (100 MHz, CDCl_3_): δ 137.9, 136.8, 132.4, 129.3, 129.0, 127.7, 125.2, 122.4, 120.7, 120.3, 110.9, 100.0.

### 3-Methyl-2-phenyl-1*H*-indole **16b**(24)

A solution of **15b** (41 mg, 0.152 mmol, 1 equiv) in MeOH (4 mL) was treated with Mg ribbon (133 mg, 5.473 mmol, 35 equiv) and a crystal of I_2_. Stirring was continued for 5 h whereupon EtOAc (20 mL) and sat. NH_4_Cl solution (25 mL) were added. The resultant aqueous layer was further extracted with EtOAc (3 × 15 mL), and the combined organic layers dried over MgSO_4_. Filtration, followed by solvent removal and flash column chromatography (*c*-Hex-EtOAc; 19:1 to 9:1), gave **16b** (22 mg, 69%) as a colorless solid. M.P. 95–100 °C (decomp). *R*_f_ = 0.2 (*c*-Hex-EtOAc; 9:1). *v̅*_max_ 3188, 3062, 3027, 2960, 2923, 2853, 1673, 1645, 1608, 1585, 1529, 1494, 1443, 1360, 1314, 1299, 1259, 1246, 1199, 1184, 1143, 1093, 1053, 1028, 764, 697, 680, 609, 580 cm^–1^. ^1^H NMR (400 MHz, CDCl_3_): δ 7.81 (s(br), 1H), 7.61–7.59 (m, 1H), 7.58–7.56 (m, 2H), 7.48–7.45 (m, 2H), 7.37–7.33 (m, 2H), 7.24–7.19 (m, 1H), 7.16–7.13 (m, 1H), 2.46 (s, 3H). ^13^C{^1^H} NMR (100 MHz, CDCl_3_): δ 136.0, 134.2, 133.5, 130.2, 129.0, 128.0, 127.5, 122.5, 120.0, 119.1, 111.0, 109.0, 9.8.

### 2-(3,4-Dimethoxyphenyl)-1*H*-indole **16c**(25)

Compound **15c** (50 mg, 0.15 mmol, 1 equiv) was dissolved in MeOH (5 mL) and THF (4 mL) in a sealed tube with a circular stirrer bar. To this reaction mixture, Mg powder (0.13 g, 5.54 mmol, 35 equiv) was added along with a crystal of iodine. This reaction mixture was sealed and left stirring at rt for 2.5 h. The reaction was quenched with sat. NH_4_Cl solution (10 mL). EtOAc (20 mL) was added and the organic layer was removed. The aqueous layer was further extracted with EtOAc (2 × 15 mL), and the combined organic layers were dried over MgSO_4_. After filtration and solvent removal under reduced pressure. *R*_f_ = 0.35 (*c*-Hex-EtOAc; 3:1) afforded the product **16c** (31 mg, 81%), as a yellow solid. *R*_f_ = 0.4 (*c*-Hex-EtOAc; 2:1). *v̅*_max_ 3366, 3055, 3001, 2956, 2928, 2838, 1695, 1607, 1587, 1504, 1455, 1303, 1256, 1164, 1141, 1023, 854, 768, 749 cm^–1^. ^1^H NMR (400 MHz, CDCl_3_): δ 8.36 (s(br), 1H) 7.38 (d, 1H, *J* = 8.0 Hz), 7.21–7.18 (m, 3H), 7.16–7.10 (m, 1H), 7.61 (d, 1H, *J* = 8.0 Hz), 6.93 (d, 1H, *J* = 8.5 Hz), 6.73 (d, 1H, *J* = 1.5 Hz), 3.97 (s, 3H), 3.92 (s, 3H). ^13^C{^1^H} NMR (100 MHz, CDCl_3_): δ 149.4, 149.0, 138.1, 136.7, 129.4, 125.5, 122.0, 120.4, 120.2, 111.6, 111.5, 110.7, 108.9, 99.2, 56.0.

### 3-(2-Deuteriophenyl)-1-tosylpyrrolidine d-**8a**

Under N_2,_ Mg powder (203 mg, 8.35 mmol, 35 equiv) and a crystal of iodine^2,3^ were added to a solution of **7a** (50 mg, 0.239 mmol, 1 equiv) in CD_3_OD (5 mL). The mixture was heated and stirred at 50 °C for 15 h. The resulting suspension was cooled, and solid NH_4_Cl (ca. 1.0 g) was added and diluted with CH_2_Cl_2_ (15 mL). A solution of 1 M NaOH (10 mL) was added (until the pH was 12) and stirred for 20 min. The resultant aqueous layer was extracted with CH_2_Cl_2_ (4 × 25 mL). The combined organic layers were dried over anhydrous MgSO_4_. Filtration followed by solvent removal under reduced pressure afforded the crude amine, which was taken up in CH_2_Cl_2_ (10 mL). To this solution, Et_3_N (0.07 mL, 0.65 mmol, 2 equiv) and TsCl (46 mg, 0.239 mmol, 1 equiv) were added at 0 °C. Stirring was continued for 15 h and the reaction gradually warmed to room temperature. Silica (ca. 2.0 g) was added to the reaction mixture and the solvent was removed under pressure. Purification by flash chromatography (*c*-Hex-EtOAc; 3:1) gave d-**8a** (48 mg, 67%) as a colorless solid. M.P. 58–60 °C. *R*_f_ = 0.3 (*c*-Hex-EtOAc; 3:1).*v̅*_max_ 2976, 2923, 2843, 1596, 1475, 1335, 1156, 1032, 956, 815, 776, 661, 631, 588, 548 cm^–1^. ^1^H NMR (400 MHz, CDCl_3_): δ 7.77 (d, 2H, *J* = 8.0 Hz), 7.35 (d, 2H, *J* = 8.0 Hz), 7.30–7.18 (m, 3H), 7.10 (d, 1H, *J* = 7.0 Hz), 3.78–3.70 (m, 1H), 3.54 (ddd, 1H, *J* = 10.0, 8.5, 3.5 Hz), 3.37 (m, 1H), 3.29–3.18 (m, 2H), 2.46 (s, 3H), 2.25–2.17 (m, 1H), 1.94–1.82 (m, 1H). ^13^C{^1^H} NMR (100 MHz, CDCl_3_): δ 143.4, 140.5, 133.9, 129.6, 128.5, 128.4, 127.5, 126.9, 126.85, 126.6 (t, *J* = 24.0 Hz), 54.0, 47.7, 43.7, 32.8, 21.4. HRMS (ESI) *m*/*z*: [M + Na]^+^ Calcd for C_17_H_18_DNO_2_SNa 325.0989; Found 325.0987.

### 2-(2-Deuteriophenyl)-1*H*-pyrrole d-**12a**

Under N_2_, Mg turnings (242 mg, 10.0 mmol, 35 equiv) were added to a solution of sulfonamide **11a** (59 mg, 0.286 mmol, 1 equiv) in CD_3_OD (5 mL). A crystal of I_2_ (∼10 mg) was added, and the mixture was stirred at 50 °C (oil bath temperature) for 2.5 h in a sealed tube. EtOAc (20 mL) and sat. NH_4_Cl solution (25 mL) were added. The resultant aqueous layer was further extracted with EtOAc (3 × 15 mL), and the combined organic layers were dried over MgSO_4_. Following filtration and solvent removal under reduced pressure, the crude product was purified by column chromatography (*c*-Hex-EtOAc; 9:1) affording d-**12a** (17 mg, 41%) as a pale pink solid. M.P. 109–111 °C. *R*_f_ = 0.55 (*c*-Hex-EtOAc; 4:1). *v̅*_max_ 3430, 3380, 3057, 2959, 2923, 2854, 1549, 1476, 1460, 1452, 1107, 1029, 917 879, 83, 770, 751, 716, 617, 528 cm^–1^. ^1^H NMR (400 MHz, CDCl_3_): δ 8.41 (s(br), 1H), 7.46 (dd, 1H, *J* = 8.0, 1.0 Hz), 7.37–7.34 (m, 2H), 7.20 (dt, 1H, *J* = 7.0, 1.0 Hz), 6.86–6.84 (m, 1H), 6.53–6.52 (m, 1H), 6.31–6.29 (m, 1H). ^13^C{^1^H} NMR (100 MHz, CDCl_3_): δ 132.8, 132.2, 129.0, 128.9, 126.3, 124.0 (t, *J* = 24.0 Hz), 119.0, 110.3, 106.1. HRMS (ESI) *m*/*z*: [M + H]^+^ (ESI) Calcd forC_10_H_9_DN 145.0871; Found 145.0871.

### 2-(2-Deutertiophenyl)-1*H*-indole d-**16a**

As described above, Mg powder (155 mg, 6.4 mmol, 35 equiv) was added to a solution of sulfonamide **15a** (47 mg, 0.18 mmol, 1 equiv) in CD_3_OD (3 mL) and THF (2 mL). A crystal of I_2_ (∼10 mg) was added, and the mixture was stirred at rt for 2.5 h. EtOAc (20 mL) and sat. NH_4_Cl solution (25 mL) were added. The resultant aqueous layer was further extracted with EtOAc (3 × 15 mL), and the combined organic layers were washed with (brine + H_2_O) (10 mL) and then dried over MgSO_4_. Filtration, followed by solvent removal and flash column chromatography (*c*-Hex-EtOAc; 2:1), gave d-**16a** (25 mg, 70%) as a colorless solid. *R*_f_ = 0.4 (*c*-Hex-EtOAc 5:1). ^1^H NMR (400 MHz, CDCl_3_): δ 8.36–8.30 (s(br), 1H), 7.67 (d, 1H, *J* = 7.5 Hz), 7.63 (d, 1H, *J* = 7.5 Hz), 7.46–7.43 (m, 2H), 7.40 (d, 1H, *J* = 7.5 Hz), 7.33 (t, 1H, *J* = 7.5 Hz), 7.19 (t, 1H, *J* = 7.5 Hz), 7.12 (t, 1H, *J* = 7.5 Hz), 6.83 (s(br), 1H). ^13^C{^1^H} NMR (100 MHz, CDCl_3_): δ 137.8, 136.8, 132.3, 129.2, 129.0, 128.9, 127.7, 125.1, 124.8 (t, *J* = 25.0 Hz), 122.3, 120.6, 120.2, 110.8, 99.9. HRMS (ESI) *m*/*z*: [M + H]^+^ Calcd for C_14_H_11_DN 195.1035; Found 195.1033.

### 2-(2-Deutertio-4,5-dimethoxyphenyl)-1*H*-indole d-**16c**

Under N_2_, Mg powder (160 mg, 6.55 mmol, 35 equiv) was added to a solution of sulfonamide **15c** (50 mg, 0.16 mmol, 1 equiv) in a mixture of CD_3_OD (5 mL) and THF (4 mL). A crystal of I_2_ (∼10 mg) was added, and the mixture was stirred at rt for 2.5 h. EtOAc (20 mL) and sat. NH_4_Cl solution (25 mL) were added. The resultant aqueous layer was further extracted with EtOAc (3 × 15 mL). The combined organic layers were washed with a saturated solution of brine (10 mL) and then dried over MgSO_4_. Filtration, followed by solvent removal and flash column chromatography (*c*-Hex-EtOAc; 2:1), gave d-**16c** (25 mg, 63%) as a yellow solid. *R*_f_ = 0.4 (*c*-Hex-EtOAc; 2:1). *v̅*_max_ 3360, 3076, 2955, 2924, 2853, 1689, 1605, 1503, 1461, 1439, 1263, 1213, 1173, 1024, 877, 739 cm^–1^. ^1^H NMR (400 MHz, CDCl_3_): δ 8.28–8.21 (s(br), 1H), 7.61–7.57 (m, 1H), 7.38–7.35 (m, 1H), 7.18–7.14 (m, 2H), 7.12–7.08 (m, 1H), 6.93 (s, 1H), 6.72–6.70 (m, 1H), 3.96 (s, 3H), 3.91 (s, 3H). ^13^C{^1^H} NMR (100 MHz, CDCl_3_): δ 149.4, 149.0, 138.1, 136.7, 129.4, 125.5, 122.0, 120.4, 120.2, 117.3 (t, *J* = 25.0 Hz), 111.5, 110.7, 108.9, 99.1, 56.0. HRMS (ESI) *m*/*z*: [M + H]^+^ Calcd for C_16_H_15_DNO_2_ 255.1244; Found 255.1246.

### 2-[3,4^′^-Dimethoxy-(1,1^′^-biphenyl)-2-yl]-*N*-methylethanamine **18**

Using a low-valent titanium reduction, a mixture of **17**(15) (64 mg, 0.18 mmol, 1 equiv) and Mg powder (31 mg, 1.28 mmol, 6 equiv) in THF (3 mL) was added to Ti(O*i*Pr)_4_ (0.06 mL, 0.203 mmol, 1.1 equiv) and Me_3_SiCl (0.05 mL, 0.370 mmol, 2 equiv). The resulting mixture was stirred at 80 °C for 15 h. Aqueous 1 M NaOH (0.4 mL), EtOAc (15 mL), anhydrous NaF (1.0 g), and Celite (1.0 g) were added at room temperature. After stirring for 30 min, the mixture was filtered through a pad of Celite. To the resulting filtrate was added aqueous 1 M NaOH (15 mL), and the mixture was extracted with EtOAc (15 mL). The organic layer was dried over anhydrous MgSO_4_. Filtration followed by solvent removal under reduced pressure afforded **18** (45 mg, 93%) as a viscous oil. *v̅*_max_ 3669, 2956, 2922, 2852, 1729, 1463, 1406, 1378, 1260, 1075, 1026, 862, 801, 754, 720 cm^–1^. ^1^H NMR (400 MHz, CDCl_3_): δ 7.33–7.20 (m, 4H, m), 6.93–6.83 (m, 3H), 3.91 (s, 3H), 3.86 (s, 3H), 3.00–2.91 (m, 2H), 2.67–2.59 (m, 2H), 2.32 (s, 3H). ^13^C{^1^H} NMR (100 MHz, CDCl_3_): δ 148.9, 148.4, 142.2, 137.8, 134.8, 130.7, 129.9, 127.8, 126.6, 121.7, 113.0, 111.3, 53.1, 55.9, 36.4, 33.6. HRMS (ESI) *m*/*z*: [M + H]^+^ (ESI) Calcd for C_17_H_22_NO_2_ 272.1651; Found 272.1657.

### Computational Details

The systems under study have been optimized using the M06-2X,^26^ CAM-B3LYP,^27^ and WB97XD,^28^ functionals and the def2-TZVPD basis set.^29^ Open shell systems have been optimized using UM06-2X, UCAM-B3LYP, and UWB97XD. Frequency calculations have been performed to verify that the geometries obtained correspond to energetic minima. These calculations have been carried out with the Gaussian-16 program.^30^

## Supporting Information Available

Copies of ^1^H and ^13^C{^1^H} NMR spectra; X-ray crystallography for compounds **15c** and **17**; and additional computational details (PDF)
