# Supplementary material for: Reduction of Substituted Benzo-Fused Cyclic Sulfonamides with Mg-MeOH: An Experimental and Computational Study
Source: J Org Chem. 2022 Sep 1;87(18):12087–95. doi: 10.1021/acs.joc.2c01169 (PMC9486945; doi:10.1021/acs.joc.2c01169)
Supplement: Supplementary file 1 — jo2c01169_si_001.pdf [file jo2c01169_si_001.pdf]

Reduction of Substituted Benzo-fused Cyclic Sulfonamides with Mg-MeOH: An Experimental and Computational Study

Aisha Khalifa, Robert Redmond, Goar Sánchez-Sanz and Paul Evans*

Centre for Synthesis and Chemical Biology, School of Chemistry,

University College Dublin, Dublin D04, N2E5, Ireland

Contents

^{1}H and $^{13}C\{^{1}H\}$-NMR Spectra ………………………………………………………………………………………….. S2-S20

Additional Computational Details ……………………………………………………………………………… S21-S32

X-Ray Crystallographic Details for Compounds **15c** and **17** ……………………………………………… S33-S47

^{1}H and $^{13}C\{^{1}H\}$-NMR Spectra

^{1}H-NMR ($CDCl_3$, 400 MHz) spectrum of **8a**

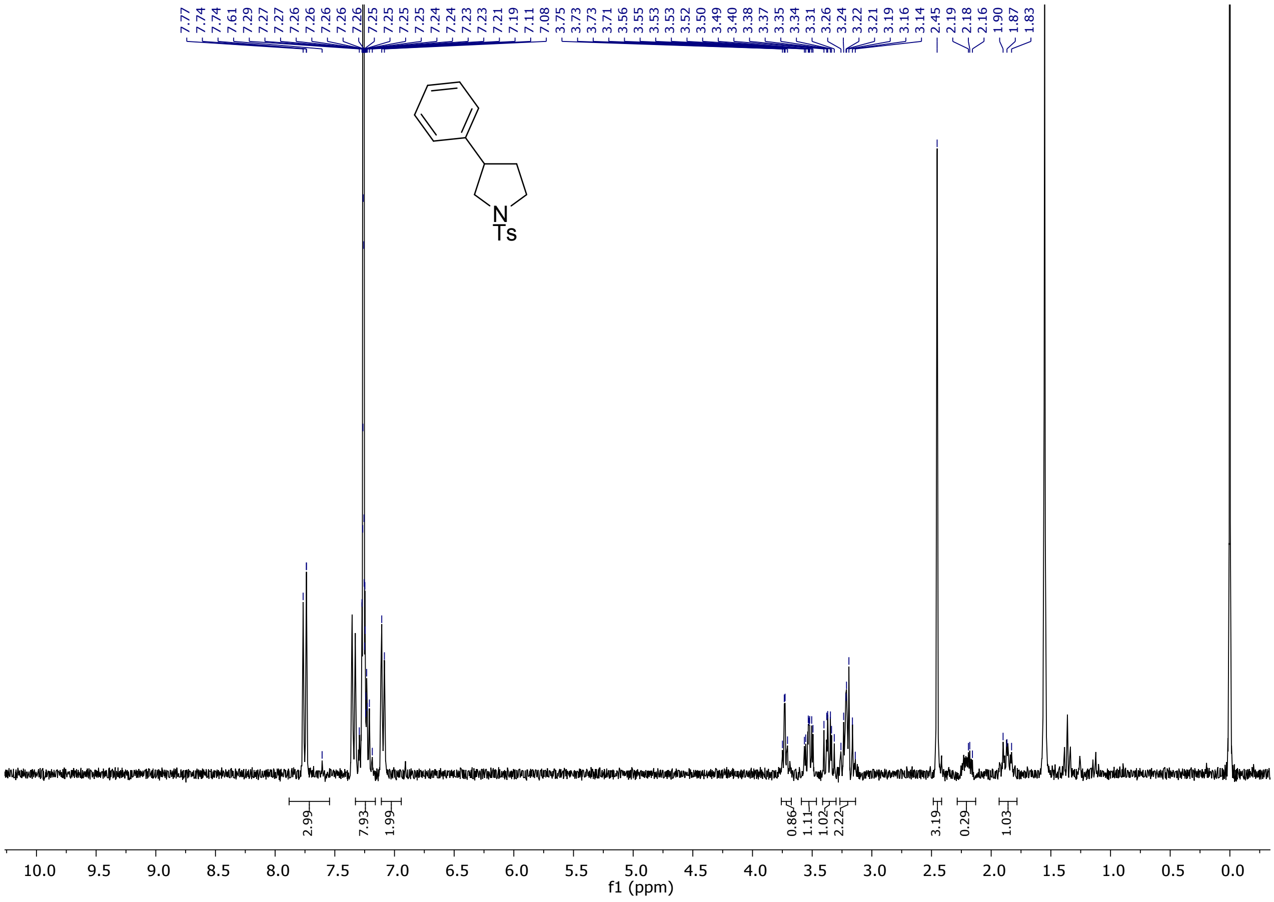

^{1}H-NMR ($CDCl_3$, 400 MHz) spectrum of **8d**

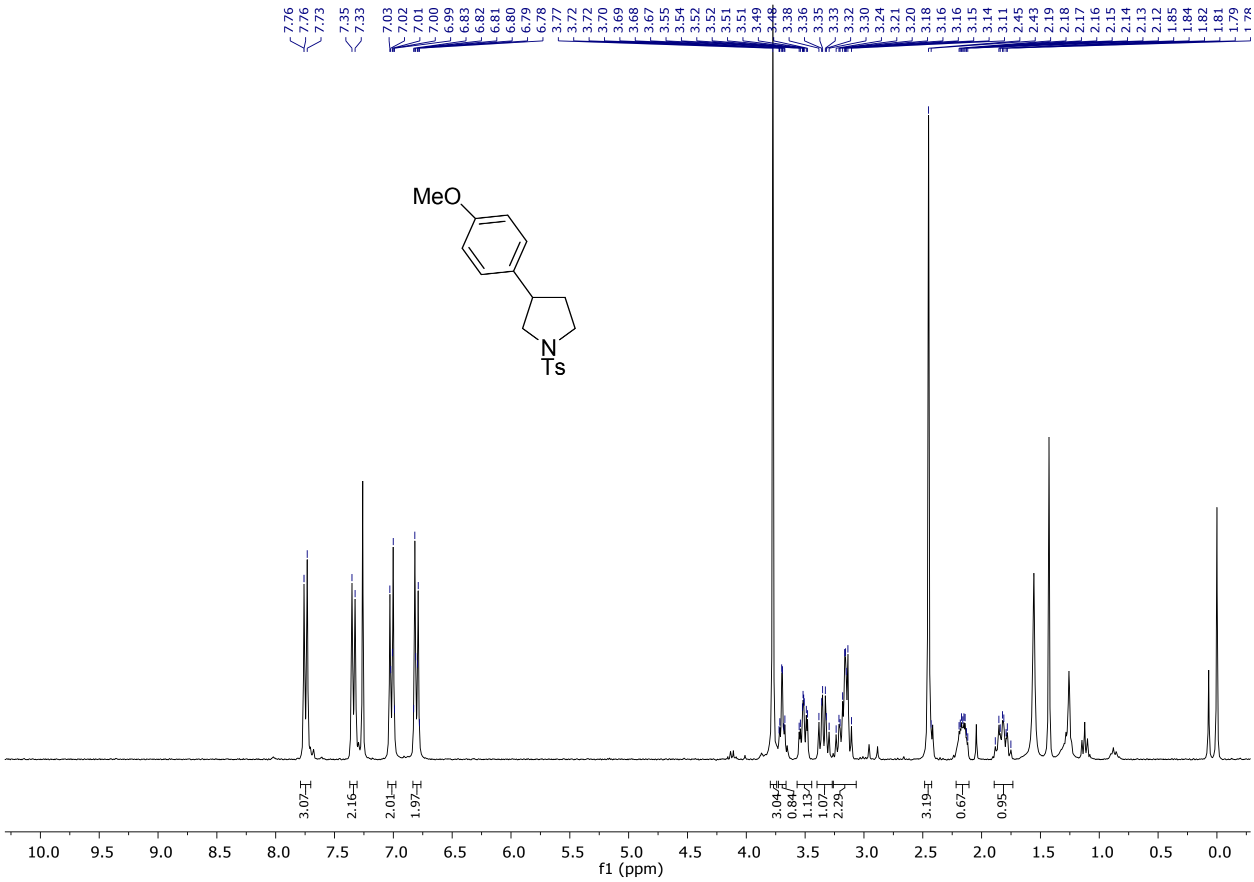

^{1}H-NMR ($CDCl_3$, 400 MHz) spectrum of **10**

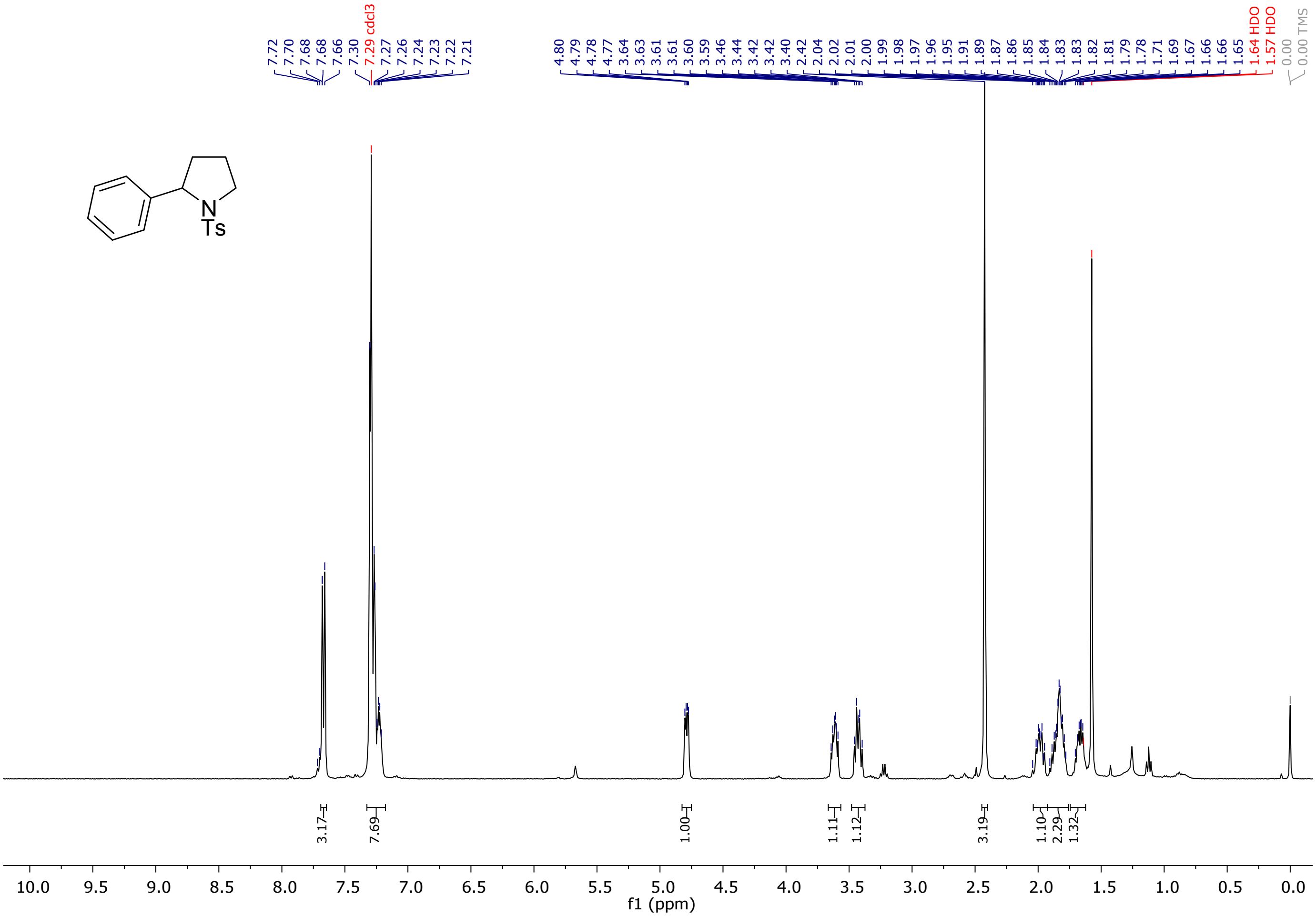

^{1}H-NMR (400 MHz, $CDCl_3$) and $^{13}C\{^{1}H\}$-NMR (100 MHz, $CDCl_3$) spectra for **12a**

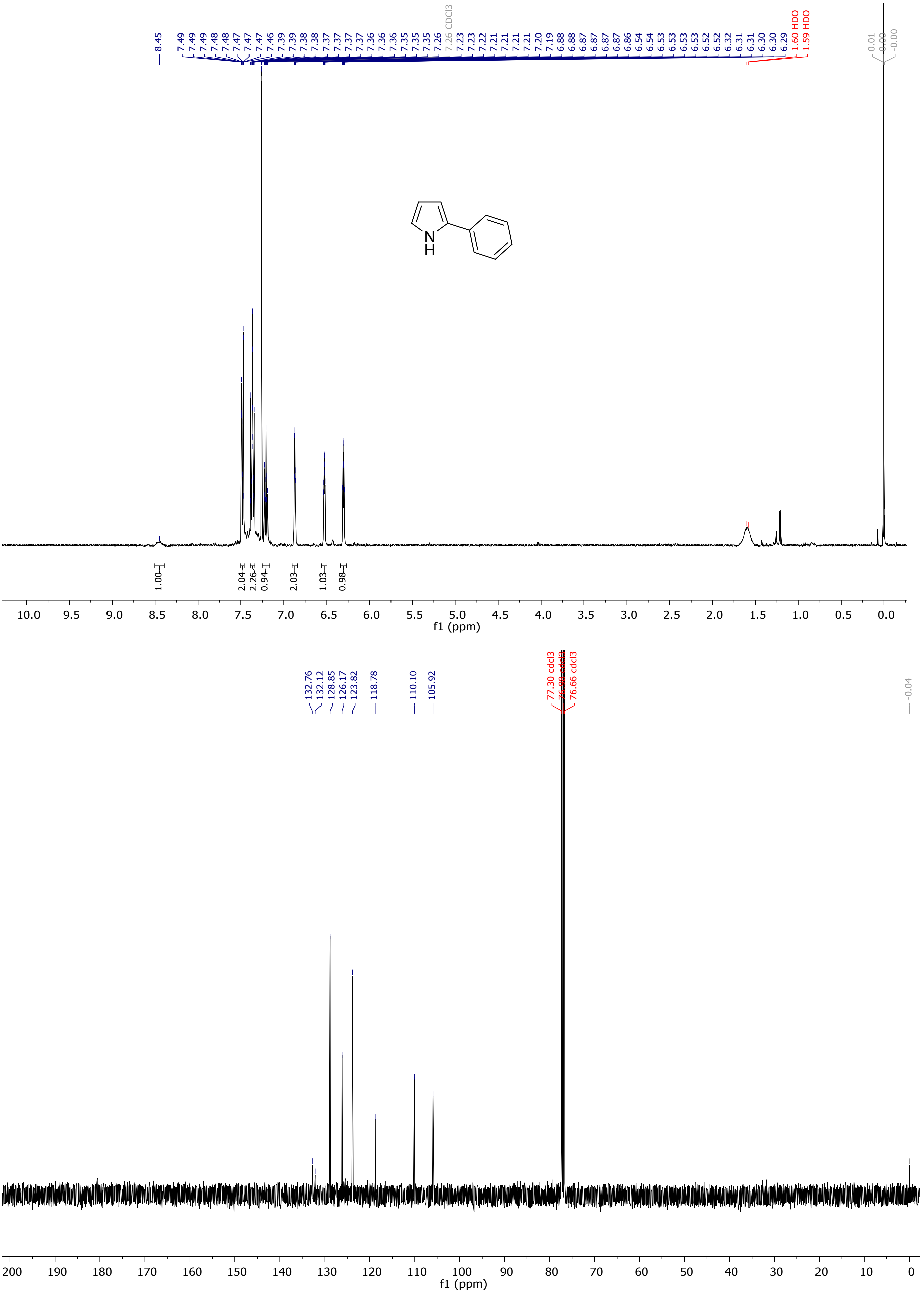

^{1}H-NMR (400 MHz, $CDCl_3$) and $^{13}C\{^{1}H\}$-NMR (100 MHz, $CDCl_3$) spectra for **12b**

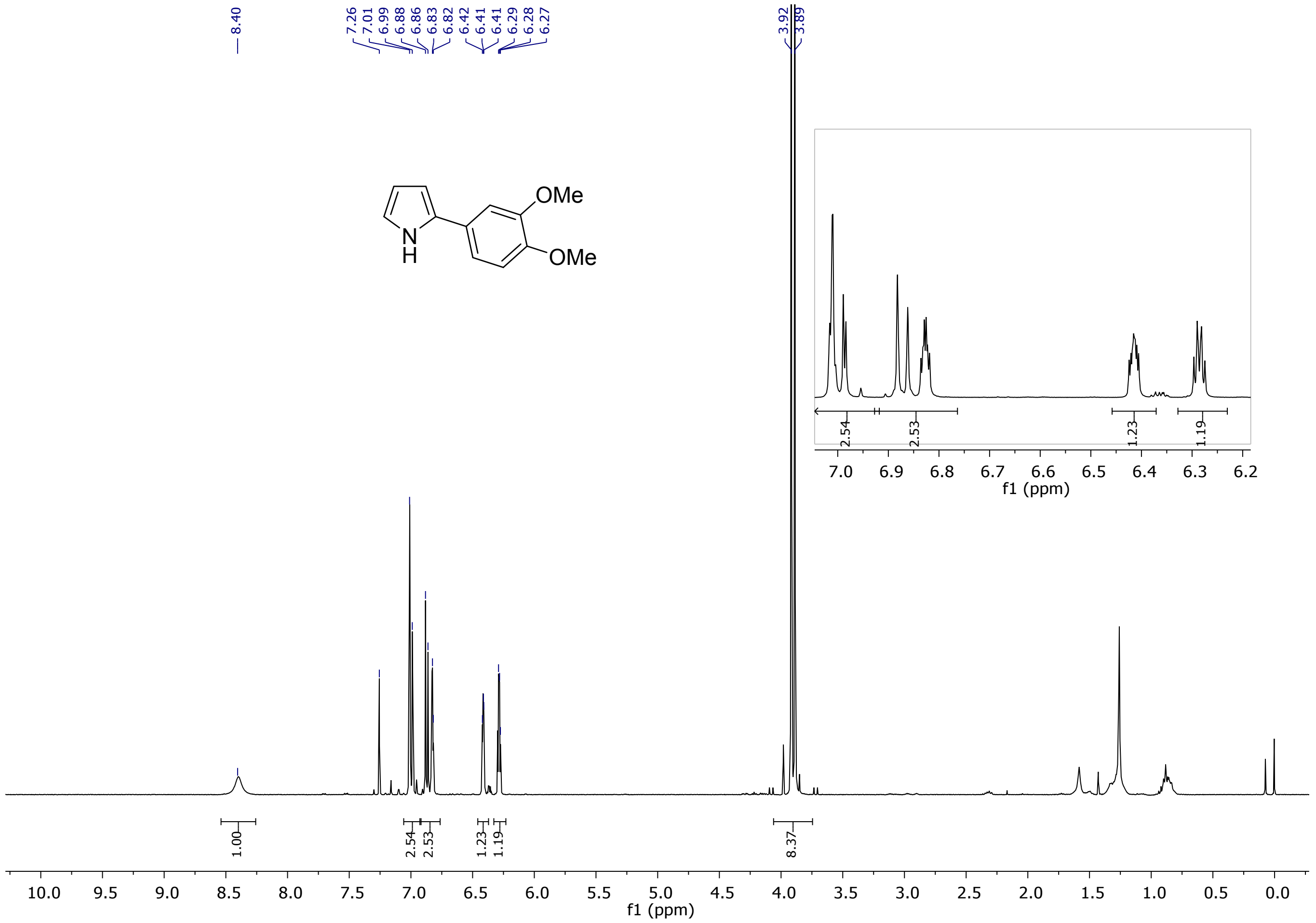

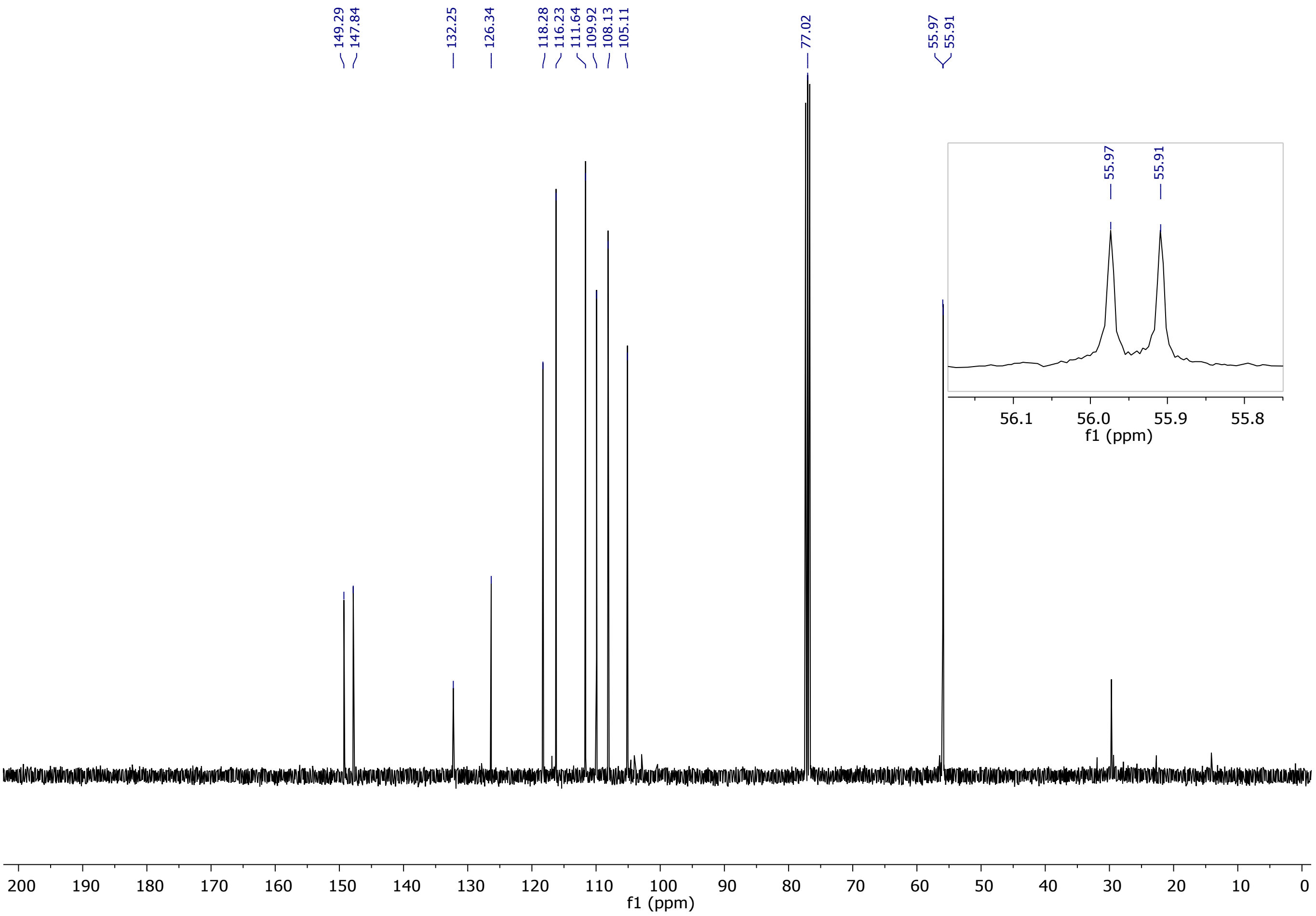

^{1}H-NMR (400 MHz, $CDCl_3$) and ^{13}C{^{1}H}-NMR (100 MHz, $CDCl_3$) spectra for **14a**

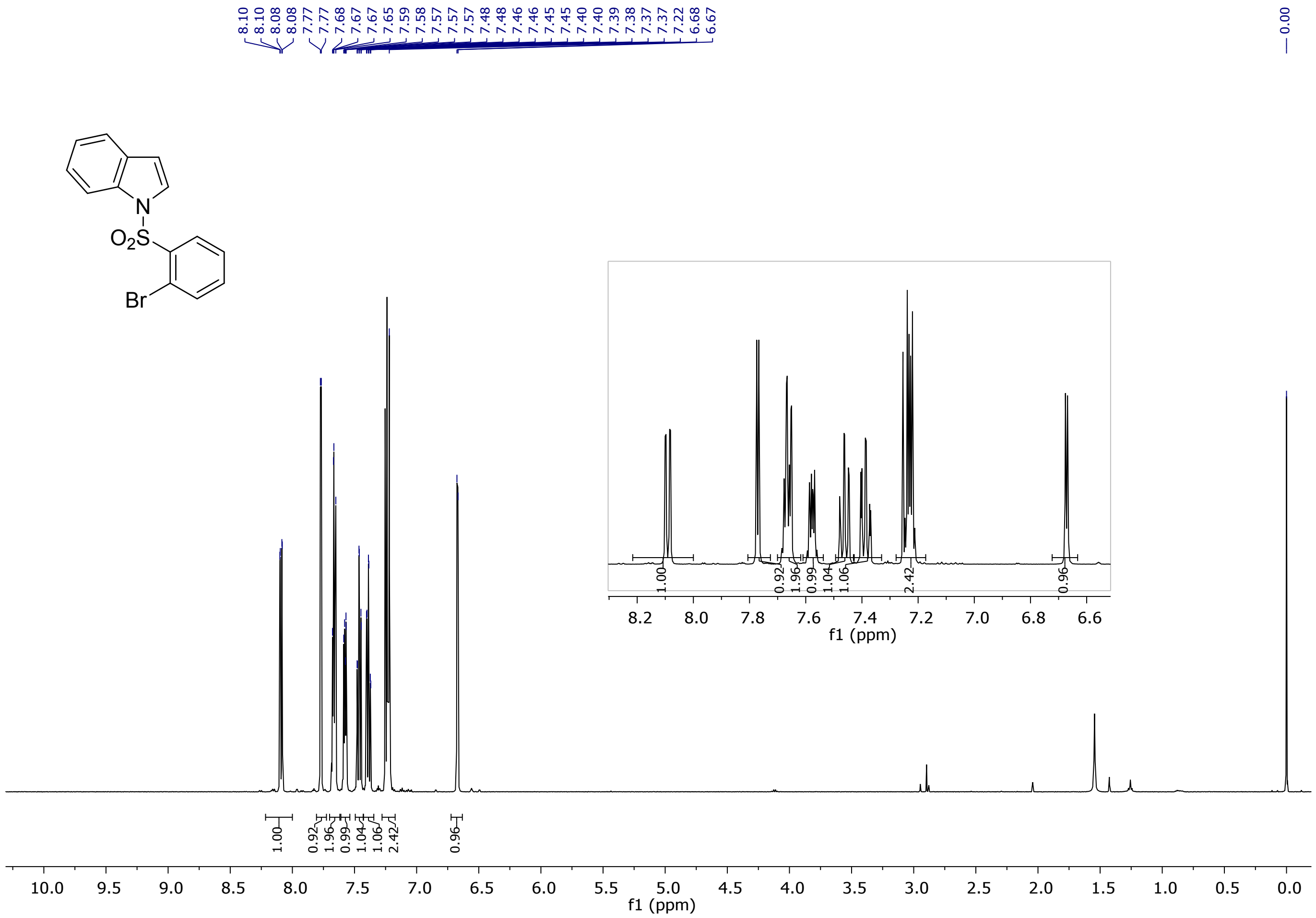

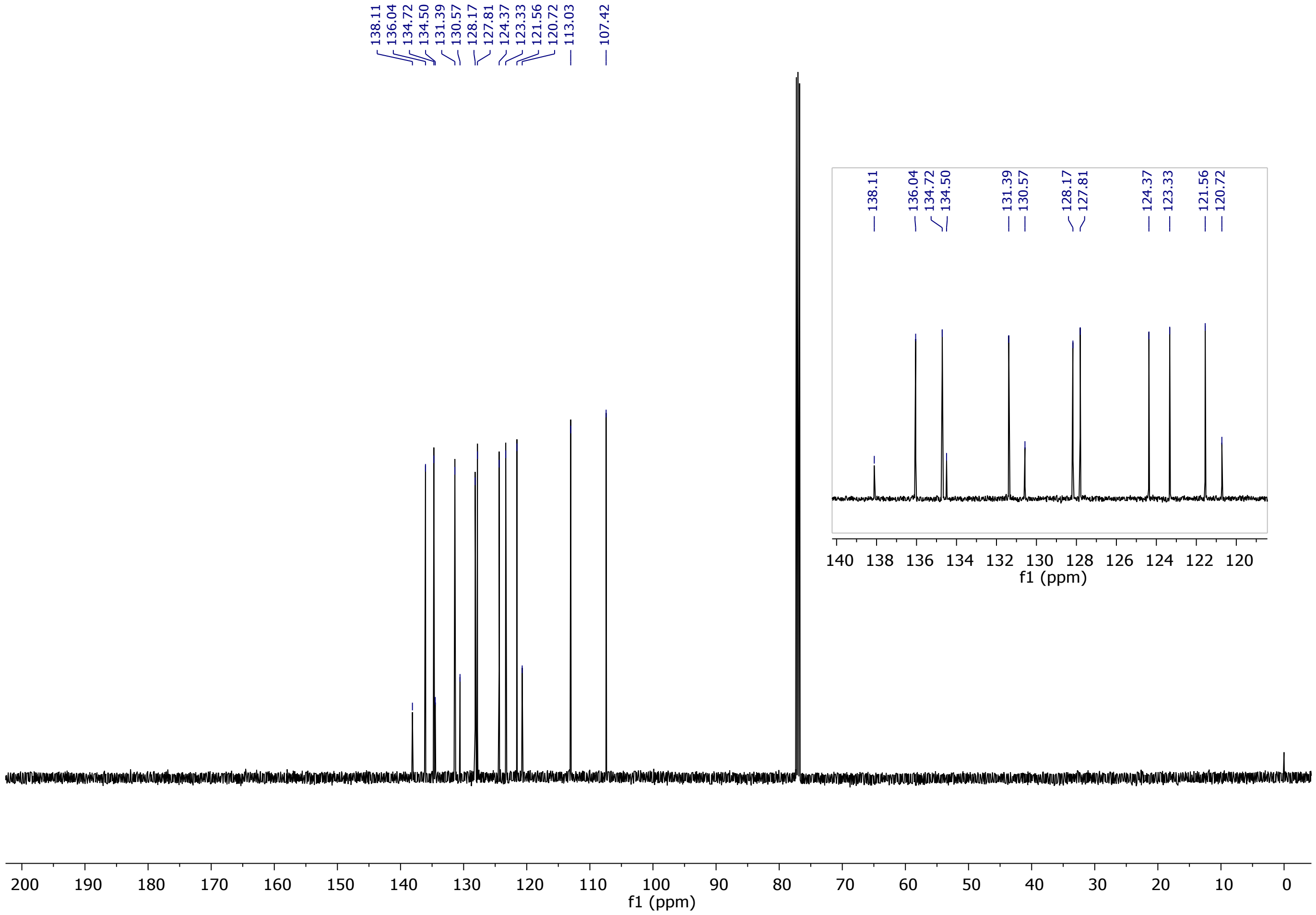

^{1}H-NMR (400 MHz, $CDCl_3$) and $^{13}C\{^{1}H\}$-NMR (100 MHz, $CDCl_3$) spectra for **14b**

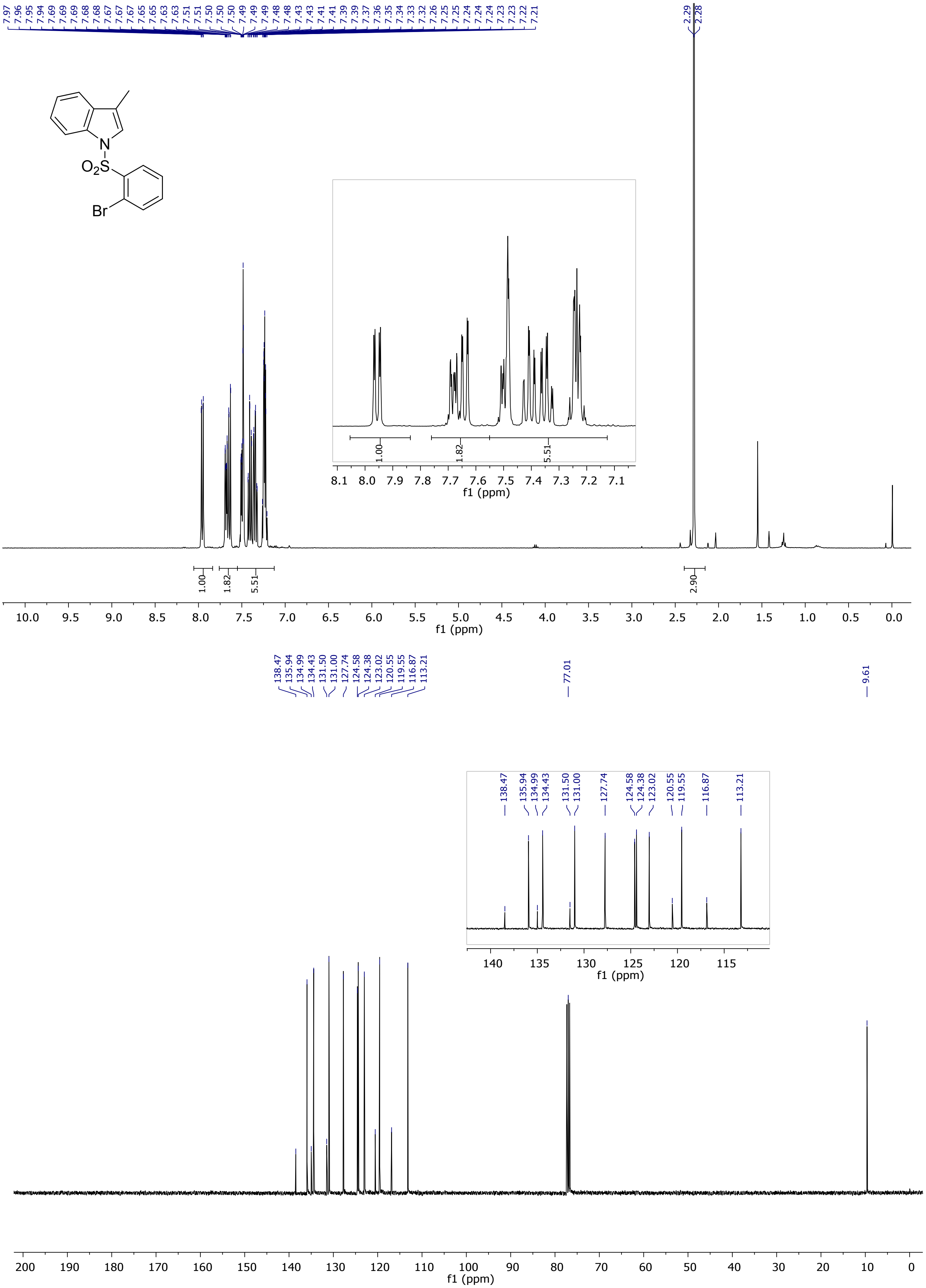

^{1}H-NMR (400 MHz, $CDCl_3$) and ^{13}C{^{1}H}-NMR (100 MHz, $CDCl_3$) spectra for **14c**

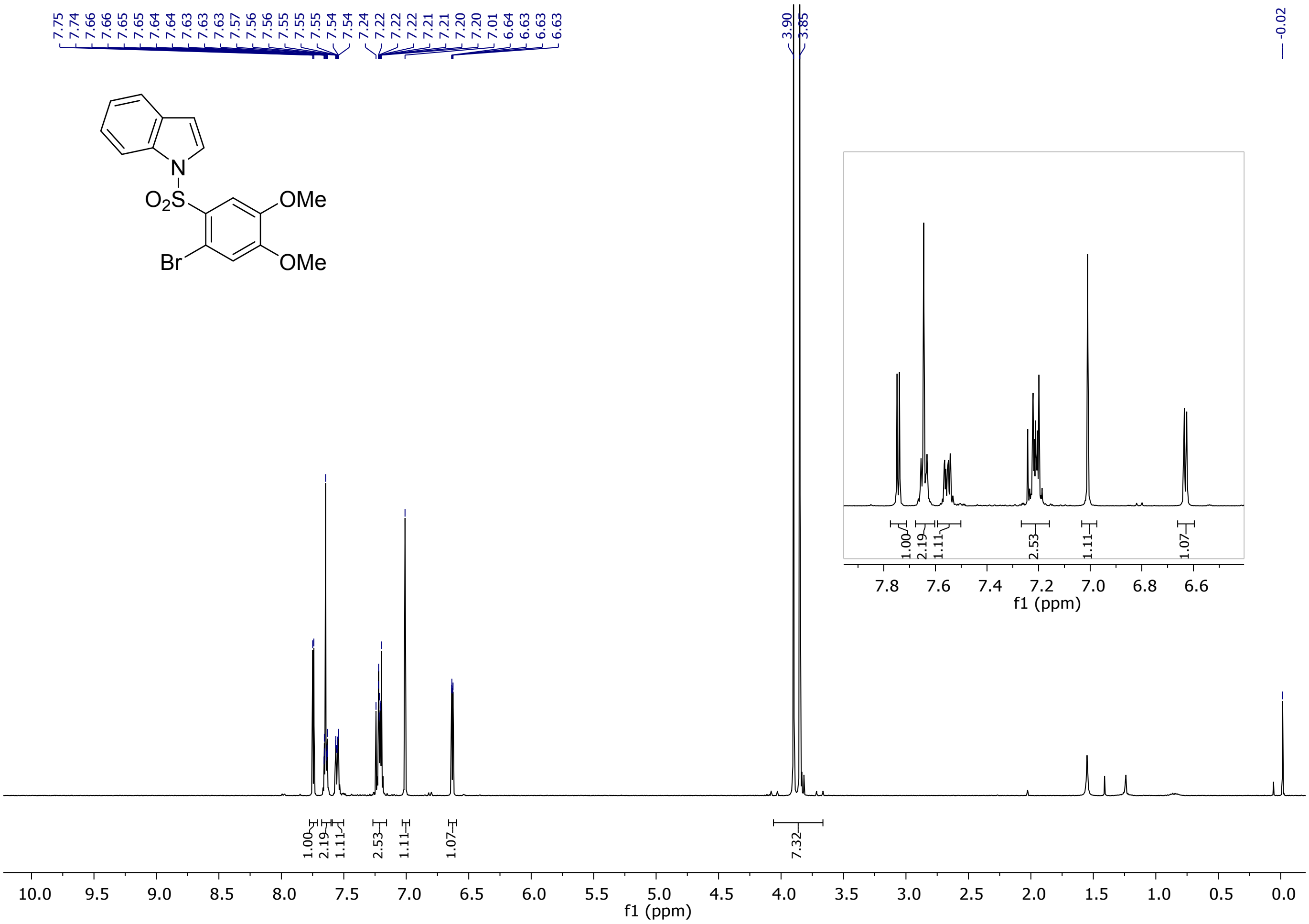

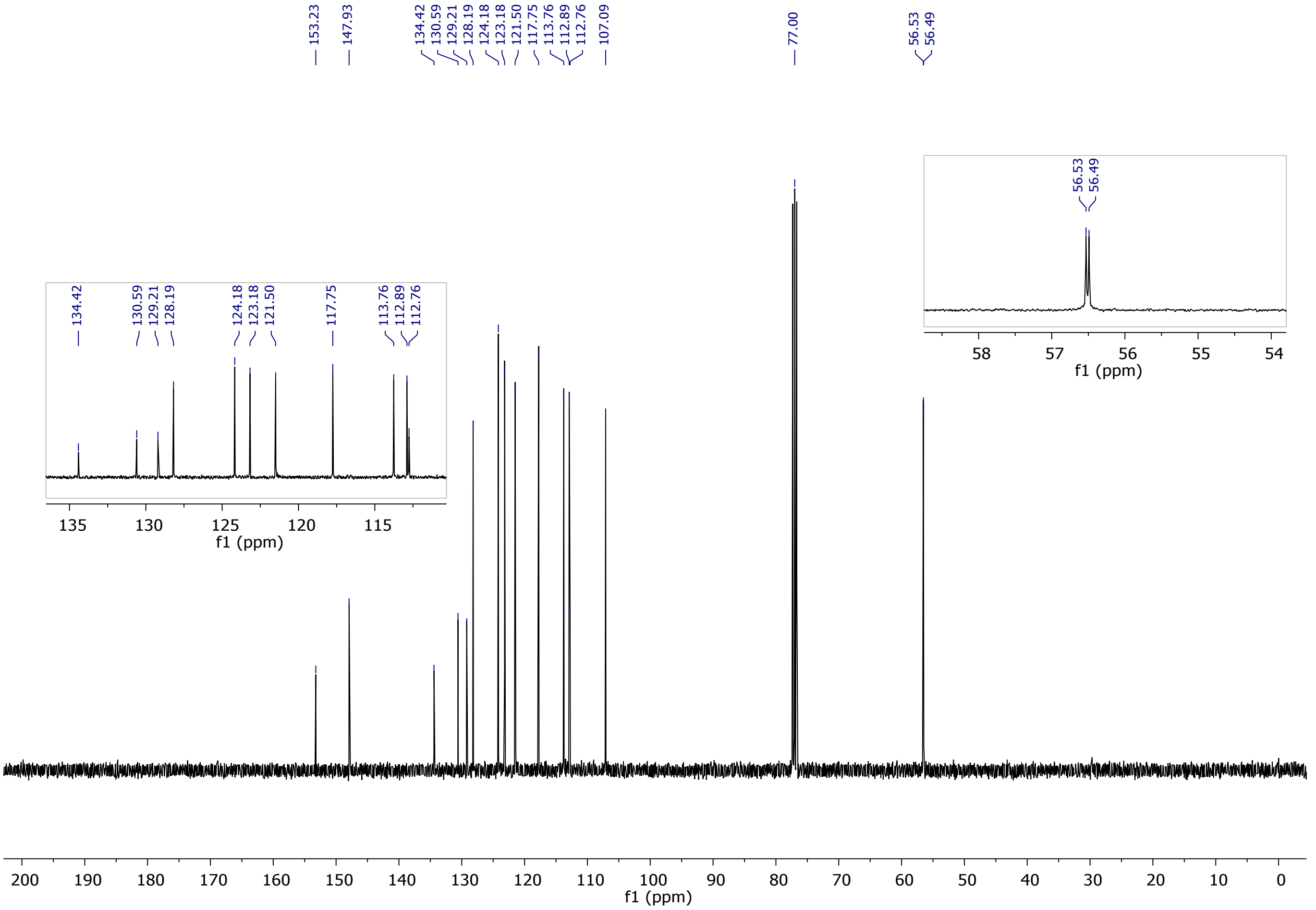

^{1}H-NMR (400 MHz, $CDCl_3$) and $^{13}C\{^{1}H\}$-NMR (100 MHz, $CDCl_3$) spectra for **15a**

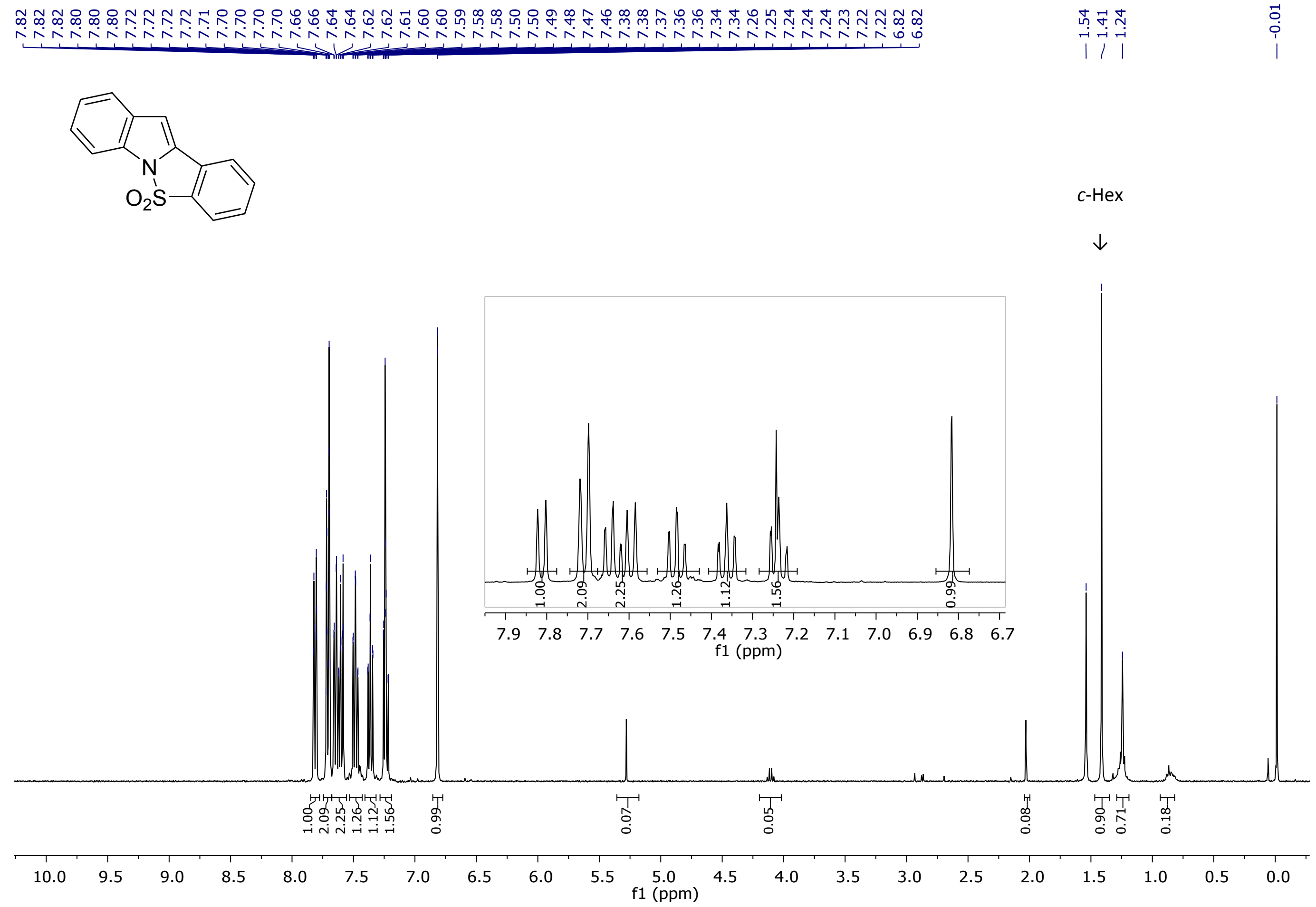

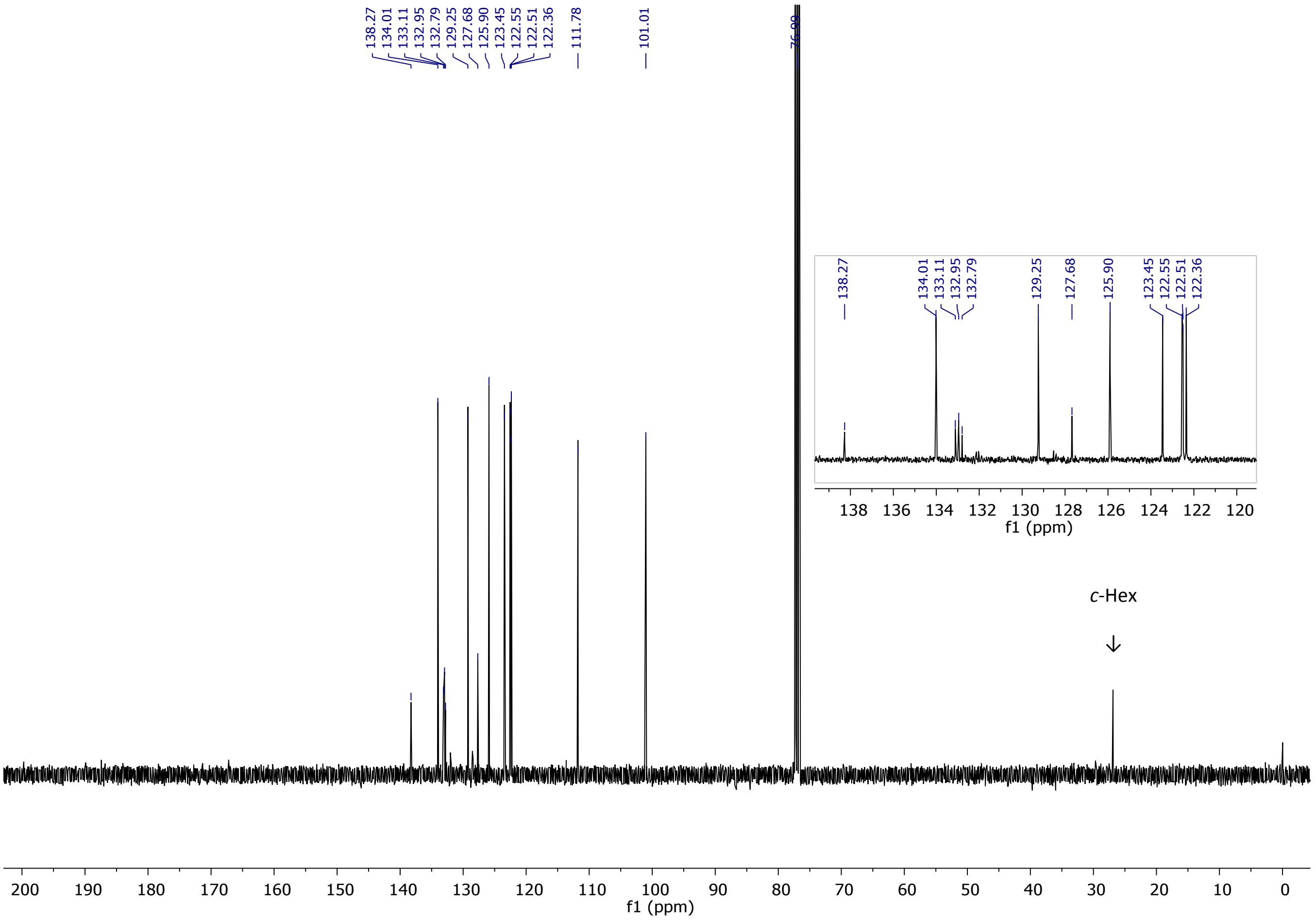

^{1}H-NMR (400 MHz, $CDCl_3$) and $^{13}C\{^{1}H\}$-NMR (100 MHz, $CDCl_3$) spectra for **15b**

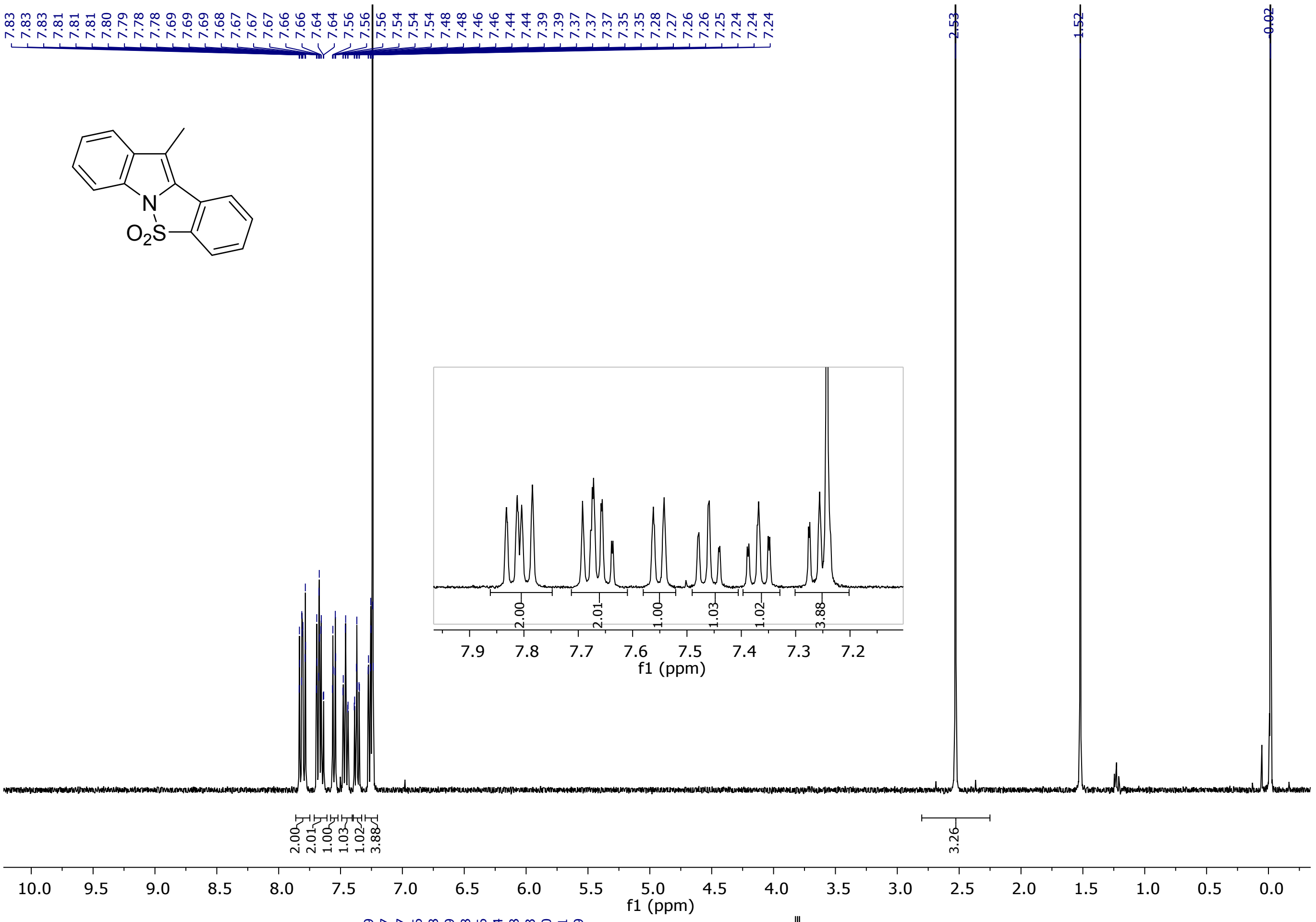

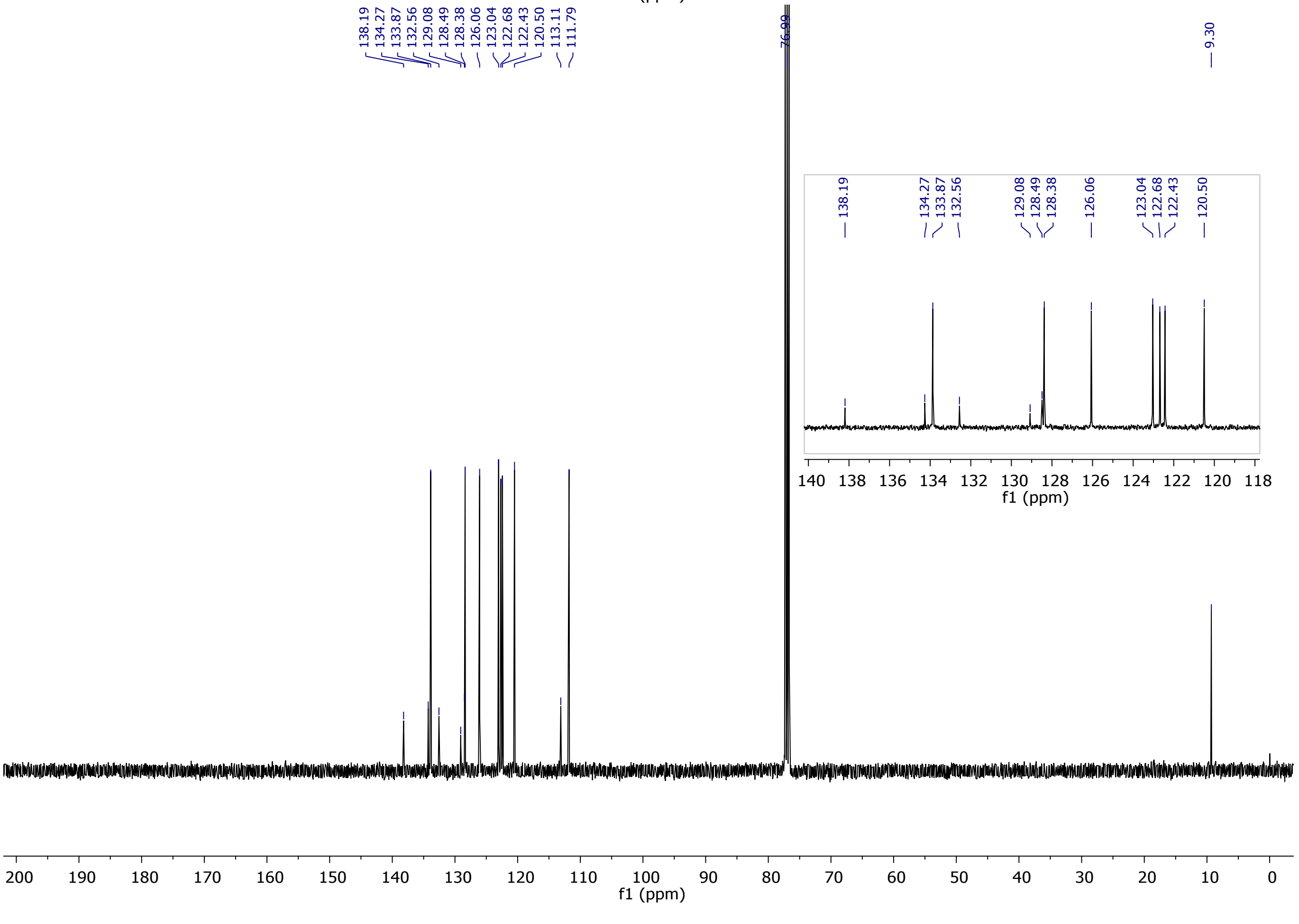

^{1}H-NMR (400 MHz, $CDCl_3$) and $^{13}C\{^{1}H\}$-NMR (100 MHz, $CDCl_3$) spectra for **15c**

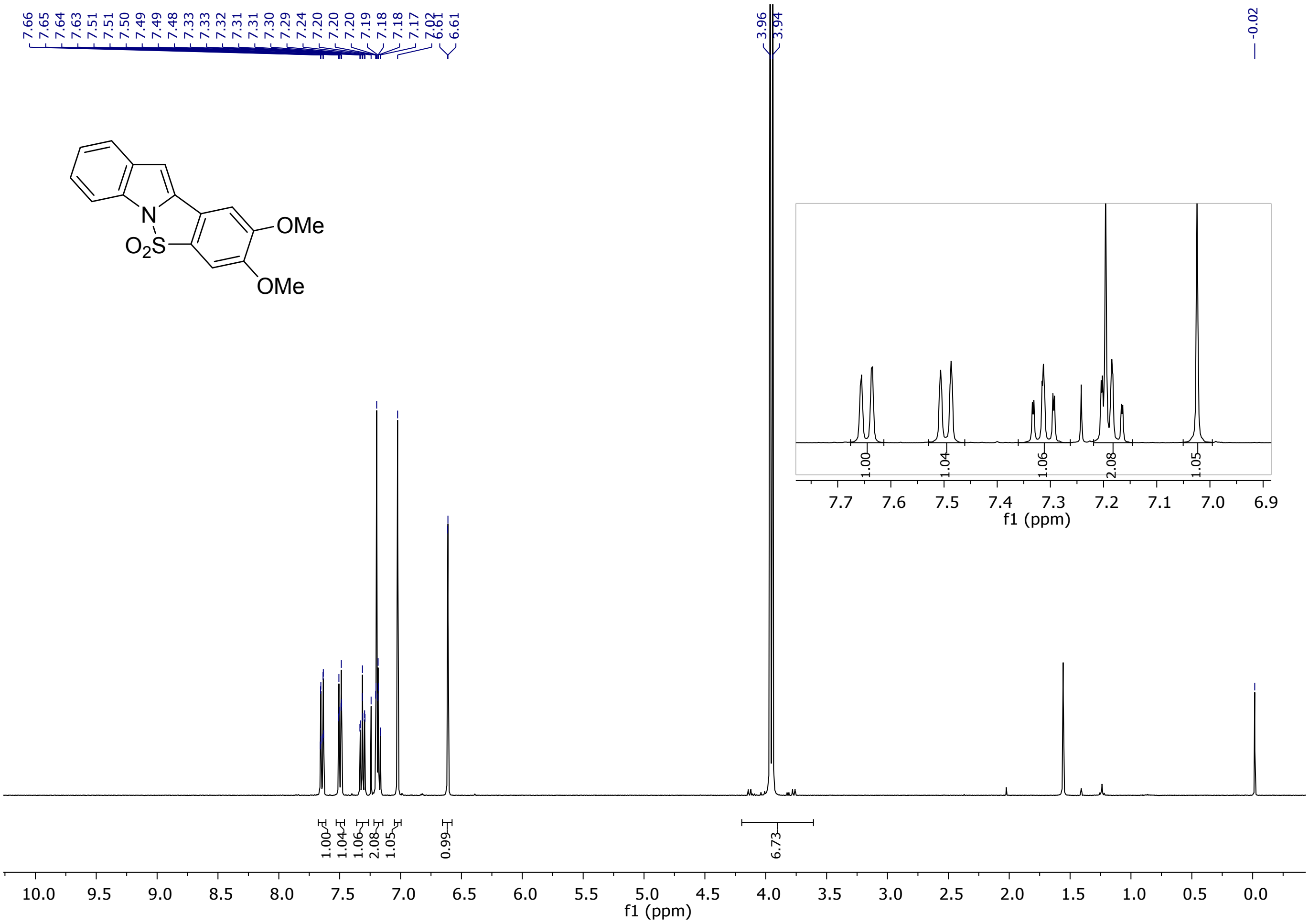

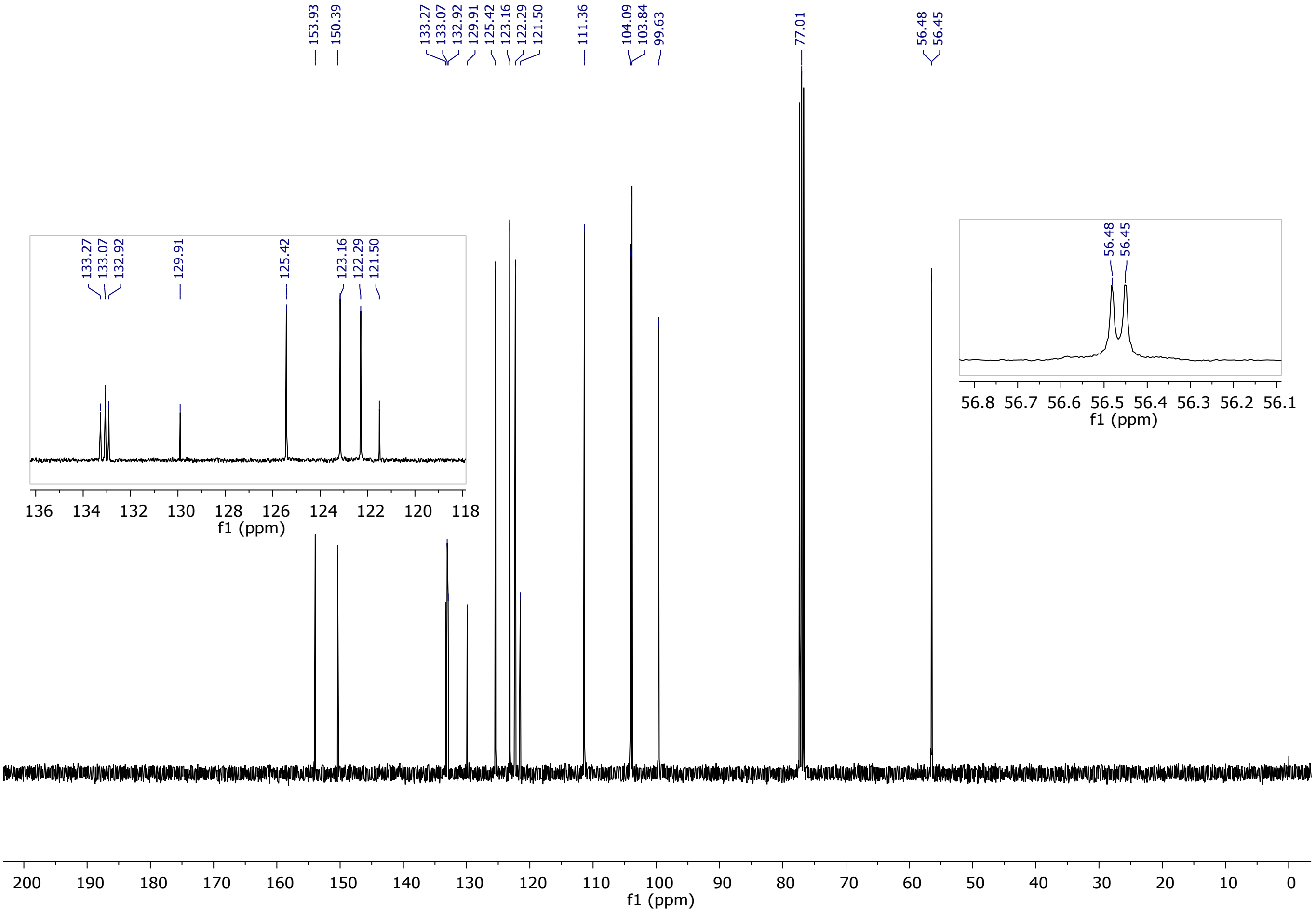

^{1}H-NMR (400 MHz, $CDCl_3$) and $^{13}C\{^{1}H\}$-NMR (100 MHz, $CDCl_3$) spectra for **16a**

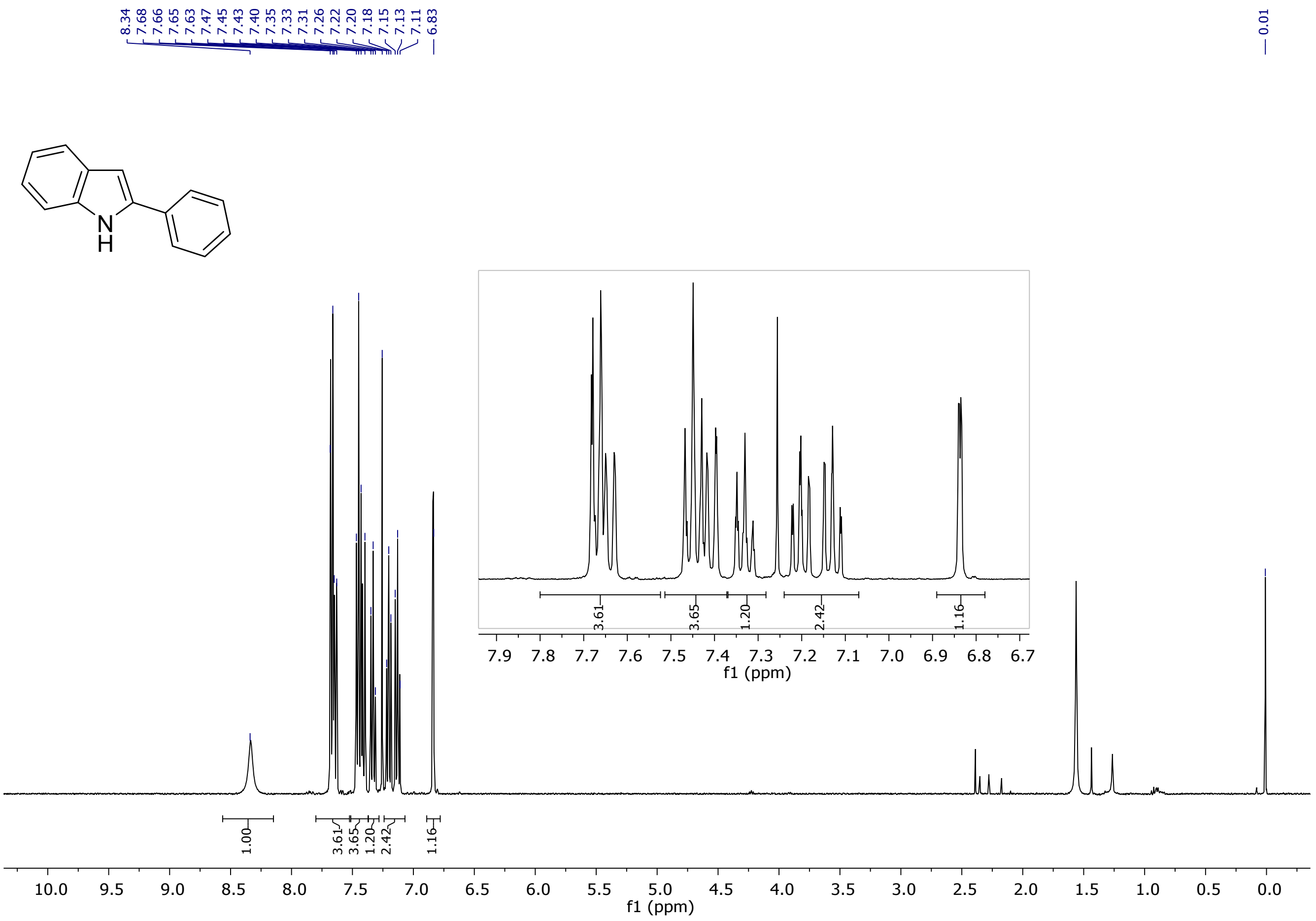

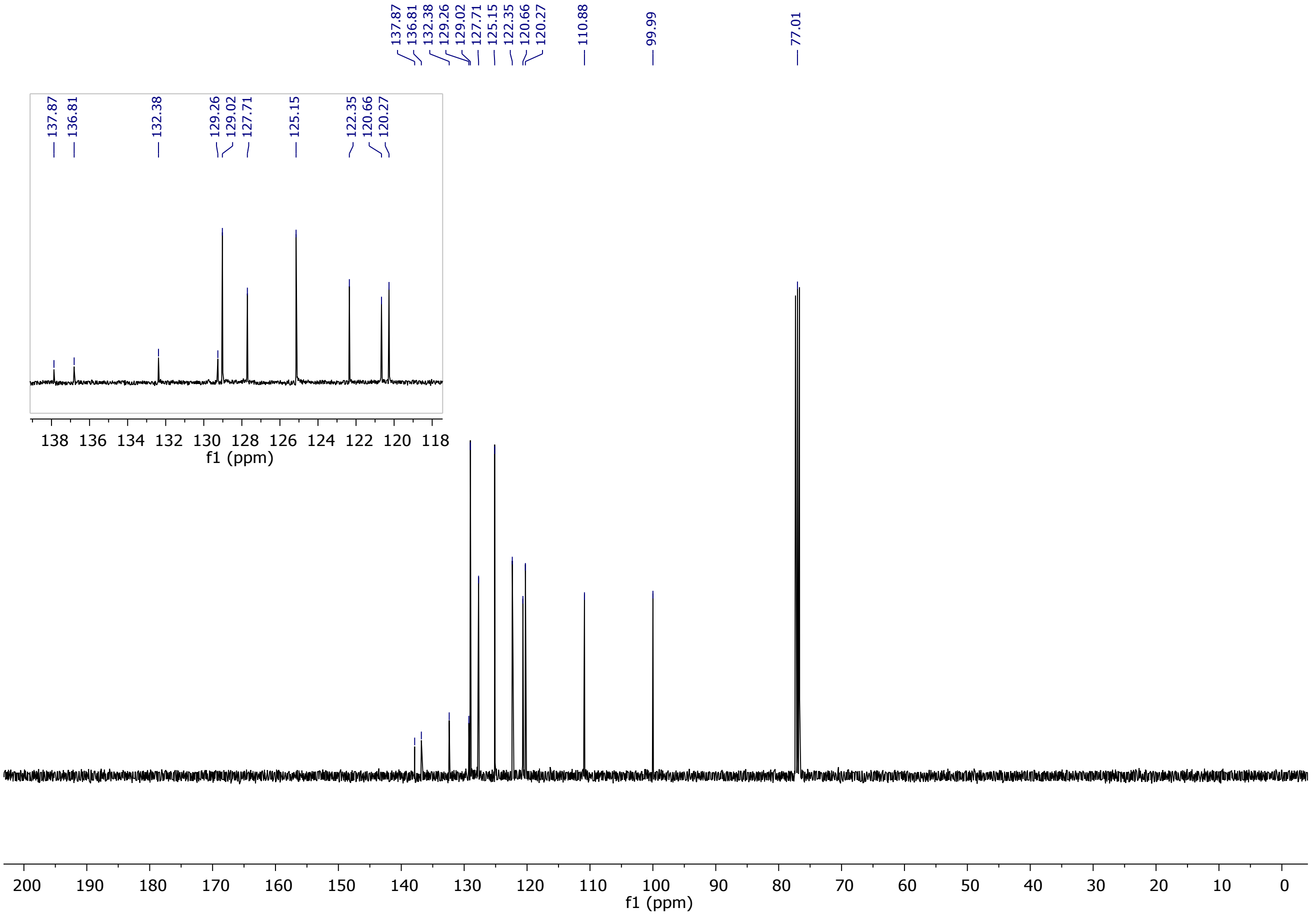

^{1}H-NMR (400 MHz, $CDCl_3$) and $^{13}C\{^1H\}$-NMR (100 MHz, $CDCl_3$) spectra for **16b**

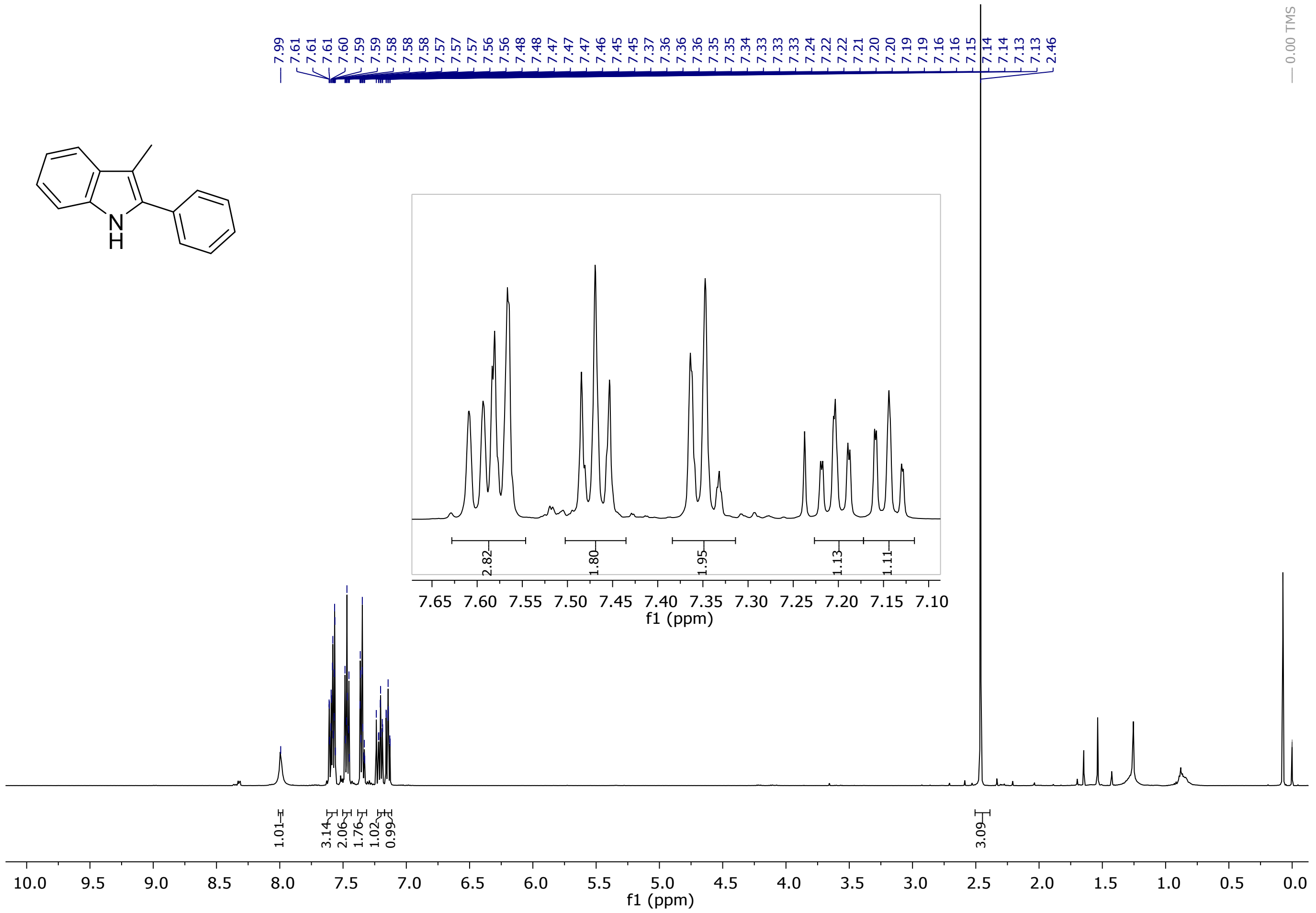

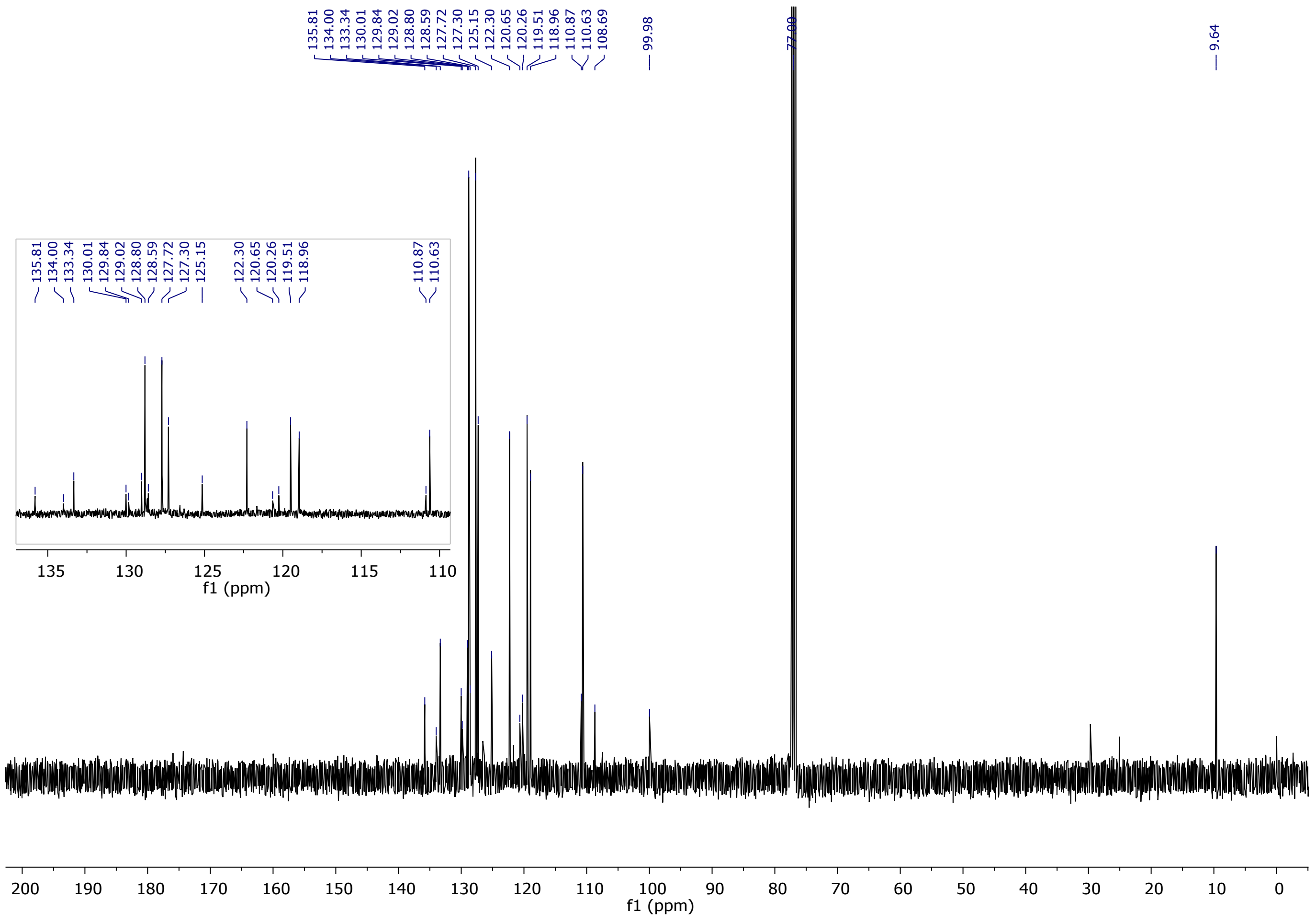

^{1}H-NMR (400 MHz, $CDCl_3$) and $^{13}C\{^{1}H\}$-NMR (100 MHz, $CDCl_3$) spectra for **16c**

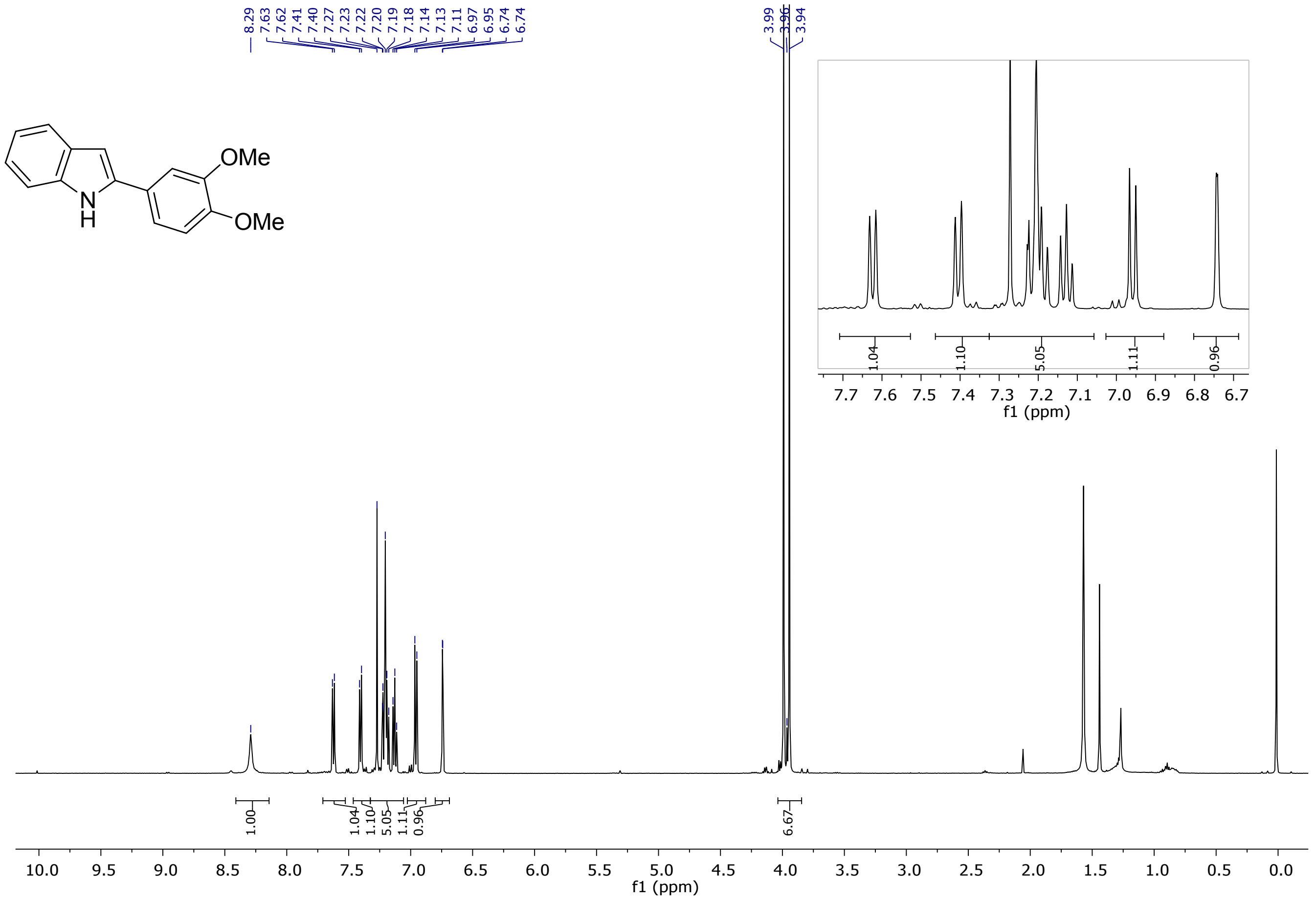

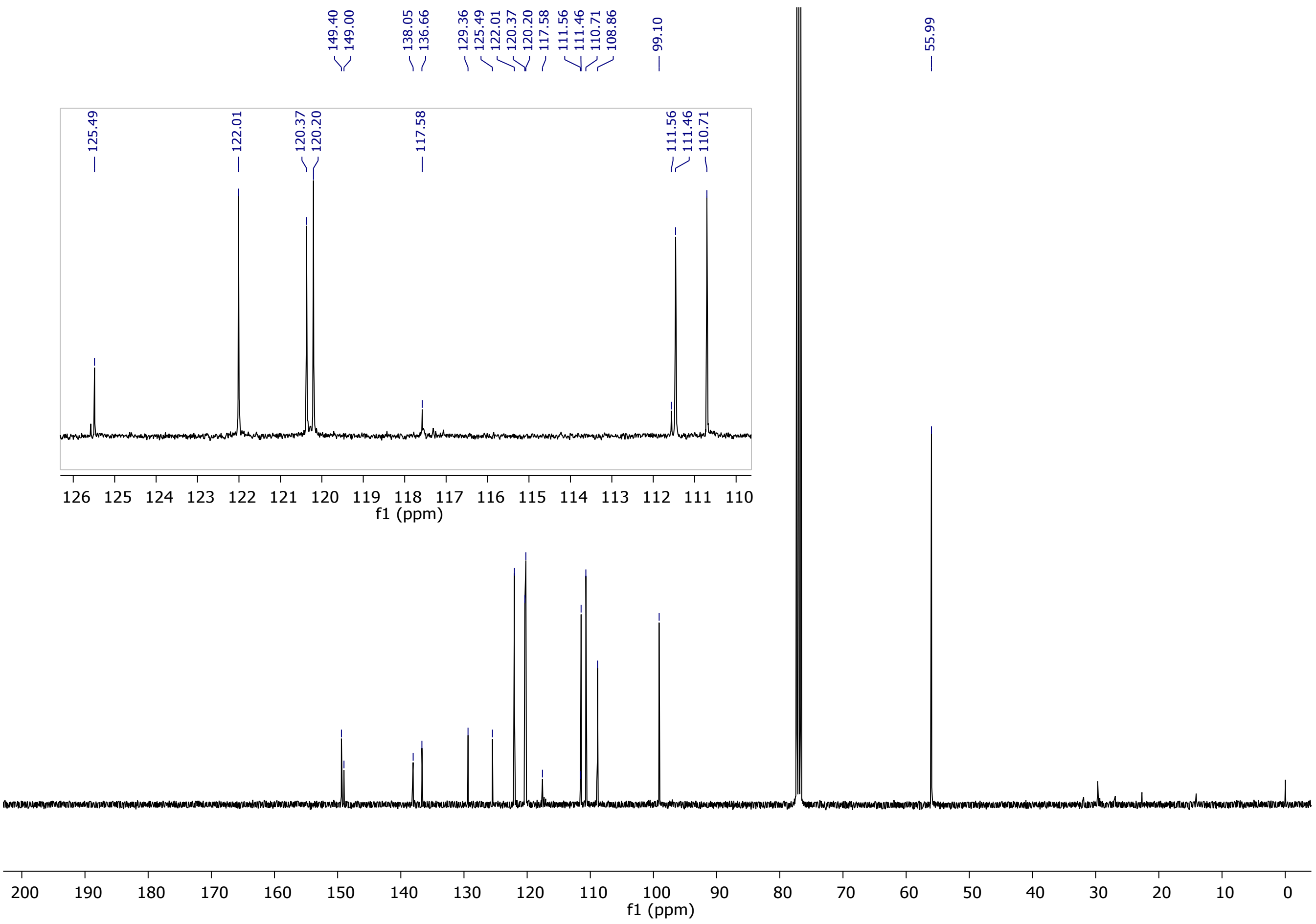

^{1}H-NMR (500 MHz, $CDCl_3$) and ^{13}C{^{1}H}-NMR (125 MHz, $CDCl_3$) spectra for D-**8a**

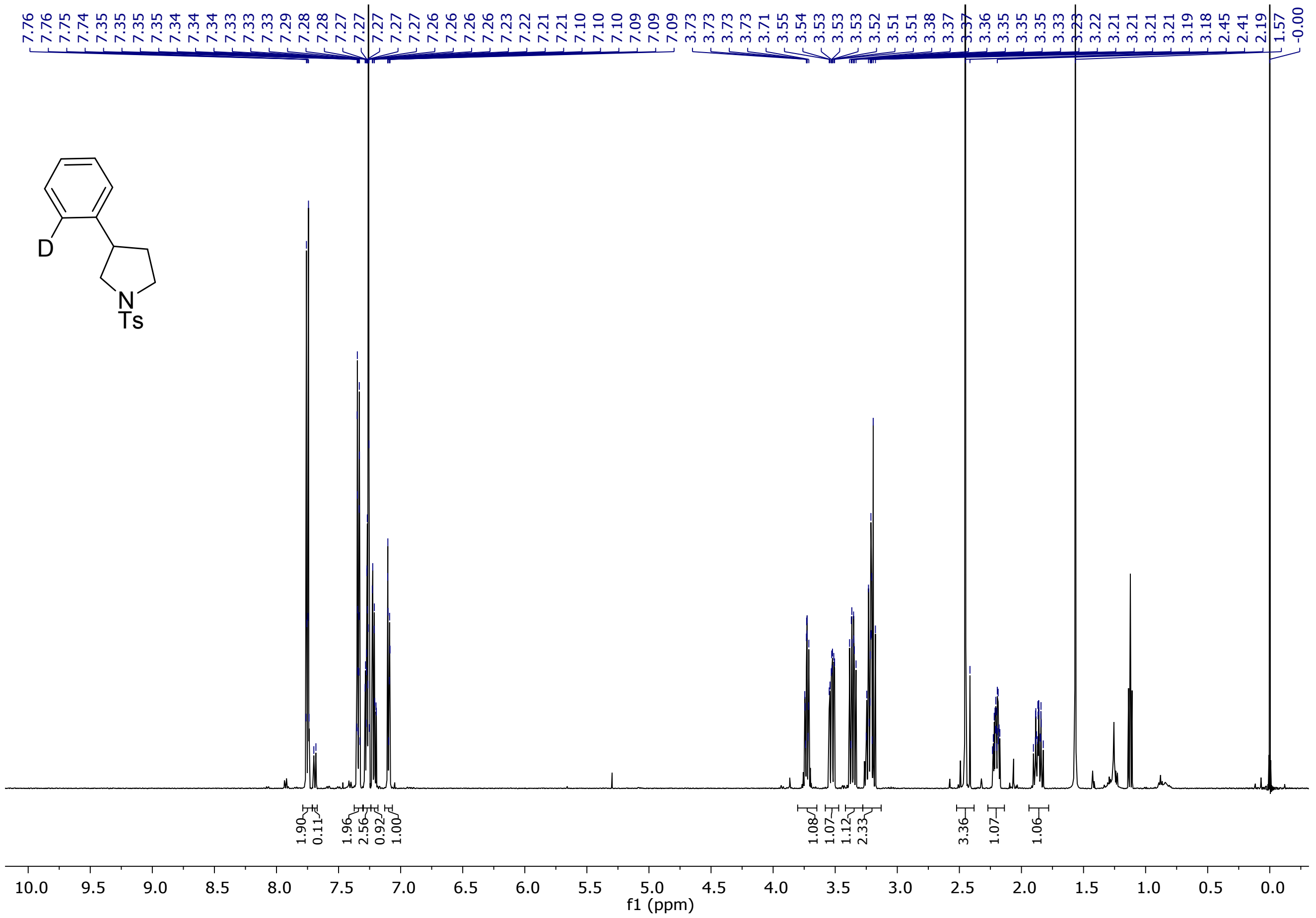

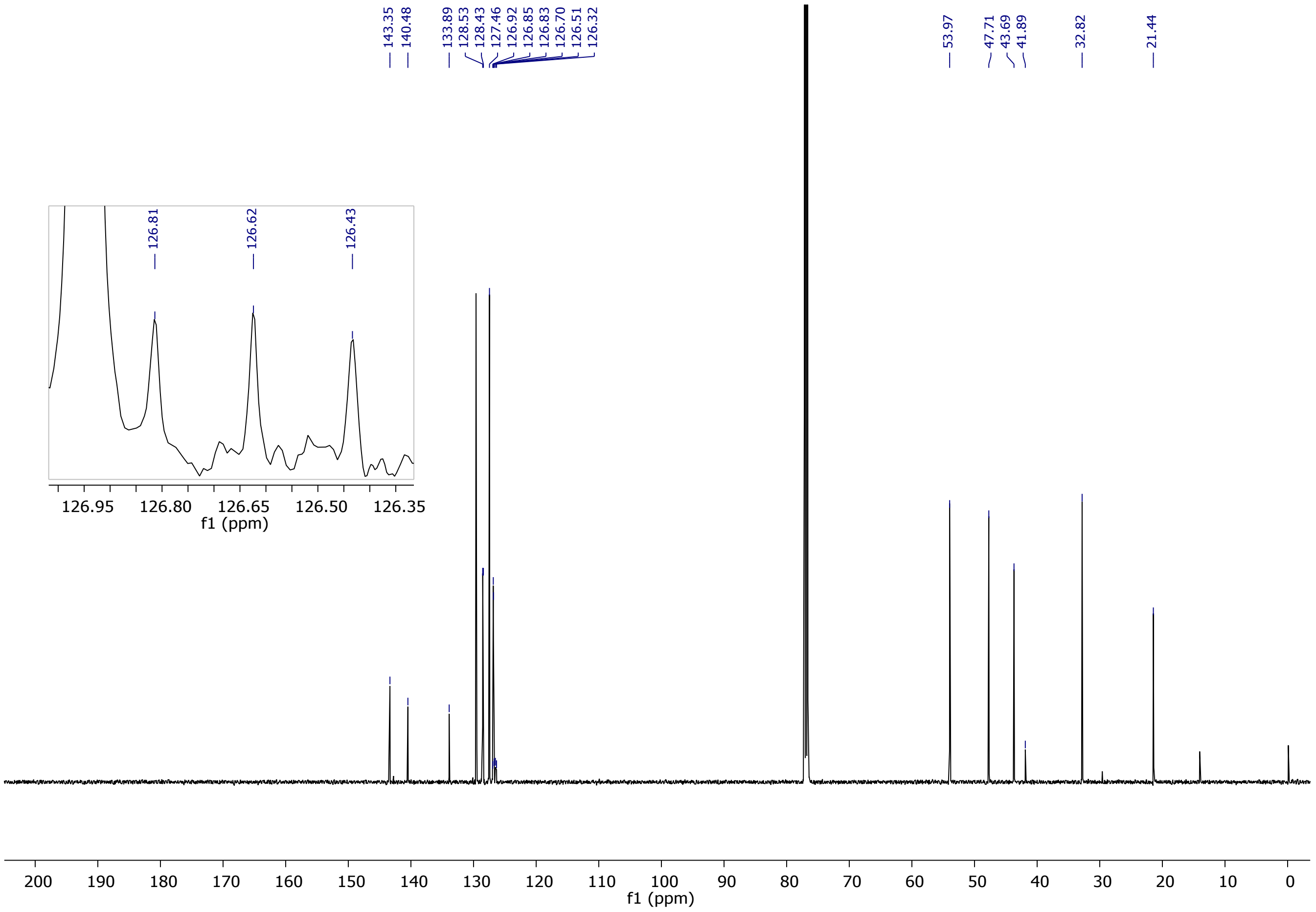

^{1}H-NMR (400 MHz, $CDCl_3$) and ^{13}C{^{1}H}-NMR (100 MHz, $CDCl_3$) spectra for D-**12a**

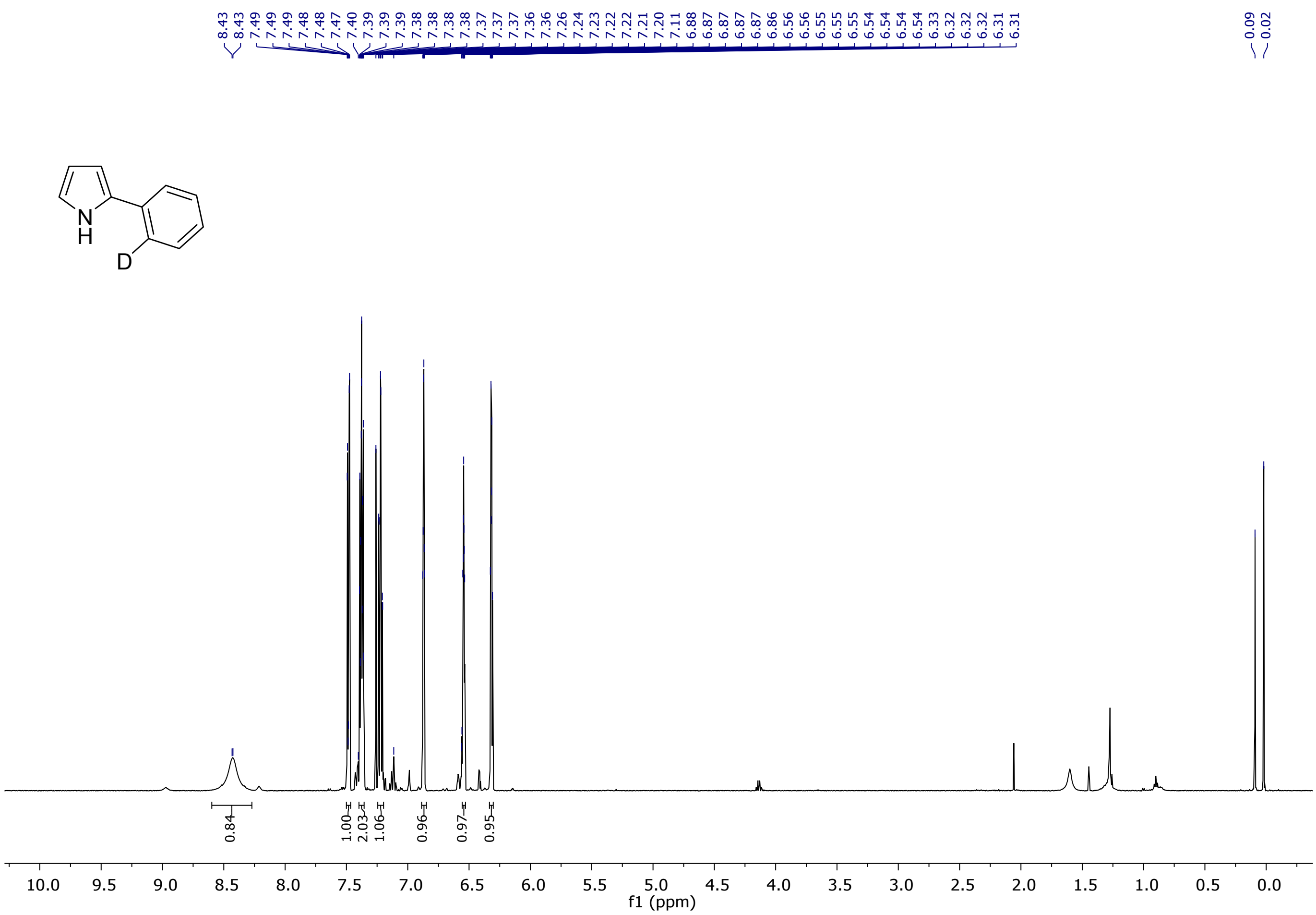

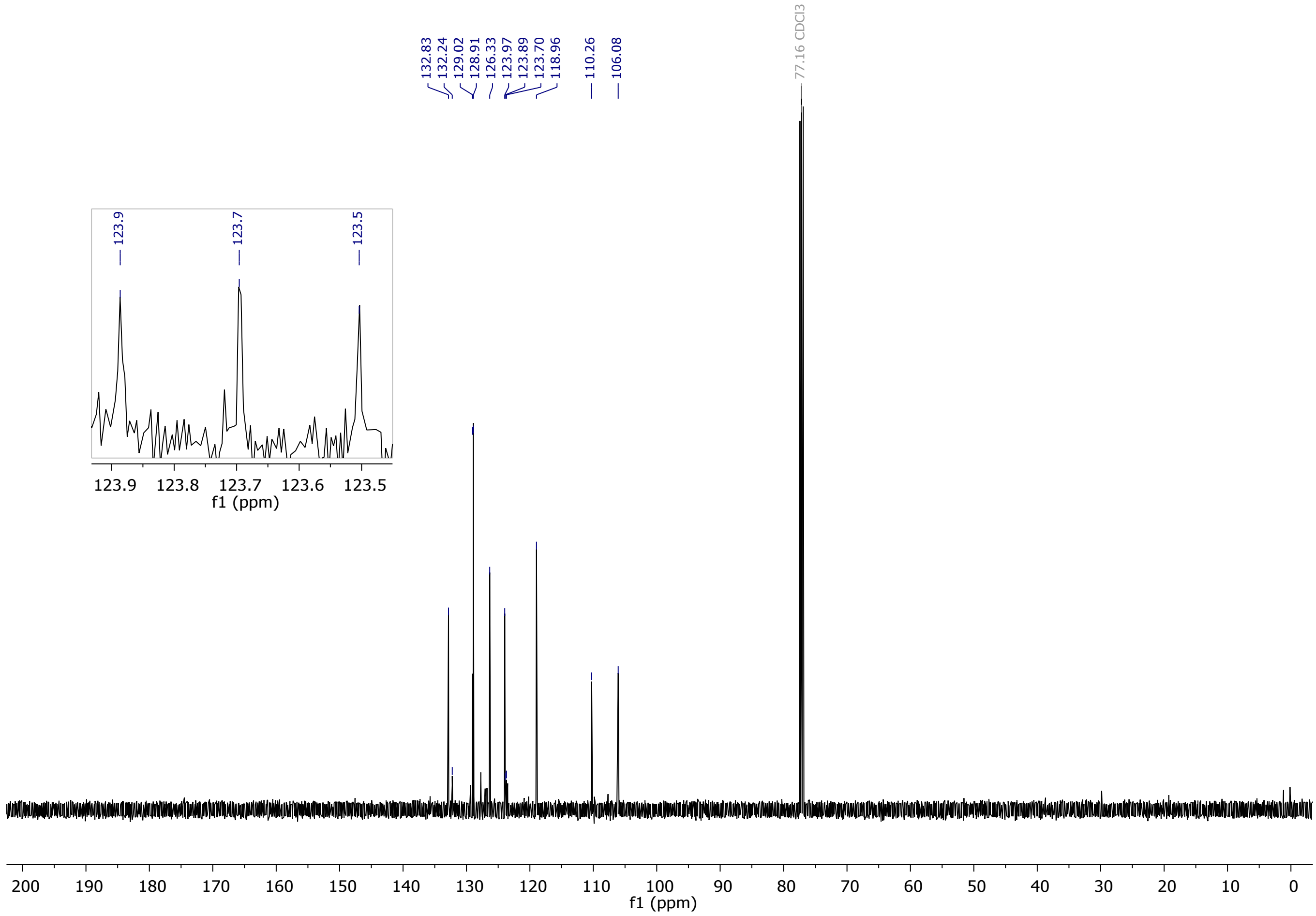

Comparison between the ^{1}H-NMR (400 MHz, $CDCl_3$) and $^{13}C\{^{1}H\}$-NMR (100 MHz, $CDCl_3$) spectra for D-**12a** (top) and **12a** (bottom)

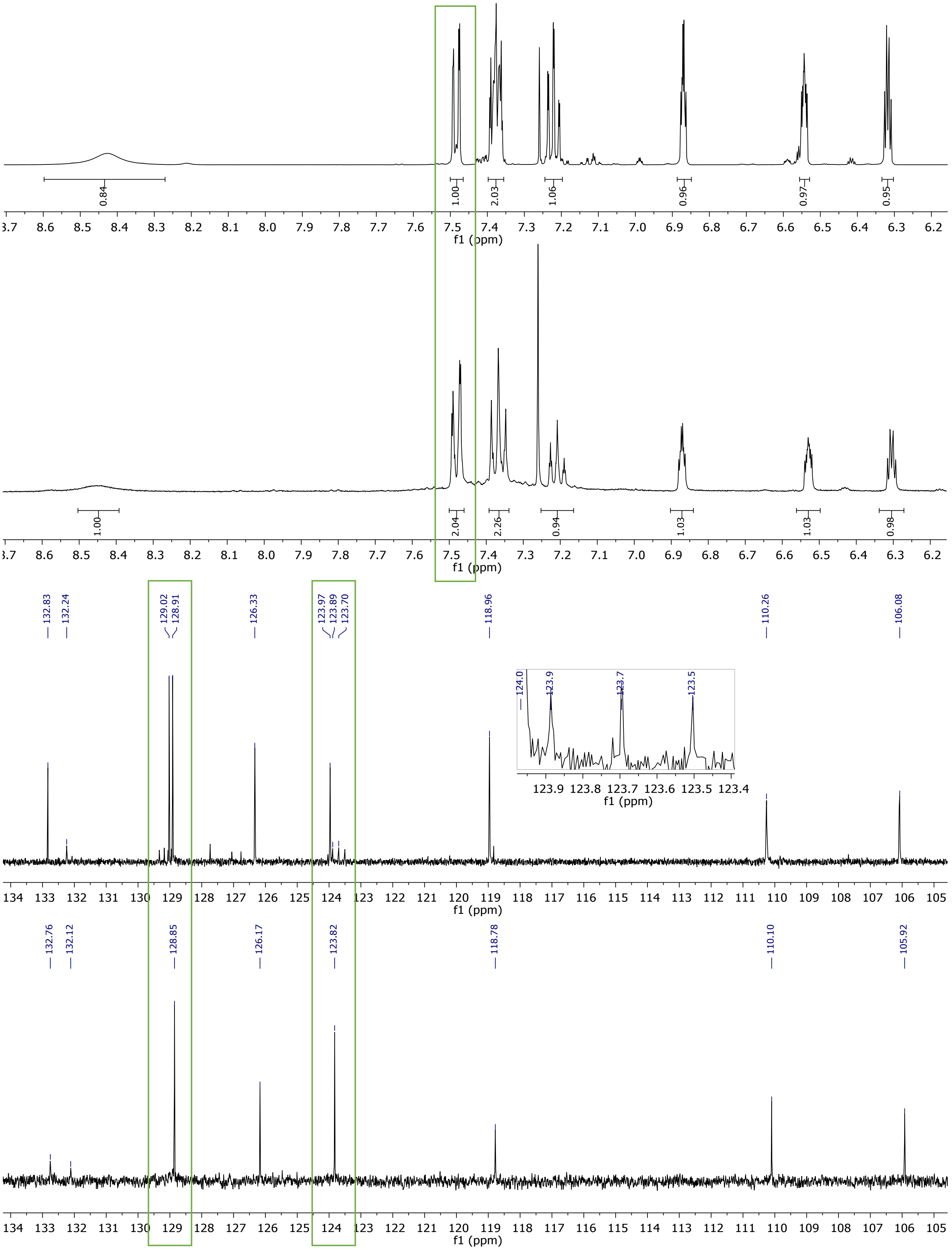

^{1}H-NMR (400 MHz, $CDCl_3$) and $^{13}C\{^{1}H\}$-NMR (100 MHz, $CDCl_3$) spectra for D-**16a**

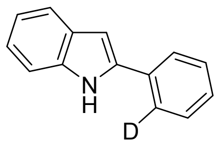

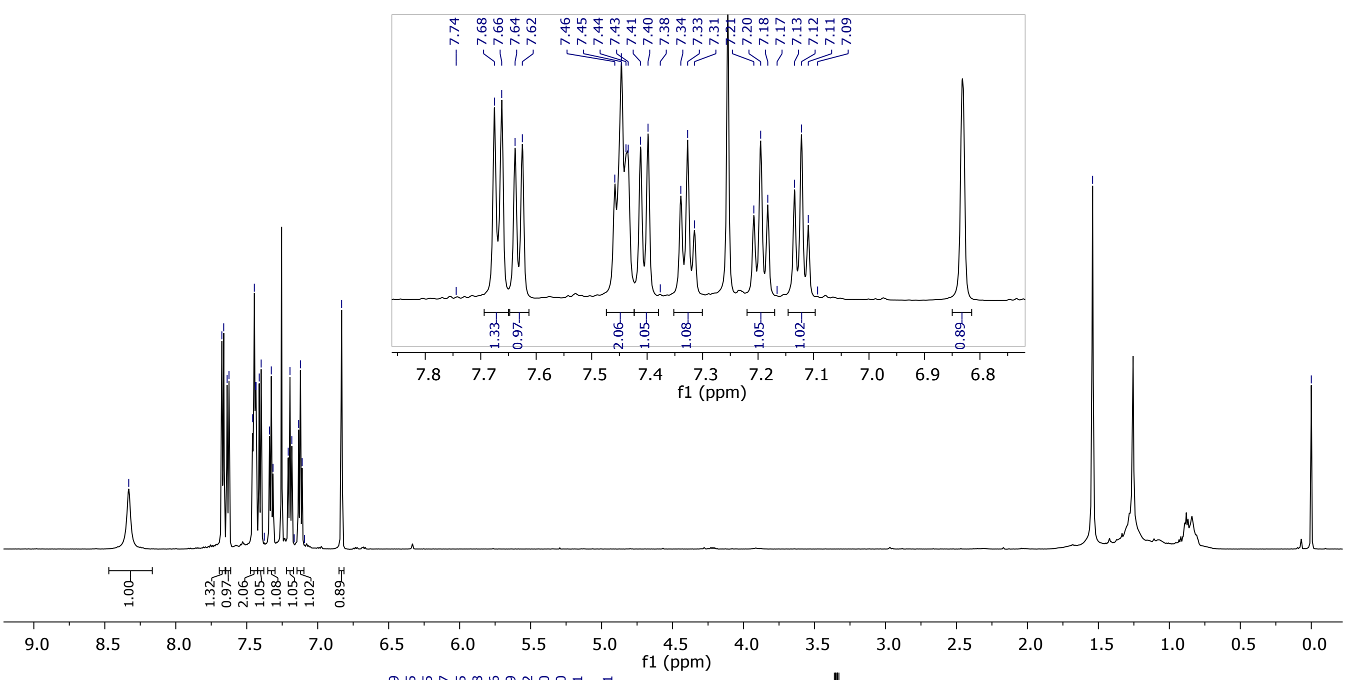

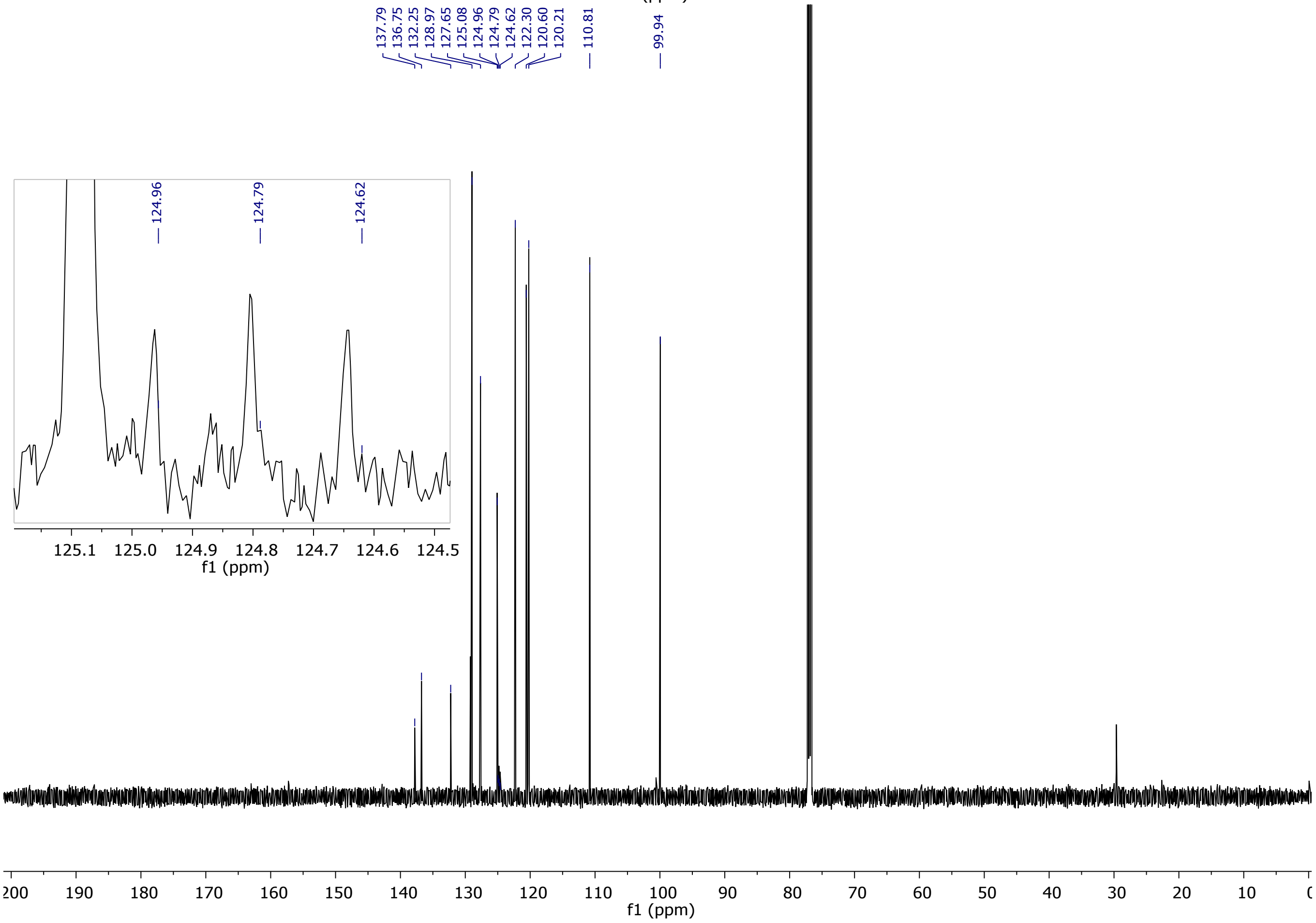

^{1}H-NMR (400 MHz, $CDCl_3$) and $^{13}C\{^1H\}$-NMR (100 MHz, $CDCl_3$) for D-**16c**

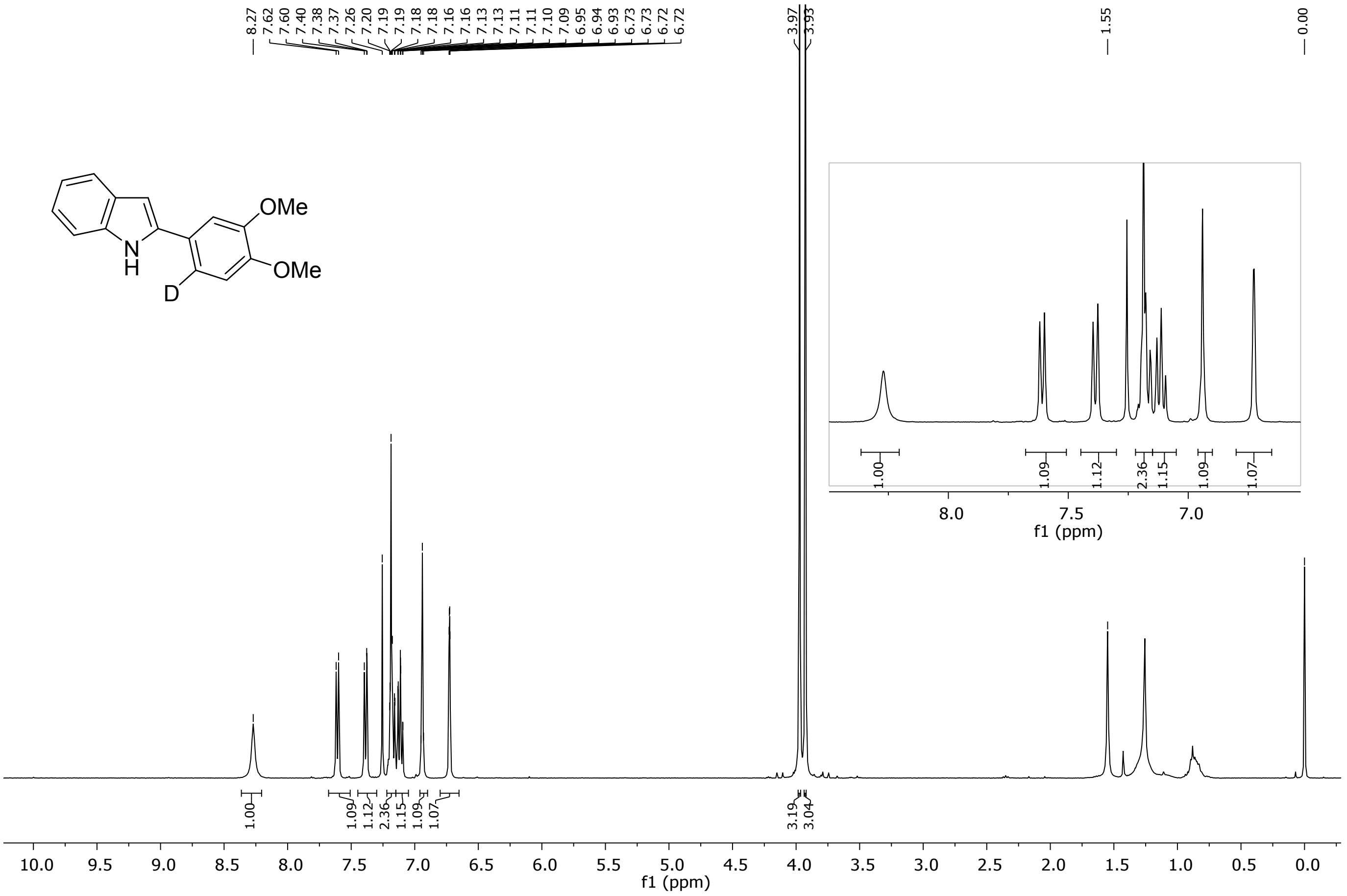

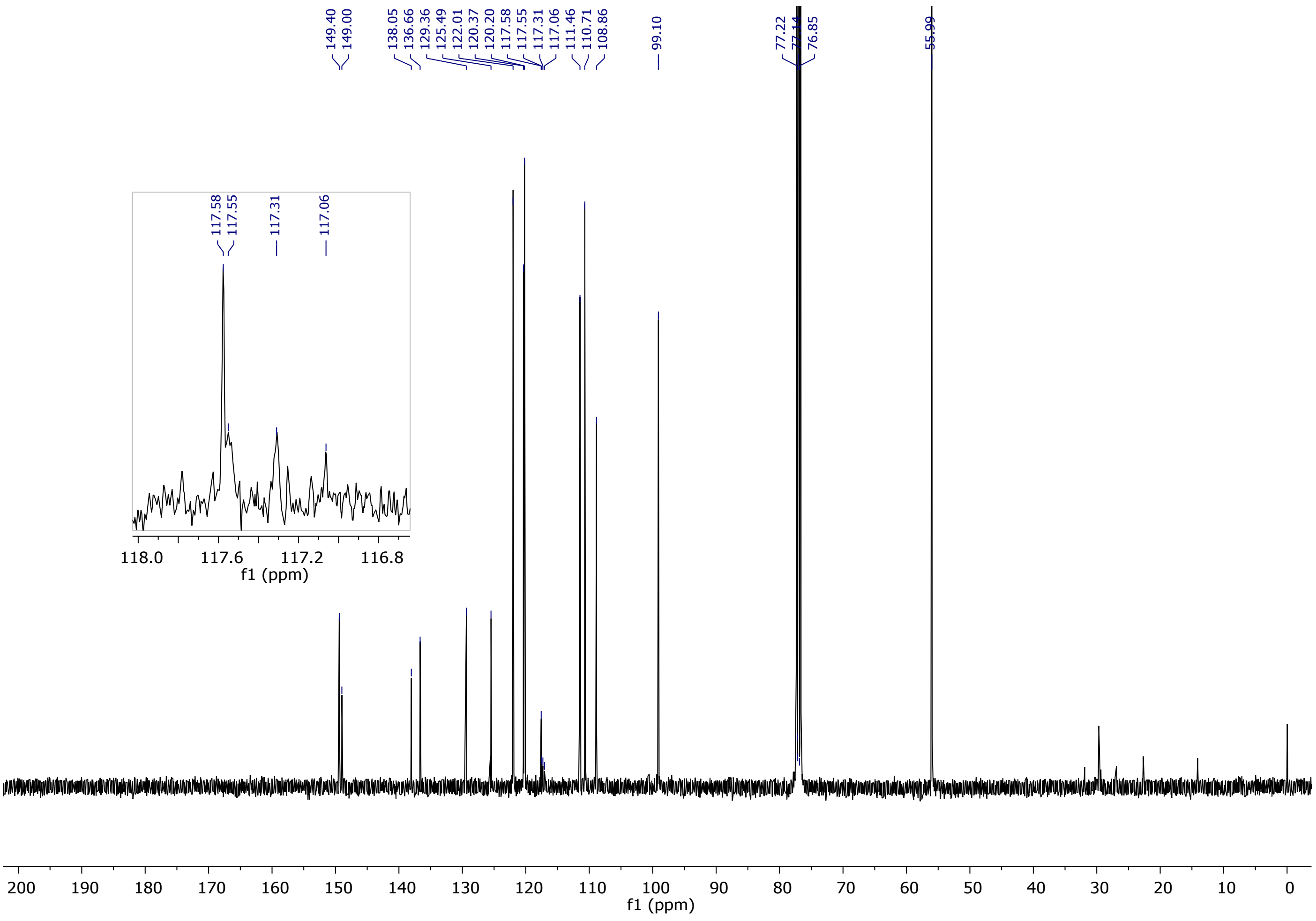

^{1}H-NMR ($CDCl_3$, 400 MHz) and ^{13}C{^{1}H}-NMR ($CDCl_3$, 100 MHz) spectra of **18**

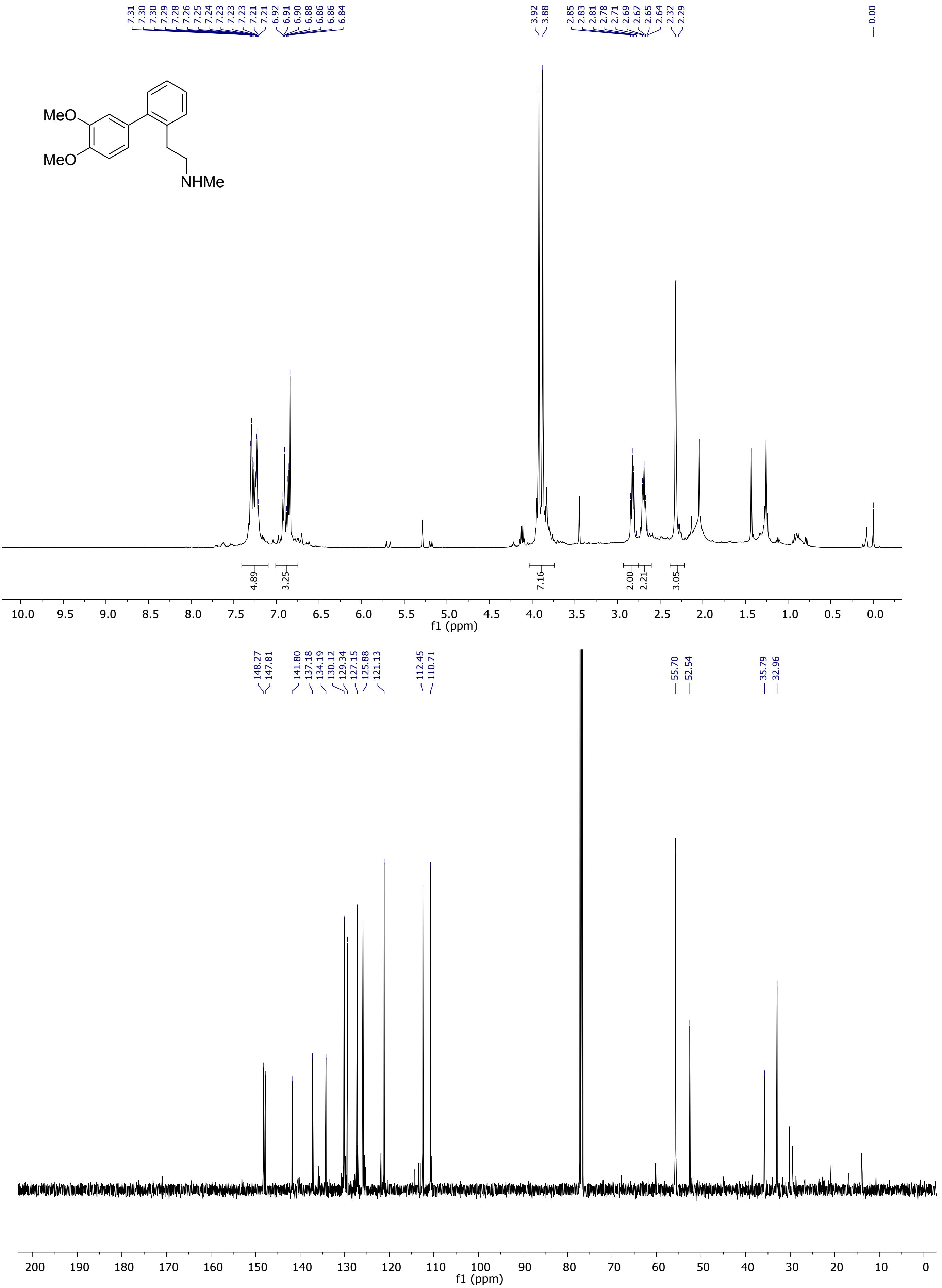

Computational Details

Table S1. LUMO orbital energies in a.u., and adiabatic electron affinities in kJ mol^{-1}, at CAM-B3LYP, WB97XD and M062X/def2TZVP levels for compounds **7a-d**, **11b**, **15c** and **17**.

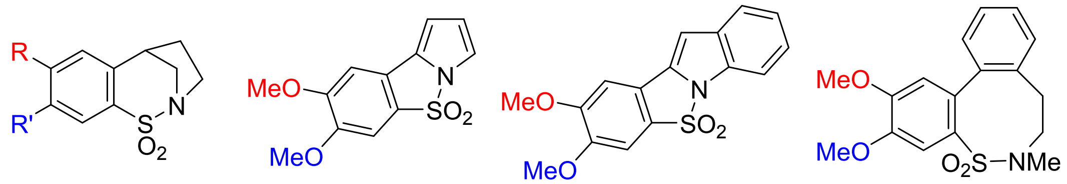

R = H, R' = H, **7a**
R = OMe, R' = OMe, **7b**
R = OMe, R' = H, **7c**
R = H, R' = OMe, **7d**

11b **15c** **17**

			LUMO			Adiabatic electron affinities		
**Entry**	**Cyclic sulfonamide**	**Yield[a]**	**CAM-B3LYP**	**WB97XD**	**M062X**	**CAM-B3LYP**	**WB97XD**	**M062X**
1	**7a**	71%	-0.0002	0.0240	-0.0119	-6.55	1.44	-6.59
2	**7b**	0%	0.0156	0.0399	0.0037	4.05	12.41	2.39
3	**7c**	0%	0.0101	0.0346	-0.0024	9.80	18.12	9.66
4	**7d**	80%	0.0007	0.0251	-0.0114	-8.69	-0.41	-9.81
5	**11b**	47%	-0.0012	0.0230	-0.0131	-6.45	1.91	-5.33
6	**15c**	81%	-0.0219	0.0011	-0.0335	-67.69	-59.30	-71.35
7	**17**	0%	0.0076	0.0315	-0.0054	-7.24	1.79	-13.16

[a]Observed, isolated yields of products from double reduction of the specified sulfonamide, see Schemes 2-5.

Figure S2. Comparative LUMO orbital energies, in a.u., at different computational levels using def2-TZVP basis set for compounds **7a-c**, **11b**, **15c** and **17**. (Note, below the dotted line the Mg-MeOH reduction reactions proceed with the compounds specified on the x-axis and above it they do not).

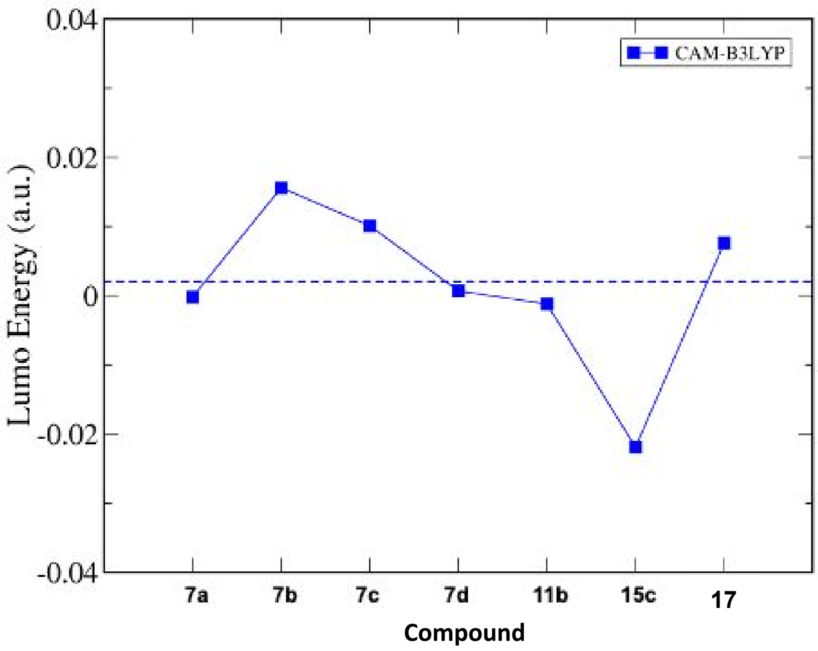

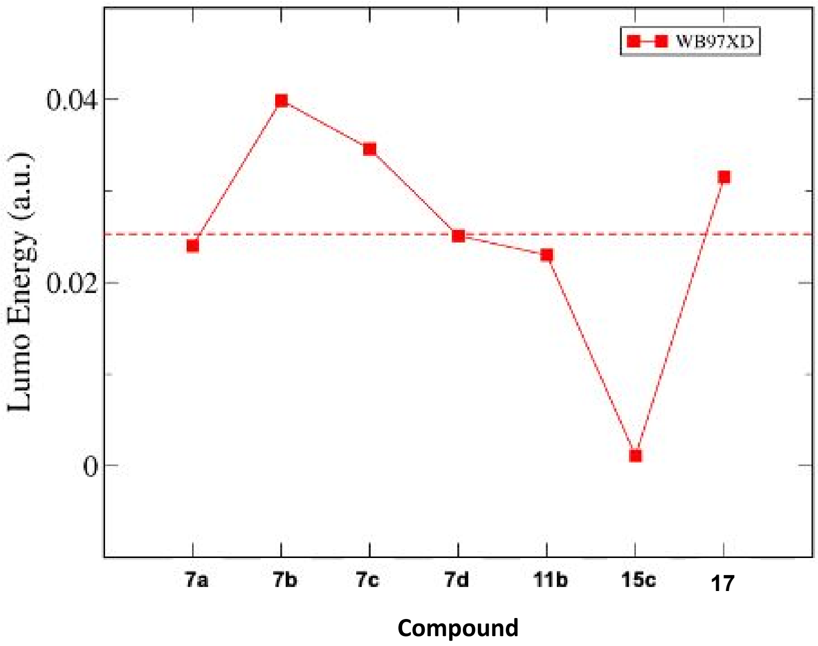
WB97XD
0.04
0.02
0
Lumo Energy (a.u.)
7a
7b
7c
7d
11b
15c
17
Compound

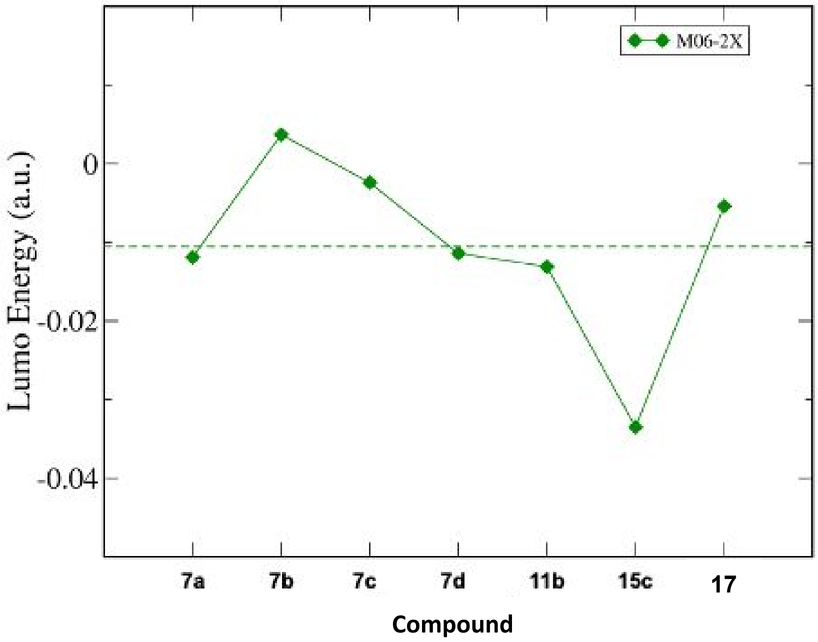
M06-2X
0
-0.02
-0.04
Lumo Energy (a.u.)
7a
7b
7c
7d
11b
15c
17
Compound

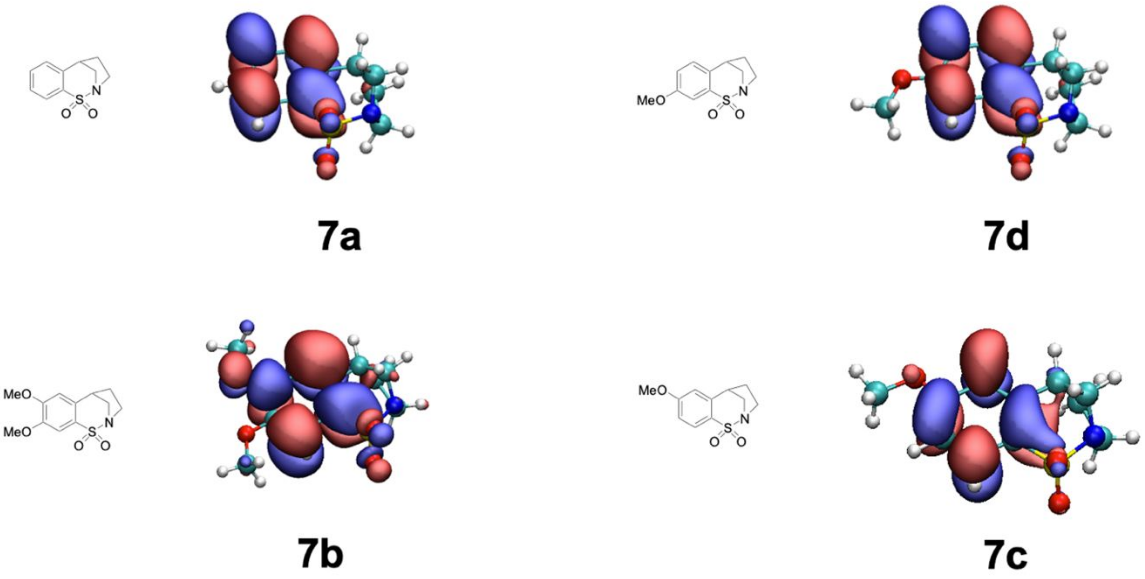

Figure S3. Depiction of LUMO orbitals for compounds **7a**-**d**, obtained with CAM-B3LYP/def2-TZVP

As depicted in Figure S3 below, the calculations indicate that the LUMO for the cyclic sulfonamides studied is located on the benzene ring which would imply that the identity and the position of the aromatic substituents (relative to the sulfonyl group) will affect the stability of the LUMO and therefore the orbital energy.

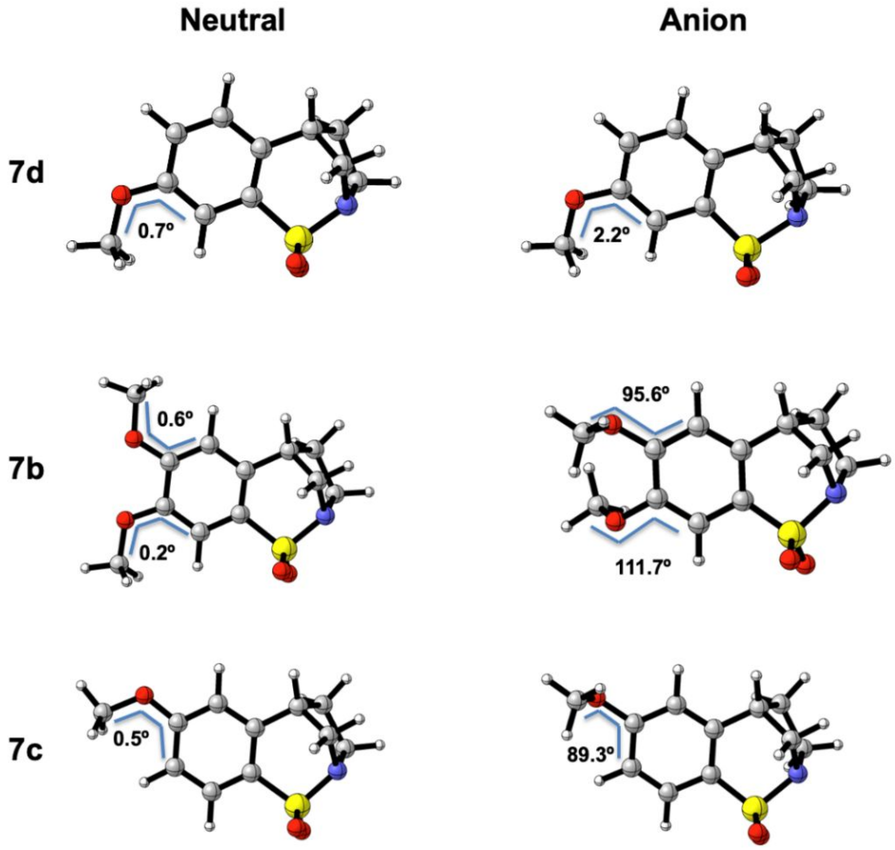

Figure S3. Depiction of neutral (left-hand side) and anionic (right-hand side) structures for compounds **7b-d**, demonstrating the change in conformation of the methoxy substituents, indicated by the torsion angles - obtained with CAM-B3LYP/def2-TZVP.

For compounds **7a**-**d**, it is also observed that the conformation of the OMe groups vary from the neutral species to the radical anions. Considering compound **7d**, for which the Mg-MeOH reaction works, in the neutral species the OMe is aligned with the molecular plane of the benzene ring. Upon acceptance of the electron in the radical anionic species, the OMe remains within the molecular plane (Figure S4). In contrast, for **7b** and **7c** (where the Mg-MeOH reaction does not work), the OMe groups are in the molecular plane in the neutral species but then they rotate and move out of the molecular plane on acceptance of an electron. We propose that this change in conformation is forced as electrons are added into the LUMO and is indicative of a destabilisation of the key reactive intermediate when the methoxy group is *para*-positioned relative to the sulfonyl group.

Table S2. Cartesian coordinates, imaginary frequencies and total energy at CAM-B3LYP /def2TZVP level for compounds **7a**-**d**, **11b**, **15c** and **18** (both neutral and anionic species).

Compound	Coordinates			
**7a** neutral	C	-0.705256	-0.491911	0.000356
Imaginary frequencies=0	C	-1.770805	-1.341049	-0.253063
SCF energy=-991.0132792	C	-3.056225	-0.837272	-0.278983
	C	-3.267429	0.512301	-0.044702
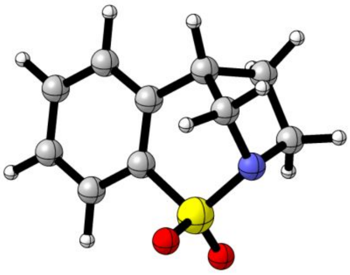	C	-2.198512	1.351783	0.209261
	C	-0.896054	0.868811	0.238329
	H	-1.586104	-2.391342	-0.434960
	H	-3.890882	-1.494655	-0.480541
	H	-4.272438	0.912904	-0.057812
	H	-2.373813	2.404265	0.395024
	C	0.268058	1.800926	0.487897
	H	-0.081352	2.688147	1.013758
	C	1.378846	1.060139	1.230509
	H	2.184000	1.732758	1.529286
	C	0.983858	2.156656	-0.830148
	H	1.519539	3.099431	-0.719103
	C	1.974200	0.986772	-1.037350
	H	1.759971	0.378737	-1.910768
	H	2.999373	1.343465	-1.123286
	N	1.882409	0.156141	0.182871
	H	0.288754	2.264738	-1.660041
	H	1.053386	0.498432	2.101737
	S	0.929793	-1.187118	0.060822
	O	1.047833	-1.918374	1.281762
	O	1.212408	-1.823991	-1.186659
**7a** anion	C	-0.642545	-0.486883	-0.140667
Imaginary frequencies=0	C	-1.761431	-1.349106	-0.409757
SCF energy=-991.0087237	C	-3.037651	-0.875253	-0.327401
	C	-3.309311	0.461398	0.043558
	C	-2.209199	1.310805	0.313215
	C	-0.914395	0.882231	0.237998
	H	-1.575400	-2.375910	-0.698171
	H	-3.861105	-1.543521	-0.558541
	H	-4.324225	0.825756	0.123493
	H	-2.390864	2.351845	0.567030

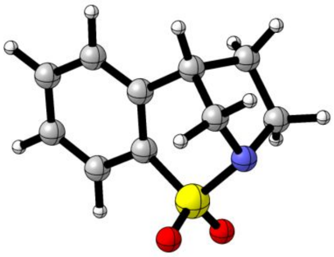	C	0.237665	1.833332	0.420700
	H	-0.108326	2.748284	0.904019
	C	1.371268	1.141606	1.183669
	H	2.168598	1.843176	1.450754
	C	0.944358	2.125130	-0.921152
	H	1.465179	3.085227	-0.875625
	C	1.954895	0.963938	-1.067437
	H	1.738274	0.305059	-1.903539
	H	2.975371	1.337502	-1.187486
	N	1.890320	0.195294	0.186852
	H	0.232743	2.163491	-1.743410
	H	1.053128	0.612135	2.077609
	S	0.899583	-1.167655	0.106079
	O	1.065924	-1.817216	1.389505
	O	1.333967	-1.942885	-1.034220
**7b** neutral<br>Imaginary frequencies=0<br>SCF energy=-1220.0608331<br>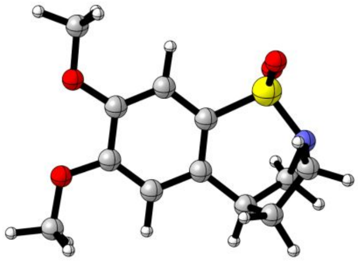				
	C	0.285549	0.411028	-0.100545
	C	-0.820894	1.248932	0.033616
	C	-2.090262	0.721522	0.060819
	C	-2.254443	-0.676397	-0.053538
	C	-1.145478	-1.486724	-0.187074
	C	0.146882	-0.959392	-0.216868
	H	-0.663944	2.313086	0.124558
	H	-1.269965	-2.555784	-0.279196
	C	1.342939	-1.877635	-0.335047
	H	1.041315	-2.818503	-0.793454
	C	2.463233	-1.172032	-1.096871
	H	3.301870	-1.840828	-1.296484
	C	2.013993	-2.091813	1.035594
	H	2.588494	-3.018449	1.029658
	C	2.951504	-0.869653	1.177052
	H	2.672840	-0.193022	1.978891
	H	3.983118	-1.176225	1.343219
	N	2.890870	-0.158045	-0.118373
	H	1.288175	-2.154265	1.843623
	H	2.159609	-0.704724	-2.029455
	S	1.890373	1.156595	-0.161764
	O	2.045901	1.780871	-1.437822
	O	2.102125	1.918107	1.030144
	O	-3.526187	-1.115931	-0.023902
	C	-3.764714	-2.501674	-0.132062
	H	-4.842799	-2.625170	-0.083047
	H	-3.398752	-2.895814	-1.083513
	H	-3.300144	-3.051262	0.690596
	O	-3.223987	1.438612	0.192412
	C	-3.113335	2.842046	0.298664
	H	-2.645361	3.272135	-0.590296
	H	-4.128725	3.217153	0.388987
	H	-2.540459	3.129941	1.183668
**7b** anion<br>Imaginary frequencies=0<br>SCF energy=-1220.0505833				
	C	0.396802	-0.429185	0.002727
	C	-0.783694	-1.257844	-0.047617
	C	-2.020691	-0.685768	-0.082276

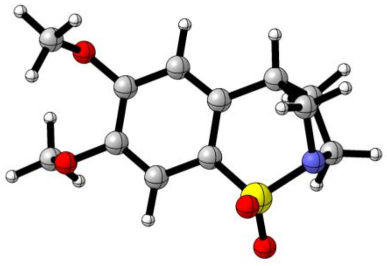	C	-2.183256	0.714490	-0.069348
	C	-1.025915	1.530813	-0.025121
	C	0.231230	1.000632	0.011567
	H	-0.699400	-2.334981	-0.089387
	H	-1.157896	2.607555	-0.057184
	C	1.448420	1.885305	-0.055027
	H	1.172461	2.914728	0.176831
	C	2.541947	1.332864	0.862990
	H	3.390207	2.020527	0.942524
	C	2.149182	1.771809	-1.425599
	H	2.733583	2.672813	-1.628739
	C	3.076576	0.544744	-1.265902
	H	2.805570	-0.289292	-1.906826
	H	4.118165	0.804416	-1.472114
	N	2.976064	0.128530	0.143051
	H	1.428050	1.646837	-2.230735
	H	2.202186	1.074682	1.862298
	S	1.894021	-1.135090	0.412143
	O	2.034706	-1.450982	1.817120
	O	2.249331	-2.199508	-0.497959
	O	-3.446974	1.293228	0.000375
	C	-3.998896	1.291383	1.299838
	H	-3.364702	1.849308	1.997529
	H	-4.977181	1.773659	1.240565
	H	-4.120577	0.270940	1.673880
	O	-3.141103	-1.502681	-0.116974
	C	-3.849780	-1.451223	-1.335580
	H	-3.211835	-1.769832	-2.167369
	H	-4.688614	-2.142697	-1.245346
	H	-4.226037	-0.445520	-1.535983
**7c** neutral<br>Imaginary frequencies=0<br>SCF energy=-1105.5405478<br>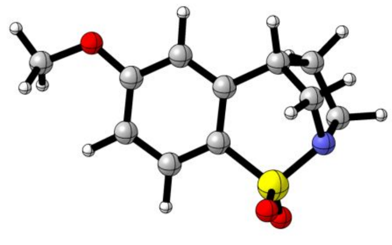	C	0.011777	-0.576194	0.022139
	C	-0.988627	-1.501130	-0.208106
	C	-2.317610	-1.120153	-0.206067
	C	-2.638879	0.209963	0.038360
	C	-1.628589	1.138002	0.271006
	C	-0.298974	0.765615	0.267846
	H	-0.727598	-2.533868	-0.397850
	H	-3.082833	-1.857378	-0.392610
	H	-1.912762	2.164918	0.460557
	C	0.785248	1.796067	0.487918
	H	0.373680	2.652846	1.018836
	C	1.972803	1.155789	1.205456
	H	2.722597	1.897464	1.484515
	C	1.435585	2.203226	-0.848952
	H	1.888284	3.190376	-0.755383
	C	2.520009	1.123793	-1.074867
	H	2.339949	0.494714	-1.941104
	H	3.507547	1.569008	-1.186094
	N	2.529501	0.294604	0.149194
	H	0.713462	2.244259	-1.661605
	H	1.717182	0.571531	2.085113
	S	1.694448	-1.128554	0.051814

O	1.908089	-1.840752	1.271899
O	2.011206	-1.747186	-1.197541
O	-3.898537	0.693446	0.070691
C	-4.973915	-0.196765	-0.146208
H	-5.878254	0.400040	-0.069538
H	-4.922203	-0.647839	-1.139832
H	-4.995759	-0.984757	0.610064

7c anion
Imaginary frequencies=0
SCF energy=-1105.5281703

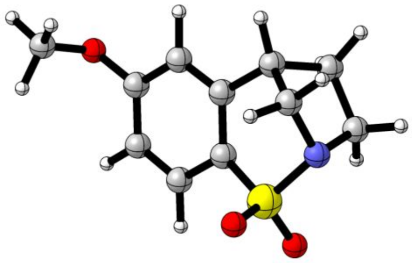

C	0.087849	-0.585170	-0.253924
C	-0.881510	-1.572398	-0.650209
C	-2.208254	-1.258829	-0.709303
C	-2.658668	0.031672	-0.369901
C	-1.720845	1.016068	0.008343
C	-0.383839	0.743898	0.075348
H	-0.540772	-2.560439	-0.931449
H	-2.930501	-1.995776	-1.043451
H	-2.074355	2.023958	0.204321
C	0.623807	1.828622	0.344249
H	0.126088	2.706121	0.759533
C	1.743271	1.290846	1.241408
H	2.421071	2.087897	1.564326
C	1.429442	2.172248	-0.928026
H	1.826320	3.188586	-0.862501
C	2.578536	1.137373	-0.930056
H	2.530802	0.440966	-1.762443
H	3.555295	1.627796	-0.955194
N	2.470387	0.391845	0.335095
H	0.809260	2.106857	-1.819619
H	1.396563	0.746743	2.115649
S	1.654472	-1.078616	0.195416
O	1.743216	-1.667845	1.515401
O	2.300260	-1.829015	-0.857576
O	-4.019588	0.339527	-0.403061
C	-4.685444	0.085363	0.809983
H	-4.263268	0.677514	1.630249
H	-5.734817	0.358442	0.675592
H	-4.623122	-0.973200	1.087071

7d neutral
Imaginary frequencies=0
SCF energy=-1105.5393865

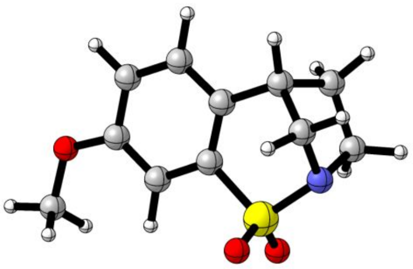

C	0.236228	-0.082822	-0.109103
C	1.565029	-0.463769	0.027055
C	2.550369	0.507292	0.014613
C	2.189136	1.844752	-0.138182
C	0.866206	2.196462	-0.272205
C	-0.149184	1.242068	-0.264260
H	1.802647	-1.509761	0.148210
H	0.606469	3.240923	-0.393862
C	-1.601179	1.646316	-0.386309
H	-1.670677	2.617329	-0.874668
C	-2.384723	0.553564	-1.110736
H	-3.412426	0.857251	-1.314854
C	-2.299056	1.640941	0.987680
H	-3.176833	2.287038	0.963056
C	-2.714942	0.163763	1.179850

	H	-2.200329	-0.335146	1.995169
	H	-3.785473	0.070126	1.355236
	N	-2.402049	-0.515684	-0.097092
	H	-1.646207	1.995527	1.782371
	H	-1.934755	0.202207	-2.035065
	S	-0.990531	-1.369959	-0.122071
	O	-0.906411	-2.041592	-1.379917
	O	-0.895752	-2.115805	1.093654
	O	3.871884	0.257303	0.144322
	C	4.291233	-1.084171	0.289719
	H	4.009238	-1.684379	-0.578563
	H	5.374040	-1.054179	0.370272
	H	3.873638	-1.535106	1.193055
	H	2.971032	2.591692	-0.150784
**7d** anion<br>Imaginary frequencies=0<br>SCF energy=-1105.5359483<br>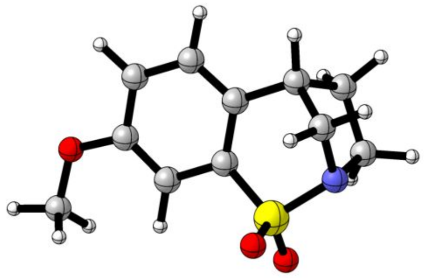	C	0.190443	-0.101581	0.012635
	C	1.583189	-0.471599	0.130897
	C	2.546575	0.491431	0.010131
	C	2.228771	1.837948	-0.244043
	C	0.861537	2.186495	-0.364516
	C	-0.144595	1.268341	-0.252570
	H	1.828501	-1.500642	0.342268
	H	0.596937	3.225950	-0.533917
	C	-1.591385	1.683700	-0.299435
	H	-1.676763	2.681389	-0.732368
	C	-2.410211	0.630172	-1.049413
	H	-3.441095	0.958745	-1.218072
	C	-2.247153	1.605046	1.095673
	H	-3.112317	2.271311	1.148183
	C	-2.690038	0.128176	1.211648
	H	-2.160911	-0.423510	1.983326
	H	-3.761306	0.048774	1.414464
	N	-2.422383	-0.489912	-0.098474
	H	-1.548625	1.893222	1.878569
	H	-1.979504	0.317591	-1.996776
	S	-0.968766	-1.337330	-0.175529
	O	-0.964199	-1.956262	-1.483922
	O	-0.955765	-2.259945	0.937648
	O	3.895927	0.232646	0.135161
	C	4.284414	-1.093269	0.352078
	H	3.955775	-1.750244	-0.460087
	H	5.373390	-1.095553	0.397432
	H	3.885187	-1.485715	1.293571
	H	3.020695	2.564676	-0.348430
**11b** neutral<br>Imaginary frequencies=0<br>SCF energy=-1217.6403092	C	0.133575	-0.616981	0.000003
	C	-1.072748	-1.298624	0.000098
	C	-2.239129	-0.562263	0.000041
	C	-2.176760	0.854236	-0.000150
	C	-0.957318	1.505066	-0.000321
	C	0.216742	0.759588	-0.000254
	H	-1.092064	-2.377636	0.000255
	H	-0.909137	2.582868	-0.000567

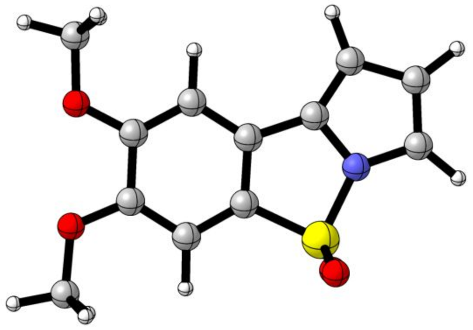

S	1.724880	-1.367360	0.000110
O	2.007806	-2.034343	1.228538
O	2.007055	-2.036148	-1.227502
O	-3.367602	1.475665	-0.000187
C	-3.395068	2.887329	-0.000350
H	-4.444793	3.165764	-0.000298
H	-2.912449	3.293585	0.891795
H	-2.912596	3.293377	-0.892671
O	-3.478964	-1.087741	0.000195
C	-3.607164	-2.493618	-0.000079
H	-3.154401	-2.933821	0.891964
H	-4.673866	-2.697696	-0.000245
H	-3.154173	-2.933487	-0.892168
N	2.466341	0.150537	-0.001115
C	1.596882	1.217315	-0.000504
C	3.762630	0.595968	-0.000003
C	2.344377	2.358337	0.000659
C	3.712649	1.959649	0.000476
H	4.594084	-0.086230	-0.000215
H	1.970280	3.368168	0.001309
H	4.568283	2.613629	0.000833

11b anion
Imaginary frequencies=0
SCF energy=-1217.6484802

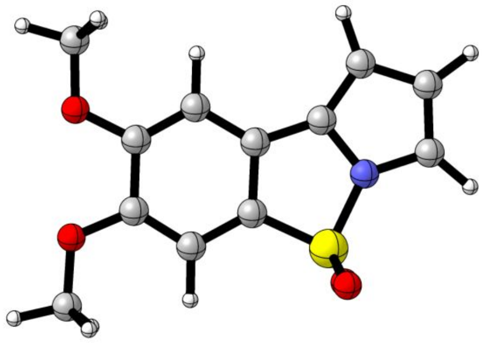

C	-0.164460	-0.628961	0.000329
C	1.070811	-1.301363	-0.000010
C	2.228806	-0.581215	0.000000
C	2.182032	0.856885	0.000240
C	0.983384	1.516419	0.000193
C	-0.229037	0.803877	0.000110
H	1.089931	-2.381613	-0.000270
H	0.953526	2.596874	0.000145
S	-1.711975	-1.345165	0.000260
O	-2.061168	-2.063477	-1.205884
O	-2.062641	-2.061335	1.207252
O	3.410691	1.469528	0.000434
C	3.429000	2.866068	0.000585
H	4.476778	3.162929	0.000814
H	2.938295	3.279793	-0.887942
H	2.937950	3.279602	0.889009
O	3.494146	-1.120068	-0.000322
C	3.585766	-2.513815	-0.000097
H	3.116535	-2.952511	-0.888368
H	4.647492	-2.756286	-0.000051
H	3.116502	-2.952204	0.888306
N	-2.439035	0.165140	-0.001315
C	-1.569081	1.252748	-0.000743
C	-3.753951	0.596638	-0.000821
C	-2.363277	2.395158	-0.000272
C	-3.715027	1.966035	-0.000627
H	-4.574422	-0.097546	-0.001251
H	-2.011697	3.413552	0.000166
H	-4.584057	2.606040	-0.000680

S	0.599822	-1.683857	-0.000061

15c neutral
Imaginary frequencies=0
SCF energy=-1371.2557935

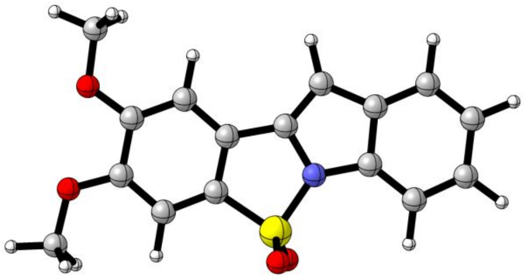

O	0.775515	-2.392142	-1.226506
O	0.775205	-2.392749	1.226076
N	1.542772	-0.297155	0.000385
C	2.899412	-0.030595	0.000164
C	3.990511	-0.883480	0.000227
C	5.241533	-0.301112	0.000121
C	5.398168	1.088133	-0.000088
C	4.305877	1.928767	-0.000151
C	3.028186	1.372554	-0.000023
C	1.702972	1.934940	-0.000151
H	1.449226	2.981698	-0.000424
C	0.833587	0.896726	0.000119
C	-0.596181	0.636676	0.000053
C	-0.869364	-0.714125	-0.000007
C	-2.159735	-1.218612	-0.000015
H	-2.331021	-2.284050	-0.000042
C	-3.210319	-0.324476	0.000003
O	-4.510111	-0.669544	-0.000007
C	-4.837305	-2.043612	-0.000078
H	-4.451450	-2.542853	-0.892246
H	-5.922132	-2.093221	-0.000107
H	-4.451494	-2.542934	0.892065
C	-2.950467	1.070611	0.000039
O	-4.042950	1.851836	0.000059
C	-3.872512	3.253558	0.000036
H	-3.337818	3.587914	-0.892272
H	-3.337878	3.587951	0.892366
H	-4.872837	3.676286	-0.000005
C	-1.652539	1.542445	0.000070
H	-1.451870	2.602521	0.000127
H	4.436811	3.003038	-0.000318
H	6.395540	1.506890	-0.000195
H	6.118897	-0.933685	0.000166
H	3.867274	-1.957349	0.000285

15c anion
Imaginary frequencies=0
SCF energy=-1371.2753029

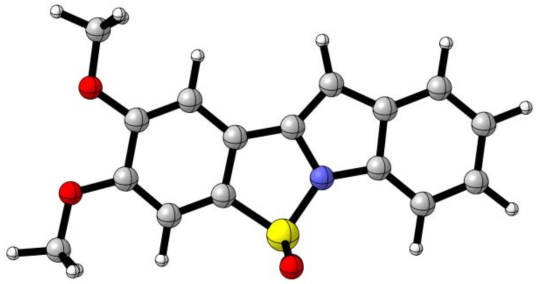

S	0.596271	-1.657627	0.000216
O	0.815934	-2.410387	-1.210390
O	0.814240	-2.409501	1.211690
N	1.525176	-0.273783	0.000219
C	2.887759	-0.019188	0.000056
C	3.961093	-0.878923	-0.000213
C	5.241185	-0.325705	-0.000557
C	5.411499	1.055549	-0.000306
C	4.327873	1.918927	0.000096
C	3.028225	1.398048	0.000189
C	1.731937	1.977206	0.000454
H	1.501480	3.029727	0.000597
C	0.803928	0.938581	0.000402
C	-0.571932	0.678175	-0.000197
C	-0.848563	-0.720636	-0.000815
C	-2.151208	-1.218077	-0.000813
H	-2.314693	-2.286166	-0.001116
C	-3.205612	-0.344227	-0.000562

O	-4.531778	-0.704098	-0.000847
C	-4.814132	-2.073158	0.000537
H	-4.410958	-2.572512	-0.887900
H	-5.898832	-2.166672	0.001073
H	-4.410140	-2.570822	0.889540
C	-2.954619	1.069483	-0.000183
O	-4.081095	1.842183	-0.000010
C	-3.912373	3.231892	0.000127
H	-3.371435	3.573583	-0.888616
H	-3.371683	3.573452	0.889073
H	-4.911701	3.663294	0.000029
C	-1.681721	1.561047	0.000024
H	-1.503326	2.626151	0.000421
H	4.484205	2.991200	0.000238
H	6.415068	1.465181	-0.000477
H	6.105335	-0.977270	-0.000937
H	3.811655	-1.950177	0.000110

18 neutral
Imaginary frequencies=0
SCF energy=-1412.9615406

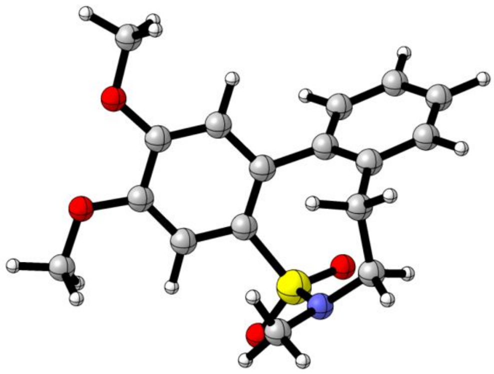

S	0.671342	-1.880237	-0.796615
O	1.759081	-1.370656	-1.567184
O	-0.135948	-2.954187	-1.290266
C	-0.449529	-0.557328	-0.402023
C	-1.805321	-0.895758	-0.377231
H	-2.078975	-1.906494	-0.631670
C	-2.768766	0.038120	-0.075917
O	-4.095006	-0.204114	-0.038704
C	-4.542364	-1.506255	-0.349227
H	-4.258848	-1.793345	-1.364616
H	-5.625457	-1.478004	-0.270756
H	-4.147229	-2.241982	0.355921
C	-2.370119	1.356648	0.211880
O	-3.359334	2.220769	0.503503
C	-3.023603	3.562653	0.783931
H	-2.529572	4.035676	-0.068159
H	-3.962845	4.070683	0.983053
H	-2.378405	3.636588	1.662757
C	-1.029237	1.678881	0.175239
H	-0.715643	2.688813	0.390356
C	-0.037377	0.739130	-0.127391
C	1.362790	1.242789	-0.179496
C	1.653089	2.229141	-1.118801
H	0.874309	2.543891	-1.801111
C	2.911988	2.788190	-1.212476
H	3.115022	3.542786	-1.960618
C	3.906695	2.372278	-0.345525
H	4.897651	2.803249	-0.399839
C	3.624265	1.402538	0.596332
H	4.400849	1.085599	1.282352
C	2.364366	0.821129	0.696206
C	2.157899	-0.255978	1.727351
H	1.149178	-0.215496	2.141682
H	2.841175	-0.067629	2.556555
C	2.440976	-1.673152	1.195434

	H	2.797826	-2.302109	2.011314
	H	3.226852	-1.635779	0.444516
	N	1.299290	-2.382979	0.631542
	C	0.390398	-3.006835	1.579100
	H	-0.335079	-3.608856	1.040431
	H	-0.140446	-2.278531	2.201376
	H	0.965883	-3.666040	2.228407
**18** anion<br>Imaginary frequencies=0<br>SCF energy=-1412.9580080<br>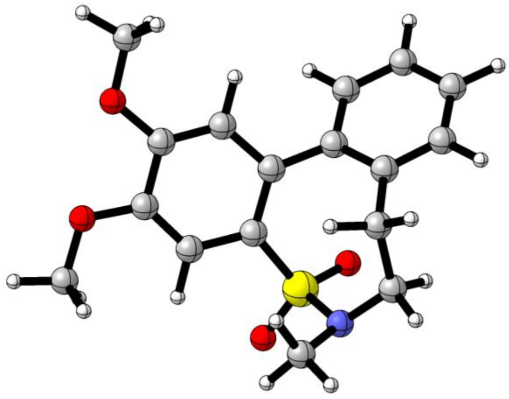				
	S	0.603914	-1.827029	-0.890060
	O	1.646472	-1.282589	-1.718111
	O	-0.215393	-2.909672	-1.383281
	C	-0.417401	-0.603781	-0.274702
	C	-1.795983	-0.955365	-0.137820
	H	-2.056316	-1.993182	-0.268533
	C	-2.763082	-0.029097	0.069053
	O	-4.104201	-0.297519	0.198737
	C	-4.496893	-1.632287	0.061464
	H	-4.230223	-2.035122	-0.921559
	H	-5.579753	-1.650889	0.177003
	H	-4.041379	-2.270250	0.827680
	C	-2.401402	1.354322	0.142568
	O	-3.440667	2.226991	0.360315
	C	-3.132924	3.588689	0.404737
	H	-2.663125	3.929090	-0.525183
	H	-4.075779	4.115939	0.543951
	H	-2.461468	3.830744	1.236687
	C	-1.085236	1.705634	0.050847
	H	-0.812060	2.743746	0.170308
	C	-0.024673	0.779153	-0.094768
	C	1.326444	1.312857	-0.081326
	C	1.594267	2.501257	-0.798159
	H	0.799922	2.925168	-1.398378
	C	2.833574	3.102142	-0.810469
	H	2.984688	4.002555	-1.395267
	C	3.886838	2.543525	-0.100906
	H	4.868959	2.998622	-0.103405
	C	3.643951	1.383999	0.624129
	H	4.445433	0.957619	1.219467
	C	2.406082	0.762947	0.653521
	C	2.250897	-0.432548	1.556881
	H	1.241068	-0.458940	1.970184
	H	2.930431	-0.296134	2.401608
	C	2.579260	-1.790780	0.906341
	H	3.049485	-2.440560	1.649297
	H	3.295143	-1.641043	0.100407
	N	1.455888	-2.547426	0.378107
	C	0.619724	-3.172649	1.382237
	H	-0.118207	-3.801223	0.889908
	H	0.092149	-2.453373	2.022092
	H	1.246858	-3.804128	2.014902

X-Ray Crystallographic Details for Compounds 15c and 17

2,3-Dimethoxybenzo[4,5]isothiazolo[2,3-*a*]indole 5,5-dioxide 15c:

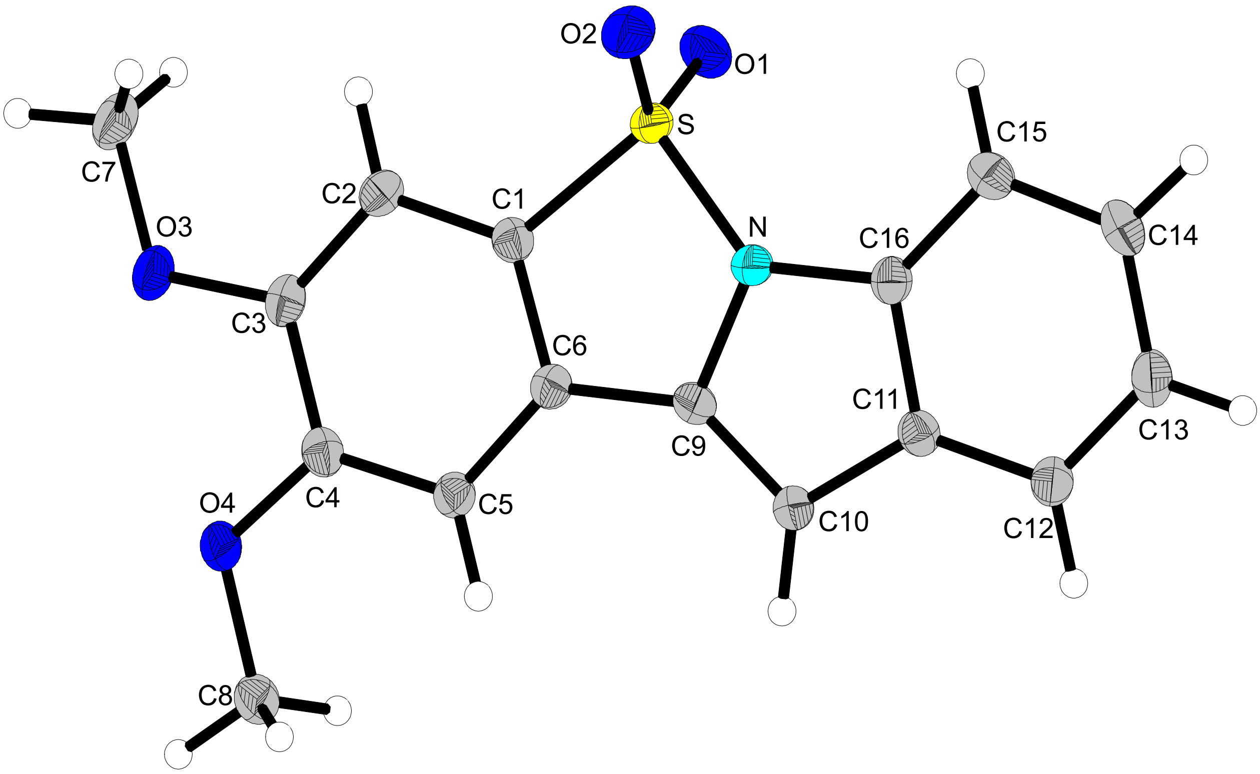

Formed by recrystallisation EtOAc (thermal ellipsoids are drawn on the 50% level)

Table S3. Crystal data and structure refinement for **15c** (eva87).

Identification code	eva87	
CCDC (www.ccdc.cam.ac.uk)	2170492	
Empirical formula	$C_{16} H_{13} N O_4 S$	
Formula weight	315.33	
Temperature	100(2) K	
Wavelength	1.54184 Å	
Crystal system	Monoclinic	
Space group	C2/c (#15)	
Unit cell dimensions	a = 14.5873(2) Å	α= 90°.
	b = 14.6099(2) Å	β= 98.266(1)°.
	c = 13.1442(2) Å	γ = 90°.
Volume	2772.18(7) Å^3	

Z	8
Density (calculated)	1.511 Mg/m^3
Absorption coefficient	2.253 mm^{-1}
F(000)	1312
Crystal size	0.251 x 0.101 x 0.069 mm^3
Theta range for data collection	4.306 to 76.771°.
Index ranges	–18<=h<=17, –18<=k<=18, –16<=l<=16
Reflections collected	16825
Independent reflections	2905 [R(int) = 0.0312]
Completeness to theta = 67.684°	100.0 %
Absorption correction	Gaussian
Max. and min. transmission	0.880 and 0.684
Refinement method	Full–matrix least–squares on F^2
Data / restraints / parameters	2905 / 0 / 201
Goodness–of–fit on F^2	1.034
Final R indices [I>2sigma(I)]	R1 = 0.0311, wR2 = 0.0852
R indices (all data)	R1 = 0.0347, wR2 = 0.0888
Extinction coefficient	n/a
Largest diff. peak and hole	0.281 and –0.447 e.Å^{-3}
Diffractometer	Rigaku XtaLab SuperNova X-ray diffractometer (XDA-018/09)

Table S4. Atomic coordinates (x 10^4) and equivalent isotropic displacement parameters (Å^{2}x 10^3) for **15c**. U(eq) is defined as one third of the trace of the orthogonalized U^{ij} tensor.

Atom	x	y	z	U(eq)
S	3193(1)	8619(1)	1092(1)	18(1)
O(1)	3810(1)	8675(1)	339(1)	27(1)
O(2)	2224(1)	8618(1)	714(1)	28(1)
C(1)	3522(1)	7772(1)	2017(1)	17(1)
C(2)	3455(1)	6835(1)	1835(1)	18(1)
C(3)	3796(1)	6260(1)	2637(1)	19(1)
O(3)	3787(1)	5331(1)	2582(1)	24(1)
C(7)	3406(1)	4950(1)	1610(1)	26(1)
C(4)	4201(1)	6628(1)	3602(1)	19(1)
O(4)	4530(1)	5995(1)	4317(1)	23(1)
C(8)	5024(1)	6329(1)	5268(1)	22(1)
C(5)	4246(1)	7568(1)	3761(1)	18(1)
C(6)	3900(1)	8150(1)	2952(1)	17(1)

C(9)	3886(1)	9146(1)	2920(1)	17(1)
N	3443(1)	9445(1)	1959(1)	20(1)
C(10)	4171(1)	9889(1)	3504(1)	18(1)
C(11)	3916(1)	10690(1)	2884(1)	18(1)
C(12)	4016(1)	11629(1)	3066(1)	22(1)
C(13)	3692(1)	12234(1)	2284(1)	24(1)
C(14)	3271(1)	11925(1)	1323(1)	24(1)
C(15)	3154(1)	10994(1)	1116(1)	22(1)
C(16)	3476(1)	10395(1)	1908(1)	18(1)

Table S5. Bond lengths [Å] and angles [°] for **15c**.

S–O(2)	1.4290(10)
S–O(1)	1.4335(10)
S–N	1.6636(11)
S–C(1)	1.7522(13)
C(1)–C(6)	1.3876(17)
C(1)–C(2)	1.3905(18)
C(2)–C(3)	1.3843(18)
C(2)–H(2)	0.9500
C(3)–O(3)	1.3593(16)
C(3)–C(4)	1.4254(18)
O(3)–C(7)	1.4300(17)
C(7)–H(7A)	0.9800
C(7)–H(7B)	0.9800
C(7)–H(7C)	0.9800
C(4)–O(4)	1.3558(16)
C(4)–C(5)	1.3887(18)
O(4)–C(8)	1.4360(16)
C(8)–H(8A)	0.9800
C(8)–H(8B)	0.9800
C(8)–H(8C)	0.9800
C(5)–C(6)	1.3983(17)
C(5)–H(5)	0.9500
C(6)–C(9)	1.4551(18)
C(9)–C(10)	1.3600(18)
C(9)–N	1.4035(16)
N–C(16)	1.3896(16)
C(10)–C(11)	1.4442(17)

C(10)–H(10)	0.9500
C(11)–C(12)	1.3965(18)
C(11)–C(16)	1.4165(17)
C(12)–C(13)	1.3858(19)
C(12)–H(12)	0.9500
C(13)–C(14)	1.398(2)
C(13)–H(13)	0.9500
C(14)–C(15)	1.393(2)
C(14)–H(14)	0.9500
C(15)–C(16)	1.3894(18)
C(15)–H(15)	0.9500
O(2)–S–O(1)	116.63(7)
O(2)–S–N	110.37(6)
O(1)–S–N	109.52(6)
O(2)–S–C(1)	113.46(6)
O(1)–S–C(1)	112.47(6)
N–S–C(1)	91.52(6)
C(6)–C(1)–C(2)	123.60(12)
C(6)–C(1)–S	111.54(9)
C(2)–C(1)–S	124.80(10)
C(3)–C(2)–C(1)	117.28(12)
C(3)–C(2)–H(2)	121.4
C(1)–C(2)–H(2)	121.4
O(3)–C(3)–C(2)	124.46(12)
O(3)–C(3)–C(4)	115.12(12)
C(2)–C(3)–C(4)	120.42(12)
C(3)–O(3)–C(7)	115.88(11)
O(3)–C(7)–H(7A)	109.5
O(3)–C(7)–H(7B)	109.5
H(7A)–C(7)–H(7B)	109.5
O(3)–C(7)–H(7C)	109.5
H(7A)–C(7)–H(7C)	109.5
H(7B)–C(7)–H(7C)	109.5
O(4)–C(4)–C(5)	124.47(12)
O(4)–C(4)–C(3)	114.78(11)
C(5)–C(4)–C(3)	120.75(12)
C(4)–O(4)–C(8)	117.05(10)
O(4)–C(8)–H(8A)	109.5
O(4)–C(8)–H(8B)	109.5

H(8A)–C(8)–H(8B)	109.5
O(4)–C(8)–H(8C)	109.5
H(8A)–C(8)–H(8C)	109.5
H(8B)–C(8)–H(8C)	109.5
C(4)–C(5)–C(6)	118.92(12)
C(4)–C(5)–H(5)	120.5
C(6)–C(5)–H(5)	120.5
C(1)–C(6)–C(5)	119.03(12)
C(1)–C(6)–C(9)	111.74(11)
C(5)–C(6)–C(9)	129.22(11)
C(10)–C(9)–N	108.84(11)
C(10)–C(9)–C(6)	141.25(12)
N–C(9)–C(6)	109.90(10)
C(16)–N–C(9)	109.86(10)
C(16)–N–S	134.18(9)
C(9)–N–S	114.66(9)
C(9)–C(10)–C(11)	107.19(11)
C(9)–C(10)–H(10)	126.4
C(11)–C(10)–H(10)	126.4
C(12)–C(11)–C(16)	118.50(12)
C(12)–C(11)–C(10)	133.43(12)
C(16)–C(11)–C(10)	108.07(11)
C(13)–C(12)–C(11)	118.90(12)
C(13)–C(12)–H(12)	120.6
C(11)–C(12)–H(12)	120.6
C(12)–C(13)–C(14)	121.53(13)
C(12)–C(13)–H(13)	119.2
C(14)–C(13)–H(13)	119.2
C(15)–C(14)–C(13)	121.14(12)
C(15)–C(14)–H(14)	119.4
C(13)–C(14)–H(14)	119.4
C(16)–C(15)–C(14)	116.78(12)
C(16)–C(15)–H(15)	121.6
C(14)–C(15)–H(15)	121.6
C(15)–C(16)–N	130.88(12)
C(15)–C(16)–C(11)	123.14(12)
N–C(16)–C(11)	105.98(11)

Symmetry transformations used to generate equivalent atoms

Table S6. Anisotropic displacement parameters ($Å^2$x 10^3) for **15c**. The anisotropic displacement factor exponent takes the form: $-2\pi^2[h^2 a^{*2}U^{11} + ... + 2 h k a^* b^* U^{12}]$

Atom	U^{11}	U^{22}	U^{33}	U^{23}	U^{13}	U^{12}
S	20(1)	17(1)	16(1)	–1(1)	–1(1)	1(1)
O(1)	37(1)	26(1)	20(1)	2(1)	8(1)	3(1)
O(2)	23(1)	27(1)	32(1)	0(1)	–9(1)	1(1)
C(1)	16(1)	18(1)	18(1)	1(1)	2(1)	0(1)
C(2)	16(1)	19(1)	20(1)	–3(1)	2(1)	–1(1)
C(3)	17(1)	15(1)	25(1)	–1(1)	4(1)	0(1)
O(3)	27(1)	15(1)	29(1)	–2(1)	1(1)	0(1)
C(7)	32(1)	17(1)	31(1)	–6(1)	3(1)	–1(1)
C(4)	16(1)	18(1)	22(1)	3(1)	2(1)	0(1)
O(4)	26(1)	16(1)	24(1)	3(1)	–3(1)	0(1)
C(8)	22(1)	20(1)	23(1)	3(1)	–1(1)	1(1)
C(5)	17(1)	17(1)	19(1)	0(1)	2(1)	–1(1)
C(6)	14(1)	17(1)	18(1)	0(1)	2(1)	0(1)
C(9)	17(1)	19(1)	16(1)	2(1)	1(1)	2(1)
N	25(1)	16(1)	16(1)	0(1)	–2(1)	1(1)
C(10)	21(1)	16(1)	17(1)	1(1)	2(1)	0(1)
C(11)	19(1)	18(1)	18(1)	1(1)	4(1)	0(1)
C(12)	28(1)	18(1)	20(1)	0(1)	5(1)	–3(1)
C(13)	29(1)	17(1)	27(1)	2(1)	8(1)	–1(1)
C(14)	25(1)	21(1)	25(1)	7(1)	5(1)	3(1)
C(15)	23(1)	22(1)	20(1)	3(1)	1(1)	2(1)
C(16)	19(1)	16(1)	20(1)	0(1)	3(1)	1(1)

Table S7. Hydrogen coordinates (x 10^4) and isotropic displacement parameters ($Å^2$x 10^3) for **15c**.

Atom	x	y	z	U(eq)
H(2)	3187	6600	1186	22
H(7A)	3779	5144	1085	40
H(7B)	2768	5163	1423	40
H(7C)	3412	4280	1657	40
H(8A)	5529	6731	5126	33

H(8B)	5281	5812	5690	33
H(8C)	4599	6674	5638	33
H(5)	4507	7811	4408	22
H(10)	4481	9882	4191	22
H(12)	4303	11850	3714	26
H(13)	3756	12874	2405	29
H(14)	3061	12358	802	28
H(15)	2869	10779	465	26

Table S8. Torsion angles [°] for **15c**.

O(2)–S–C(1)–C(6)	–118.76(9)
O(1)–S–C(1)–C(6)	106.15(10)
N–S–C(1)–C(6)	–5.74(9)
O(2)–S–C(1)–C(2)	64.03(12)
O(1)–S–C(1)–C(2)	–71.06(12)
N–S–C(1)–C(2)	177.05(11)
C(6)–C(1)–C(2)–C(3)	–0.52(18)
S–C(1)–C(2)–C(3)	176.36(9)
C(1)–C(2)–C(3)–O(3)	–179.67(12)
C(1)–C(2)–C(3)–C(4)	–0.39(18)
C(2)–C(3)–O(3)–C(7)	1.01(19)
C(4)–C(3)–O(3)–C(7)	–178.30(11)
O(3)–C(3)–C(4)–O(4)	0.98(16)
C(2)–C(3)–C(4)–O(4)	–178.37(11)
O(3)–C(3)–C(4)–C(5)	–179.49(11)
C(2)–C(3)–C(4)–C(5)	1.17(19)
C(5)–C(4)–O(4)–C(8)	–5.31(18)
C(3)–C(4)–O(4)–C(8)	174.21(11)
O(4)–C(4)–C(5)–C(6)	178.47(11)
C(3)–C(4)–C(5)–C(6)	–1.02(18)
C(2)–C(1)–C(6)–C(5)	0.66(18)
S–C(1)–C(6)–C(5)	–176.59(9)
C(2)–C(1)–C(6)–C(9)	179.59(11)
S–C(1)–C(6)–C(9)	2.34(13)
C(4)–C(5)–C(6)–C(1)	0.14(17)
C(4)–C(5)–C(6)–C(9)	–178.58(12)
C(1)–C(6)–C(9)–C(10)	–175.40(16)

C(5)–C(6)–C(9)–C(10)	3.4(3)
C(1)–C(6)–C(9)–N	3.18(14)
C(5)–C(6)–C(9)–N	–178.03(12)
C(10)–C(9)–N–C(16)	2.44(14)
C(6)–C(9)–N–C(16)	–176.62(10)
C(10)–C(9)–N–S	171.29(9)
C(6)–C(9)–N–S	–7.77(13)
O(2)–S–N–C(16)	–71.10(14)
O(1)–S–N–C(16)	58.62(14)
C(1)–S–N–C(16)	173.14(13)
O(2)–S–N–C(9)	123.60(10)
O(1)–S–N–C(9)	–106.69(10)
C(1)–S–N–C(9)	7.83(10)
N–C(9)–C(10)–C(11)	–1.11(14)
C(6)–C(9)–C(10)–C(11)	177.47(15)
C(9)–C(10)–C(11)–C(12)	179.32(14)
C(9)–C(10)–C(11)–C(16)	–0.55(14)
C(16)–C(11)–C(12)–C(13)	–0.56(19)
C(10)–C(11)–C(12)–C(13)	179.57(13)
C(11)–C(12)–C(13)–C(14)	–0.2(2)
C(12)–C(13)–C(14)–C(15)	0.6(2)
C(13)–C(14)–C(15)–C(16)	–0.08(19)
C(14)–C(15)–C(16)–N	177.98(13)
C(14)–C(15)–C(16)–C(11)	–0.75(19)
C(9)–N–C(16)–C(15)	178.41(13)
S–N–C(16)–C(15)	12.6(2)
C(9)–N–C(16)–C(11)	–2.70(14)
S–N–C(16)–C(11)	–168.51(10)
C(12)–C(11)–C(16)–C(15)	1.09(19)
C(10)–C(11)–C(16)–C(15)	–179.01(12)
C(12)–C(11)–C(16)–N	–177.91(11)
C(10)–C(11)–C(16)–N	1.99(13)

Symmetry transformations used to generate equivalent atoms

2,3-Dimethoxy-6-methyl-7,8-dihydro-6*H*-dibenzo[e,g][1,2]thiazocine 5,5-dioxide 17:

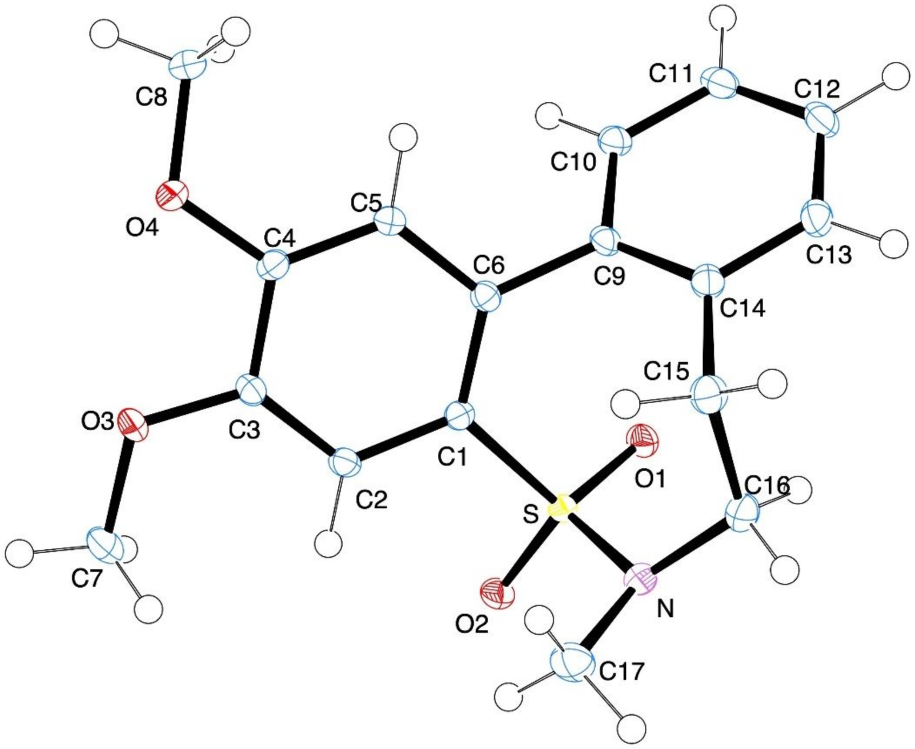

Formed by recrystallisation from acetone (thermal ellipsoids are drawn on the 30% level). The synthesis of this compound was described in *Tetrahedron Lett*. **2008**, *49*, 7187.

Table S9. Crystal data and structure refinement for **17** (eva35).

Identification code	eva35
CCDC (www.ccdc.cam.ac.uk)	2170491
Empirical formula	$C_{17} H_{19} N O_4 S$
Formula weight	333.39
Temperature	100(2) K
Wavelength	0.71073 Å
Crystal system	Monoclinic
Space group	P 21/n
Unit cell dimensions	a = 12.9789(11) Å α = 90°.
	b = 7.4268(6) Å β = 96.527(2)°.
	c = 16.1148(13) Å γ = 90°.
Volume	1543.3(2) Å^3
Z	4
Density (calculated)	1.435 Mg/m^3
Absorption coefficient	0.230 mm^{-1}
F(000)	704
Crystal size	0.80 x 0.60 x 0.40 mm^3

Theta range for data collection	1.91 to 30.51°.
Index ranges	-18<=h<=18, -10<=k<=10, -22<=l<=22
Reflections collected	17110
Independent reflections	4648 [R(int) = 0.0239]
Completeness to theta = 30.00°	99.6 %
Absorption correction	Semi-empirical from equivalents
Max. and min. transmission	0.9135 and 0.7569
Refinement method	Full-matrix least-squares on F^2
Data / restraints / parameters	4648 / 0 / 284
Goodness-of-fit on F^2	1.051
Final R indices [I>2sigma(I)]	R1 = 0.0394, wR2 = 0.1016
R indices (all data)	R1 = 0.0452, wR2 = 0.1051
Largest diff. peak and hole	0.630 and -0.312 e.Å^{-3}
Diffractometer	Rigaku XtaLab SuperNova X-ray diffractometer (XDA-018/09)

Table S10. Atomic coordinates (x 10^4) and equivalent isotropic displacement parameters (Å^{2}x 10^3) for **17**. U(eq) is defined as one third of the trace of the orthogonalized U^{ij} tensor.

Atom	x	y	z	U(eq)
S	3217(1)	6128(1)	462(1)	12(1)
O(1)	2211(1)	5673(1)	687(1)	16(1)
O(2)	3888(1)	4706(1)	248(1)	16(1)
C(1)	3918(1)	7358(1)	1279(1)	12(1)
C(2)	5001(1)	7178(2)	1359(1)	13(1)
C(3)	5600(1)	7953(2)	2029(1)	13(1)
O(3)	6650(1)	7860(1)	2181(1)	20(1)
C(7)	7172(1)	6767(2)	1632(1)	25(1)
C(4)	5105(1)	8856(1)	2645(1)	13(1)
C(8)	5266(1)	10081(2)	4017(1)	17(1)
C(5)	4042(1)	9071(2)	2545(1)	13(1)
C(6)	3423(1)	8368(2)	1848(1)	12(1)
C(9)	2296(1)	8773(1)	1785(1)	14(1)
C(10)	1767(1)	8359(2)	2472(1)	16(1)
C(11)	724(1)	8763(2)	2472(1)	19(1)
C(12)	198(1)	9626(2)	1786(1)	21(1)
C(13)	720(1)	10072(2)	1110(1)	19(1)
C(14)	1769(1)	9657(2)	1092(1)	16(1)
C(15)	2272(1)	10199(2)	329(1)	18(1)
C(16)	2172(1)	8767(2)	-373(1)	19(1)

N	3022(1)	7446(1)	-354(1)	16(1)
C(17)	3953(1)	8096(2)	-697(1)	23(1)

Table S11. Bond lengths [Å] and angles [°] for **17**.

S-O(1)	1.4341(9)
S-O(2)	1.4359(8)
S-N	1.6356(10)
S-C(1)	1.7675(11)
C(1)-C(6)	1.3966(15)
C(1)-C(2)	1.4035(15)
C(2)-C(3)	1.3819(15)
C(2)-H(2)	0.965(17)
C(3)-O(3)	1.3589(13)
C(3)-C(4)	1.4112(15)
O(3)-C(7)	1.4271(15)
C(7)-H(7A)	0.94(2)
C(7)-H(7B)	0.949(18)
C(7)-H(7C)	0.998(19)
C(4)-O(4)	1.3580(13)
C(4)-C(5)	1.3802(15)
O(4)-C(8)	1.4307(14)
C(8)-H(8A)	0.940(17)
C(8)-H(8B)	0.961(17)
C(8)-H(8C)	0.947(16)
C(5)-C(6)	1.4058(15)
C(5)-H(5)	0.937(16)
C(6)-C(9)	1.4856(15)
C(9)-C(10)	1.4022(16)
C(9)-C(14)	1.4037(16)
C(10)-C(11)	1.3865(16)
C(10)-H(10)	0.949(16)
C(11)-C(12)	1.3881(18)
C(11)-H(11)	0.979(17)
C(12)-C(13)	1.3860(19)
C(12)-H(12)	0.973(18)
C(13)-C(14)	1.3991(16)
C(13)-H(13)	0.988(18)

C(14)-C(15)	1.5109(17)
C(15)-C(16)	1.5470(17)
C(15)-H(15A)	0.999(18)
C(15)-H(15B)	0.966(17)
C(16)-N	1.4742(15)
C(16)-H(16A)	0.940(18)
C(16)-H(16B)	0.987(18)
N-C(17)	1.4663(16)
C(17)-H(17A)	0.954(18)
C(17)-H(17B)	0.96(2)
C(17)-H(17C)	0.987(19)
O(1)-S-O(2)	118.78(5)
O(1)-S-N	106.42(5)
O(2)-S-N	106.83(5)
O(1)-S-C(1)	109.88(5)
O(2)-S-C(1)	106.50(5)
N-S-C(1)	107.98(5)
O(4)-C(4)-C(3)	115.22(10)
C(13)-C(12)-H(12)	120.0(10)
C(11)-C(12)-H(12)	120.2(10)
C(16)-C(15)-H(15B)	106.9(10)
H(15A)-C(15)-H(15B)	106.9(14)
N-C(16)-C(15)	116.28(10)
N-C(16)-H(16A)	106.0(11)
C(15)-C(16)-H(16A)	109.6(11)
N-C(16)-H(16B)	108.6(10)
C(15)-C(16)-H(16B)	108.8(10)
H(16A)-C(16)-H(16B)	107.2(15)
C(17)-N-C(16)	114.85(10)
C(17)-N-S	116.20(8)
C(16)-N-S	117.46(8)
N-C(17)-H(17A)	108.7(11)
N-C(17)-H(17B)	111.4(12)
H(17A)-C(17)-H(17B)	110.2(15)
N-C(17)-H(17C)	106.1(11)
H(17A)-C(17)-H(17C)	109.2(15)
H(17B)-C(17)-H(17C)	111.2(16)

Symmetry transformations used to generate equivalent atoms

Table S12. Anisotropic displacement parameters ($Å^2$x 10^3) for **17**. The anisotropic displacement factor exponent takes the form: $-2\pi^2[h^2 a^{*2}U^{11} + ... + 2 h k a^* b^* U^{12}]$.

Atom	U^{11}	U^{22}	U^{33}	U^{23}	U^{13}	U^{12}
S	12(1)	14(1)	12(1)	-2(1)	1(1)	0(1)
O(1)	12(1)	19(1)	18(1)	-2(1)	2(1)	-2(1)
O(2)	16(1)	16(1)	17(1)	-4(1)	3(1)	2(1)
C(1)	13(1)	13(1)	11(1)	-1(1)	1(1)	0(1)
C(2)	13(1)	15(1)	13(1)	0(1)	4(1)	1(1)
C(3)	11(1)	15(1)	14(1)	0(1)	3(1)	0(1)
O(3)	10(1)	30(1)	20(1)	-8(1)	2(1)	1(1)
C(7)	14(1)	35(1)	27(1)	-11(1)	4(1)	4(1)
C(4)	14(1)	14(1)	12(1)	-1(1)	1(1)	-1(1)
O(4)	13(1)	26(1)	14(1)	-6(1)	1(1)	-1(1)
C(8)	18(1)	20(1)	12(1)	-4(1)	2(1)	2(1)
C(5)	13(1)	14(1)	13(1)	-2(1)	2(1)	1(1)
C(6)	12(1)	12(1)	13(1)	1(1)	2(1)	1(1)
C(9)	12(1)	13(1)	16(1)	-4(1)	2(1)	1(1)
C(10)	15(1)	16(1)	16(1)	-4(1)	3(1)	1(1)
C(11)	15(1)	20(1)	22(1)	-8(1)	6(1)	-1(1)
C(12)	13(1)	22(1)	26(1)	-10(1)	1(1)	3(1)
C(13)	16(1)	18(1)	21(1)	-6(1)	-3(1)	5(1)
C(14)	15(1)	14(1)	17(1)	-4(1)	0(1)	1(1)
C(15)	20(1)	16(1)	18(1)	2(1)	1(1)	3(1)
C(16)	19(1)	21(1)	15(1)	1(1)	-1(1)	4(1)
N	16(1)	20(1)	12(1)	1(1)	2(1)	2(1)
C(17)	23(1)	26(1)	20(1)	5(1)	8(1)	0(1)

Table S13. Hydrogen coordinates (x 10^4) and isotropic displacement parameters ($Å^2$x 10^3) for **17**.

Atom	x	y	z	U(eq)
H(2)	5335(13)	6470(20)	965(11)	25(4)
H(7A)	6957(14)	5560(30)	1687(12)	31(5)
H(7B)	7886(14)	6870(30)	1834(11)	29(4)

H(7C)	7052(14)	7190(20)	1041(12)	30(5)
H(8A)	4841(13)	9170(20)	4196(10)	19(4)
H(8B)	5844(13)	10280(20)	4434(11)	23(4)
H(8C)	4903(13)	11170(20)	3896(10)	19(4)
H(5)	3730(13)	9700(20)	2956(10)	18(4)
H(10)	2130(12)	7780(20)	2941(10)	18(4)
H(11)	369(13)	8440(20)	2955(11)	23(4)
H(12)	-525(14)	9970(20)	1790(10)	25(4)
H(13)	336(14)	10710(20)	634(11)	27(4)
H(15A)	3016(14)	10510(30)	493(10)	27(4)
H(15B)	1938(13)	11280(20)	96(11)	23(4)
H(16A)	2141(13)	9340(20)	-895(11)	26(4)
H(16B)	1513(14)	8110(20)	-358(11)	26(4)
H(17A)	4465(14)	7170(20)	-639(11)	25(4)
H(17B)	4219(15)	9170(30)	-418(12)	38(5)
H(17C)	3743(14)	8320(30)	-1296(12)	32(5)

Table S14. Torsion angles [°] for **17**.

O(1)-S-C(1)-C(6)	-25.50(11)
O(3)-C(3)-C(4)-O(4)	-2.45(15)
C(4)-C(5)-C(6)-C(1)	3.27(16)
C(4)-C(5)-C(6)-C(9)	-176.64(10)
C(1)-C(6)-C(9)-C(10)	126.90(12)
C(9)-C(10)-C(11)-C(12)	-1.31(17)
C(10)-C(11)-C(12)-C(13)	0.04(18)
C(11)-C(12)-C(13)-C(14)	0.80(18)
C(12)-C(13)-C(14)-C(9)	-0.36(17)
C(12)-C(13)-C(14)-C(15)	-179.95(11)
C(10)-C(9)-C(14)-C(13)	-0.89(16)
C(6)-C(9)-C(14)-C(13)	-176.38(10)
C(10)-C(9)-C(14)-C(15)	178.68(10)
C(6)-C(9)-C(14)-C(15)	3.19(17)
C(13)-C(14)-C(15)-C(16)	-88.34(13)
C(9)-C(14)-C(15)-C(16)	92.09(13)
C(14)-C(15)-C(16)-N	-91.33(13)
C(15)-C(16)-N-C(17)	-80.01(13)
C(15)-C(16)-N-S	62.08(13)
O(1)-S-N-C(17)	179.77(9)

O(2)-S-N-C(17)	-52.37(10)
C(1)-S-N-C(17)	61.85(10)
O(1)-S-N-C(16)	38.19(10)
O(2)-S-N-C(16)	166.04(8)
C(1)-S-N-C(16)	-79.74(10)

Symmetry transformations used to generate equivalent atoms
